# Foraging models explain human exploration in uncertain tasks

**DOI:** 10.1038/s41467-026-75773-4

**Published:** 2026-07-31

**Authors:** Meriam Zid, Veldon-James Laurie, Jorge Ramírez-Ruiz, Alix Lavigne-Champagne, Akram Shourkeshti, Dameon C. Harrell, Alexander B. Herman, R. Becket Ebitz

**Affiliations:** 1 Department of Neuroscience, University of Montreal, Montreal, QC Canada; 2 Department of Psychiatry, University of Minnesota, Minneapolis, MN USA

**Keywords:** Decision, Learning algorithms, Human behaviour

## Abstract

Different fields have fundamentally different models of how decisions are made. Psychology and neuroscience tend to assume that decisions are made by calculating and comparing the values of all options. In ethology, conversely, decisions tend to be binary regardless of the number of options: Decision-makers calculate the value of continuing to exploit some option and explore only when this value drops below a threshold. Because these fields use incompatible methods, it remains unclear which view better describes human decision-making. We find that humans use compare-to-threshold computations even in classic compare-alternative tasks. Because the reinforcement-learning models typically used in the cognitive and brain sciences depend on compare-alternative computations, we also develop a compare-to-threshold foraging model. Compared to previous models, the foraging model better fits participant behavior, predicts the tendency to repeat choices, and predicts held-out participants that were almost impossible under traditional compare-alternative models. These results suggest that humans use compare-to-threshold computations in more environments than were previously known.

## Abstract (web summary)

It is often assumed that people decide by comparing the values of all available options. Here, the authors challenge this by showing that a foraging model inspired by natural behavior only tracks the value of exploration and explains choices more accurately than classic theories.

## Introduction

Because the world is only imperfectly observable and changes frequently, many of the decisions we make are uncertain. How do we make good decisions in the presence of this uncertainty? The conventional view in the cognitive and brain sciences is that we (1) estimate the subjective utility or value of each option being considered and then (2) compare alternative option values to make the best possible decision^1–5^. This compare-alternatives perspective is ubiquitous in psychology and neuroscience. For example, nearly all reinforcement learning (RL) models, from the Rescorla-Wagner model^1^ to models like Q-Learning and SARSA^6,7^, assume that the value of choosing each alternative option is compared in some way in order to select the best action^1,4,6^. These models, as well as the compare-alternatives perspective more broadly, shape the way that tasks are designed in these fields. For example, because we assume that values must be compared against each other, laboratory tasks often present all options simultaneously^8–11^. Similarly, because the reward prediction error in RL models functions to track changing mean values, reward contingencies in reward learning tasks tend to both increase and decrease over time^12–15^—in contrast to the decay of exploited resources often seen in natural environments.

While the compare-alternatives view is widely influential, it is not universally accepted. In ethology, for example, foraging models instead assume that we (1) calculate the value of one exploited option and (2) compare this one value against a threshold to decide whether to continue exploiting or try something new^16,17^. This compare-to-threshold view grew out of natural environments, where the value of an exploited resource tends to decay over time, and options are encountered sequentially, rather than in parallel. As a result, although compare-to-threshold models have begun seeing some use among human cognitive scientists^18–23^, these studies tend to develop tasks that mirror foraging assumptions: they introduce reward contingencies that decay at a predictable rate (rather than changing unpredictably) and/or options that are encountered in sequence (rather than being presented simultaneously)^18–23^. In short, the compare-alternatives and compare-to-threshold views of sequential decision-making use incomparable formalisms to make sense of behavior in incompatible environments. As a result, we do not know which view best describes human decision-making in standard laboratory tasks.

Here, we asked whether human decision-making was better described as a compare-to-threshold process or as a compare-alternatives process in a classic testbed of decision-making under uncertainty from the reinforcement learning (RL) literature: a restless *k*-armed bandit task. We found that human behavior more closely resembled compare-to-threshold computations than compare-alternatives computations. This insight is difficult to reconcile with traditional RL algorithms from psychology and neuroscience, which model action selection as a compare-alternatives process. Therefore, we developed a compare-to-threshold sequential decision-making model (foraging-RL model) that is inspired by foraging theory but incorporates delta-rule reward prediction errors from RL. We compared this new foraging-RL model with multiple variations of the compare-alternatives RL models (traditional RL)^4^ across five independent experiments that manipulated the uncertainty of the unchosen options, the demands of the environment, and the setting in which the experiments were performed. We found that the foraging-RL model was a better fit for the participants’ decisions in all experiments, outperformed multiple variations on traditional RL models, and better predicted the participants’ tendency to repeat choices on both the individual and group level. Together, our findings suggest that humans are more likely to use compare-to-threshold computations than previously understood. Across multiple variations of a task commonly used as a testbed for compare-alternatives computations, people tended, on average, to use foraging-like algorithms.

## Results

### Participants performed above chance and used complex strategies

**Fig. 1:**
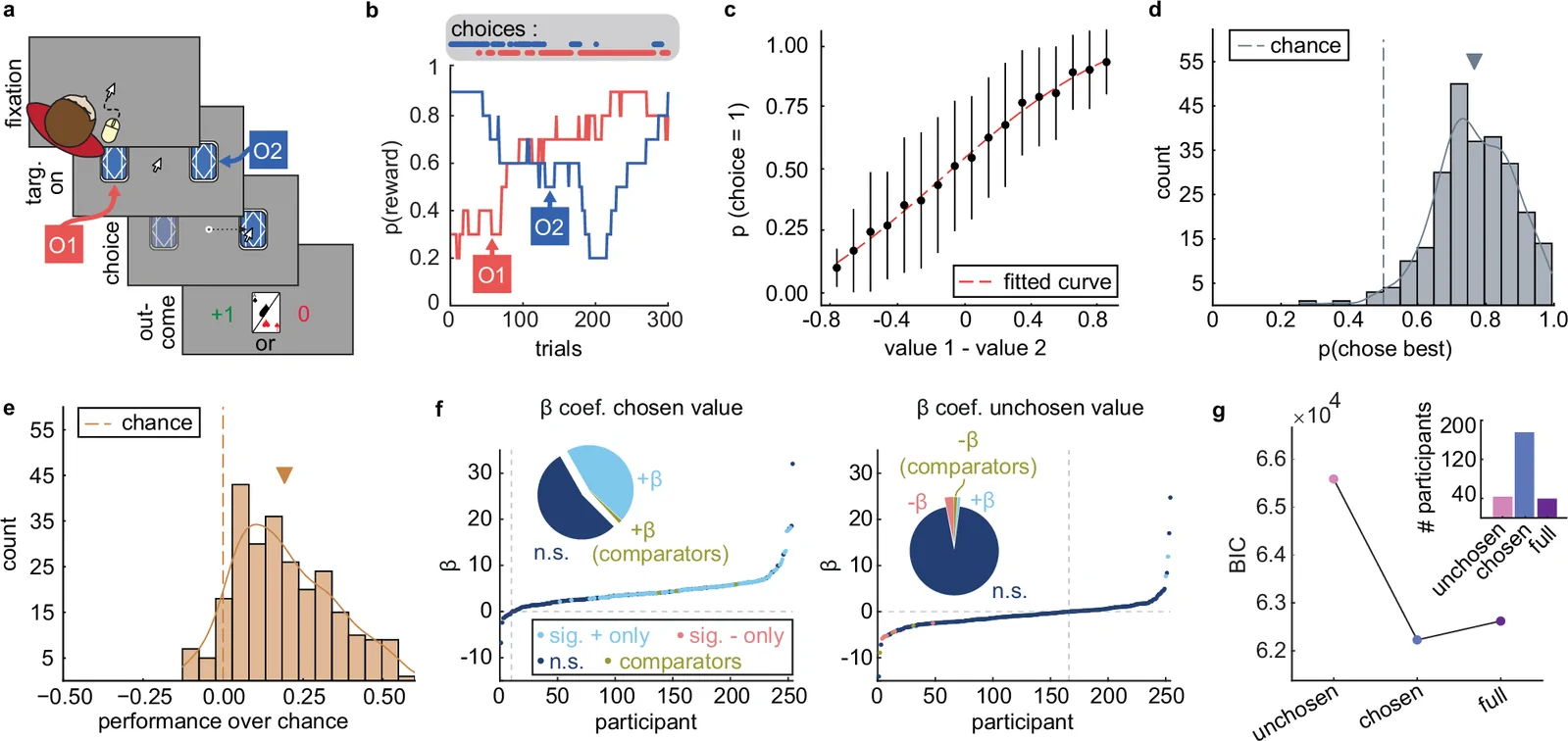
Experiment 1 task and baseline behavior. **a** Participants (*n* = 258) chose between 2 face-down decks of playing cards that were composed of aces and 2’s. Participants were told that the proportion of aces and 2’s in each deck would change over time and that decks could be good, bad, or mediocre right now, but would not stay that way the whole time. Participants got a point (and $0.02) for each ace they found, but no points for 2’s. **b** The reward probability of each option (red trace = option 1; blue trace = option 2) from an example reward schedule, with participant choices along the top (red dots = option 1; blue dots = option 2). **c** Average probability of choosing option 1 as a function of the difference in the objective reward probability (“value”) between option 1 and 2. Error bars = standard error of the mean (SEM) across participants. **d** Distribution of the number of trials in which each participant chose the objectively best option. Dotted line = chance, caret = mean across participants. **e** Same as **d**, for the percent of trials in which participants were rewarded, normalized as percent of chance performance. **f** Predicting choice from the value of chosen and unchosen options. Beta coefficients for (*V*(chosen_*t*−1_)) (left) and *V*(unchosen_*t*−1_) (right). Each circle represents a single participant, sorted by coefficient value. The dashed horizontal line is at zero (no effect). Participants with a significant positive betas are light blue, significant negative betas in red, and non-significant betas in dark blue. Gold circles identify “comparators” who had a significant positive weight for the chosen option and negative weight for unchosen option value. Significance is Holm-Bonferroni corrected within predictor (*α* = 0.05). Insets illustrate the proportion of participants in whom each term was significant, colored by sign and identifying all “comparators”. **g** Sum of BIC for each model (*V*(unchosen_*t*−1_)-only, *V*(chosen_*t*−1_)-only, and the full model which contains both terms). Inset: The number of individual participants best fit by each model, based on the Bayesian Information Criterion (BIC).

In Experiment 1, participants on the Amazon MTurk platform (258 people, 120 female, 2 other or non-reporting) performed a classic sequential decision-making task known as a restless *k*-armed bandit (Fig. 1a; refs. ^6,12,14,15,24,25^). The participants were asked to repeatedly choose between 2 options, each of which was associated with some probability of reward. A 2-option version of the task was chosen here because we believed that minimizing the number of options would encourage compare-alternatives computations via reducing the computational demands of this strategy. Reward probabilities changed unpredictably over time and independently across options (Fig. 1b) and were not cued to the participants. The only way to infer the value of an option was to sample it. This task naturally encourages decision-makers to alternate between exploiting valuable options and exploring uncertain alternatives because the latter could become better at any time. This task is a classic testbed for evaluating RL models in psychology, neuroscience, and Artificial Intelligence (AI)^4,12,14,15,24,26,27^.

Participants were generally good at the task despite the uncertainty (Fig. 1c). They chose the objectively best option 76.6% of the time (±11.5% STD; Fig. 1d) and received rewards 19.2% more frequently than expected by chance (±15.3% STD; Fig. 1e). Note that 4/258 participants [1.6%] were excluded from this and several further analyses because they chose only one option. Participants were more likely to repeat choices than to switch (switching on only 19.9% of trials, ±14.5% STD). They were also more likely to repeat after reward (win-stay = 93.3%, ±11.1% STD) and switch after no-reward (lose-shift = 39.2%, ±21.0% STD). However, no participants followed a deterministic win-stay-lose-shift rule (all participants either win-shifted or lose-stayed at least 5% of the time).

### Participants largely ignored the value of the unchosen option

The compare-to-threshold view predicts that the probability of choosing an option is only determined by the prior value of that option, not the value of any alternative options. Therefore, choices should be entirely insensitive to the value of unchosen alternatives. By contrast, decisions should depend on the difference between chosen and unchosen values in the compare-alternatives view. To determine which view better described participants’ choices here, we developed a generalized linear model (GLM) that predicted choice from the reward probabilities of the previously chosen option (*V*(chosen_*t*−1_)) and the previously unchosen option (*V*(unchosen_*t*−1_); see Methods). We found that the value of the chosen option was a positive predictor of the decision to repeat a choice in roughly half the participants (Fig. 1f, left: sig. positive in 118/254 participants). However, the value of the unchosen option had minimal influence (Fig. 1f, right: sig. positive in 2/254 participants; sig. negative in 11/254 participants). Further, of the 11 participants who had a negative *V*(unchosen_*t*−1_) term, only 3 also had a significant positive *V*(chosen_*t*−1_) term. This meant that only 3 out of 258 participants (1.2%) were value “comparators” (Fig. 1f, right) in the sense predicted by the compare-alternatives view, while the vast majority of participants (98.8%) were not. Model comparison revealed that a model which included only *V*(chosen_*t*−1_) outperformed a full model (containing both *V*(chosen_*t*−1_) and *V*(unchosen_*t*−1_)) (Fig. 1g). The *V*(chosen_*t*−1_)—only model was also better fit to the majority of individual participants (better fit in 174/254 participants; *wBIC* of full and *V*(chosen_*t*−1_)—only models relative to *V*(unchosen_*t*−1_)—only model (both < 0.001)). These results hold true even when using subjective values estimated via an Ideal Bayesian Learner (IBL) model (see Supplementary Fig. S1). Together, these results suggest that the value of the previously chosen option was the primary driver of choice, with minimal influence from unchosen option values, consistent with the compare-to-threshold view.

### Human behavior better resembles the fingerprint of compare-to-threshold decisions

**Fig. 2:**
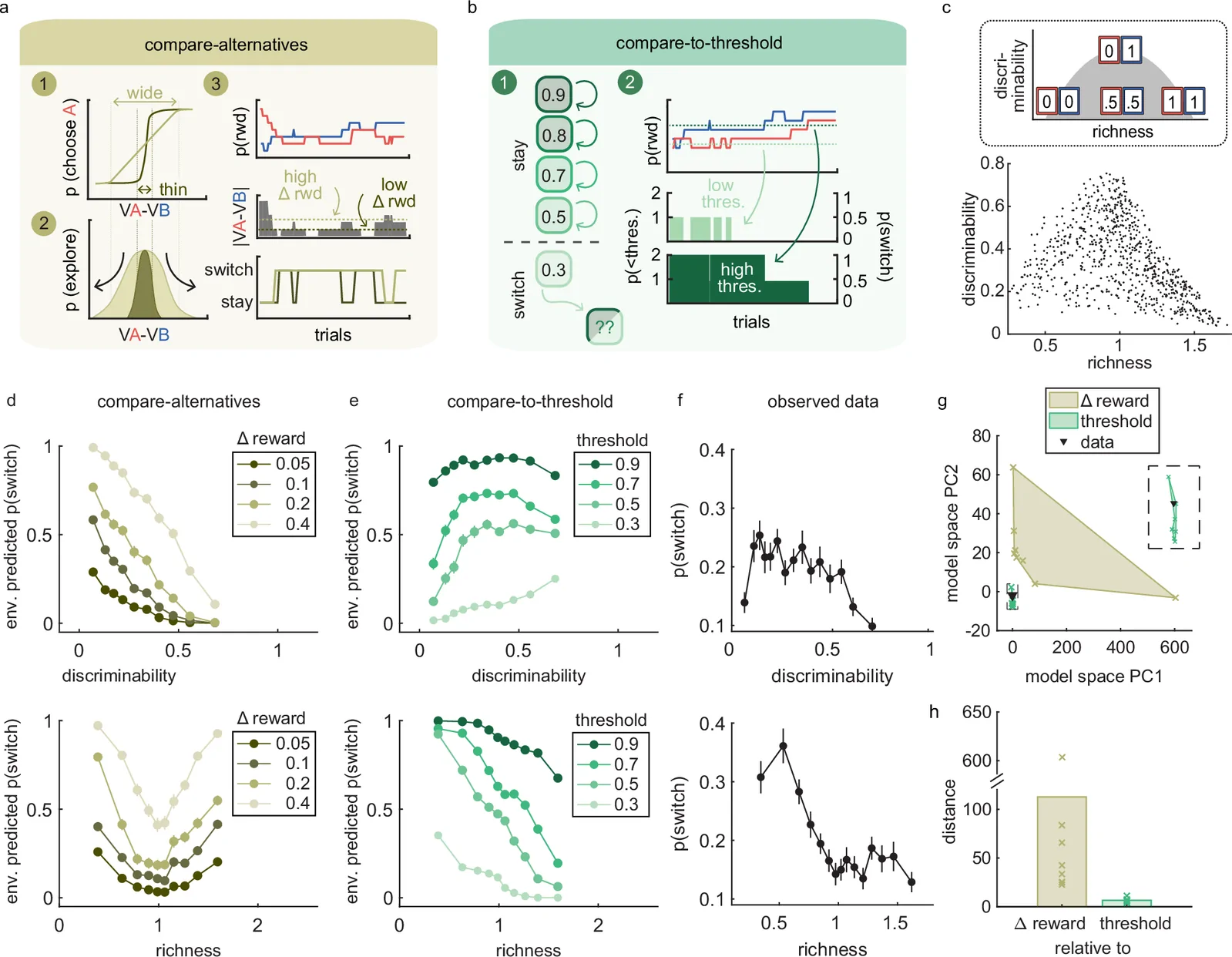
Dissociable signatures of compare-to-threshold and compare-alternatives decisions to explore as a function of environmental statistics. **a** In compare-alternatives decision-making, exploration is driven by value comparison noise. With a softmax decision rule, noise is larger (and thus exploration is more frequent) when values are close together. Note that the breadth of this high-exploration regime varies with the inverse temperature of the softmax. If we consider a typical reward walk, this means that switching should occur most frequently when value differences are low and the decision-maker is in the exploratory regime. Switching is related to discriminability here. **b** In compare-to-threshold decision-making, the decision to stay depends only on the value of one focal option. If this value drops below a threshold, the algorithm switches. This means that switching rarely occurs when all options are above threshold and occurs in proportion to the number of options that are below threshold otherwise. Switching is related to richness here. **c** Top: Schematic relationship between richness and discriminability for 4 example pairs of reward probabilities. Bottom: Discriminability as a function of richness in the reward walks in Experiment 1. Each dot represents discriminability and richness in a 100-trial bin (see Methods). **d** Probability that the difference in option values was below some Δ reward as a function of discriminability (top) and richness (bottom) for 4 values of Δ reward (shades of gold, errorbar = SEM). This is the environment predicted p(switch) under compare-alternatives. **e** Probability that any option value was below a threshold as a function of discriminability (top) and richness (bottom) for 4 threshold values (shades of green, errorbar = SEM). This is the predicted p(switch) under compare-to-threshold. **f** Probability of switching within each 100 trial bin for all participants in Experiment 1 as a function of discriminability (top) and richness (bottom; *n* = 774; errorbar = SEM). **g** First 2 PCs of the multivariate feature space of compare-alternatives fingerprints (gold) and compare-to-threshold fingerprints (green) (see Methods). Bounds encompass all values of the Δ reward and threshold parameters. x = each parameter value, black caret = humans. **h** Distance between humans and every simulated datapoint in the full 7-dimensional space. x = individual parameter values.

The compare-alternatives and compare-to-threshold views also differ in their predictions about how people should decide to explore as a function of the environment (Fig. 2a, b). Specifically, the compare-alternatives and compare-to-threshold views predict that switching will be most frequent in different situations because there are different mechanisms for exploration in the two cases. The compare-alternatives view predicts that people are most likely to explore when it is difficult to discern which option is the best. Switching would therefore occur most frequently when the option values are close together (i.e., when the Δ reward between the options is small; Fig. 2a). This happens most often in ambiguous environments, rather than discriminable environments. Conversely, the compare-to-threshold view predicts that people explore whenever an exploited option falls below a threshold (Fig. 2b). Switching would therefore occur most frequently when the value of any option is more likely to fall below a threshold. This happens most often when all option values are low, in poor environments rather than rich environments, when no option can fall below the threshold. Note that this is true even without assuming a compare-to-threshold algorithm that calculates richness: compare-to-threshold choices will inevitably depend on richness as a byproduct of the fact that options are more likely to be above threshold in rich environments. In short, switching should depend on different statistics of the environment in compare-to-threshold decision-making versus compare-alternatives decision-making.

These insights imply that we should be able to extract distinct fingerprints of these two strategies as a function of the environment. Regardless of implementation, switching probability in compare-to-threshold decision-making should depend on richness (the sum of values), while it should depend on discriminability (the differences between values) in compare-alternatives decision-making. However, in any bounded environment, the sum of values and the difference between values will be related (Fig. 2c; i.e., any time outcomes are probabilistic or there are floor and/or ceilings on outcome magnitude). When richness is high (value of all options is close to their maximum) or low (value of all options close to their minimum), discriminability can only be low. However, when richness is at intermediate levels, discriminability can range from low (e.g., all values around their midpoint) to high (e.g., a mix of probabilities close to min and max). As a consequence of these bounds, compare-alternatives decision-making should have a U-shaped relationship with richness, with minimal switching at intermediate levels where discriminability is highest (Fig. 2d, bottom). By contrast, compare-to-threshold decision-making will have an inverted U-shaped relationship with discriminability, driven by the statistical relationship between richness and discriminability (Fig. 2e, top), and both strategies have a unique fingerprint as a function of both variables (Fig. 2d, e).

We found that the participants switched options the most at intermediate, rather than low levels of discriminability (Fig. 2f, top), consistent with compare-to-threshold computations (Fig. 2e, top). The participants were also less likely to switch in rich environments, compared to poor ones (Fig. 2f, bottom), again consistent with compare-to-threshold computations (Fig. 2e, bottom). To quantify the relationship between participants’ data and the compare-alternatives and compare-to-threshold fingerprints, we calculated the probability of each strategy switching frequently as a function of discriminability and richness (i.e., environment predicted p(switch); Fig. 2d, e; see Methods). We then measured the slope and curvature of the relationship to discriminability and richness under each hypothesis (Fig. 2g, h; see Methods). Human switching was sensitive to discriminability and richness in a way that better resembled compare-to-threshold decision making, both in terms of the individual features (Fig. S2a–c) and in a multivariate feature space that considered all features simultaneously (Figs. 2g, h and S2d, e; median distance to Δ reward in the full feature space = 38.05, range = [22.61, 603.84]; to threshold = 6.23, range = [3.70, 11.25]; significant Wilcoxon rank sum test, *W*(15) = 92, *p* < 0.001, *r* = 0.50; see Methods). While the human data was not a perfect match for either fingerprint, it better resembled the fingerprint of compare-to-threshold decision-making than the fingerprint of compare-alternatives decision-making.

### Compare-alternatives agents perform better than compare-to-threshold agents on the task

**Fig. 3:**
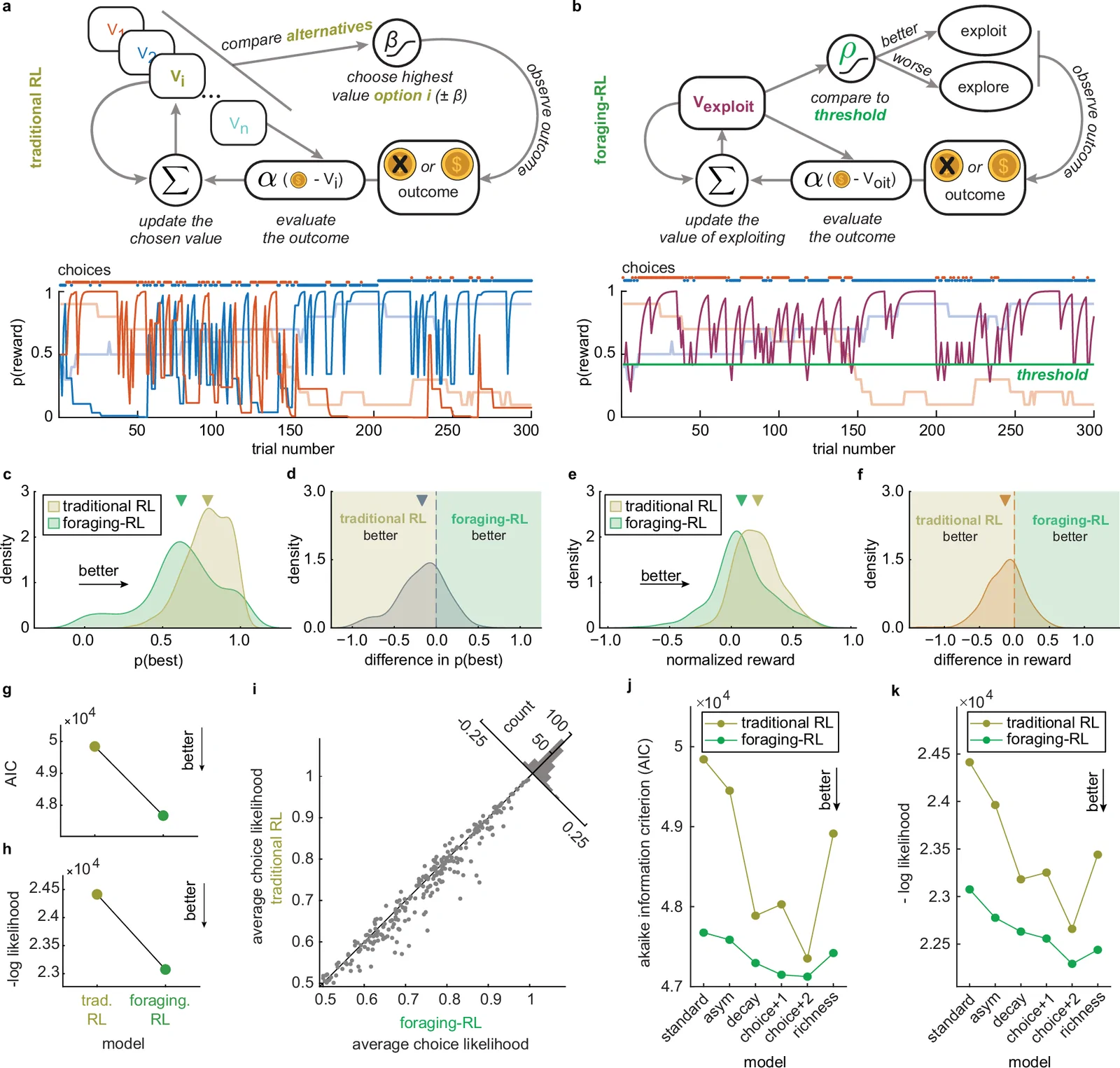
Traditional RL and foraging-RL models produce different choice patterns, performance and quality fits to the data. **a** The traditional RL model estimates the value of choosing each individual option through a delta-rule update. The value of choosing alternative options are then compared and the highest value is selected. Bottom: Simulated data from the traditional RL model, fit to example participant from Experiment 1 and run on the corresponding reward schedule. The choices (colored dots) and estimated values (colored traces) of the model are shown, along with the objective reward schedule (pale traces). **b** The foraging-RL model estimates the value of staying with the currently exploited option through a delta-rule update. The value of exploitation is then compared to a threshold in order to decide whether to stay and continue exploiting or to switch. bottom: Simulated data from the foraging-RL model, fit to an example participant from Experiment 1 and run on the corresponding reward schedule. The choices (colored dots) and exploitation value (purple trace) of the compare-to-threshold foraging-RL model are shown, along with the objective reward probabilities (pale traces). The horizontal green line is the threshold. **c** Distribution of the probability of choosing the objectively best option for both foraging-RL (green) and traditional RL (gold) agents (caret = mean). **d** Difference between the distributions seen in (**c**). **e** Distribution of the normalized average reward received by both foraging-RL (green) and traditional RL (gold) agents (caret = mean). **f** Difference between the distributions seen in (**e**) (caret = mean). **g** The Akaike information criteria (AIC) of the foraging-RL and traditional RL model fits to all data from Experiment 1 (*n* = 254). **h** The negative log-likelihood of the traditional RL and foraging-RL models fits (*n* = 254). **i** Average choice likelihood for each participant under the foraging-RL (*x*-axis) and the traditional RL model (*y*-axis). Inset: Distribution of differences in choice likelihood across participants of Experiment 1. **j** The Akaike information criteria of the extensions of traditional RL (gold) and foraging-RL models (green). **k** The negative log-likelihood of the extensions of the traditional RL (gold) and foraging-RL models (green).

Participants may have looked more compare-to-threshold in this task because some aspect of the task design encouraged the participants to use compare-to-threshold, rather than compare-alternatives computations. To determine if this was the case, we developed a sequential decision-making agent based on compare-to-threshold computations (foraging-RL model) and compared its performance on the task against a traditional compare-alternatives RL agent (traditional RL model; Rescorla-Wagner^6,7^). Where a traditional compare-alternatives RL agent estimates the value of choosing each option and compares them (*V*_1_, …, *V*_*n*_; Fig. 3a), our foraging-RL agent updates the value of exploiting the last chosen action via the same delta-rule computations used in traditional RL agents (*V*_exploit_; Fig. 3b). It then compares this value against a fixed threshold to decide whether to continue exploiting or to explore the alternatives at random (foraging-RL model; Fig. 3b). When there are only 2 options, this means deterministically switching to the only alternative.

Simulating both agents with the same reward schedules experienced by participants revealed that the environment did not advantage foraging-RL over traditional RL. In fact, traditional RL agents outperformed foraging-RL agents, regardless of whether performance was defined as the probability of choosing the objectively best option (significant Wilcoxon signed-rank test: *W*(257) = 4703, *p* < 0.001, average difference = −0.180, difference range = [−0.987, 0.327]; Fig. 3c, d) or as the probability of reward (significant Wilcoxon signed-rank test: *W*(257) = 5226, *p* < 0.001, average difference = −0.140, difference range = [−1.209, 0.373]; Fig. 3e, f). The task not only did not encourage compare-to-threshold decision-making, but the participants would have sacrificed a reward to adopt this strategy.

### Foraging-RL models as a class better predict participant decisions

We next fit a series of computational models to behavior (see Methods). We first compared a simple 2-parameter reinforcement learning model ("standard traditional RL”; Rescorla-Wagner^1,6^) and a simple 3-parameter formulation of the Foraging-RL model ("standard foraging-RL”). We found that the standard Foraging-RL model was a better fit to participant behavior than the standard RL model (Fig. 3g, h; standard foraging-RL: AIC = 47,675, 3 parameters by 254 participants = 762 parameters; log-likelihood = −23,075, standard traditional RL: AIC = 49,840, relative AIC weight  < 0.001, 2 parameters per participant, 508 total, log-likelihood = −24,413; 76,200 total trials), with both models substantially outperforming a 1-parameter Win-Stay-Lose-Shift model (WSLS: AIC = 84,740, log-likelihood = −42,116). The standard foraging-RL model was also a better fit on an individual basis in comparison to standard traditional RL (Fig. 3i; standard foraging-RL: median individual AIC = 170.73, median individual log-likelihood = −82.36; standard traditional RL: median individual AIC = 177.44, median individual log-likelihood = −86.72; significant difference in AIC paired Wilcoxon signed rank test: *W*(253) = 11097, *p* < 0.001, average difference = −8.530, difference range = [−112.807, 32.757]). Choice likelihoods of both models were stable through time within participants (Fig. S3a, b). Together, these results suggest that a foraging-like compare-to-threshold mechanism may better describe the participants’ choices in this task.

A variety of extensions and alternatives are known to outperform the standard 2-parameter traditional RL model, and it remained unclear whether any of these would fit behavior better than the foraging-RL model. Therefore, we next compared the foraging-RL model with 10 additional models: 4 common extensions of the traditional RL model (asymmetrical learning, value decay, and 2 forms of choice history dependence), 2 dynamic Bayesian models and a free-fitting exponential filter (see Supplementary Fig. S4e), and 3 extensions meant to address the specific limitations of the traditional RL model identified here (see Methods). At the group level, every extended traditional RL model explained behavior better than the standard traditional RL model (Fig. 3j, k and Supplementary Fig. S4). The foraging-RL was a better fit than all but 1 extended traditional RL model (Fig. 3j, k, Supplementary Fig. S4 and Supplementary Table 1). Foraging-RL fit better than a traditional RL model that incorporated asymmetrical learning from wins and losses ("asymmetrical traditional RL”^24,28–30^; AIC = 49,450, AIC weight relative to standard foraging-RL model < 0.001, 762 parameters, log-likelihood = −23,963), decay in the value of unchosen options ("decaying traditional RL”^15,31–33^; AIC = 47,887, relative AIC weight < 0.001, 762 parameters, log-likelihood = −23,181), and a simple form of choice-history dependence with 1 added parameter ("history-kernel 1 traditional RL”^4,34^; AIC = 48,028, AIC weight relative to standard foraging-RL model < 0.001, 762 parameters, log-likelihood = −23,252). Foraging-RL also outperformed 3 extensions to RL models that were developed to account for our behavioral observations: an RL model in which values are explicitly normalized by richness (see Methods; richness; Supplementary Fig. S4e and Supplementary Table 1), a repetition bonus model that implements a tradeoff between repeating the last option and making decisions based on relative value (see Methods; repetition bonus; Supplementary Fig. S4e and Supplementary Table 1), and a model that combined richness normalization with the best formulation of choice-history dependence (see Methods; richness + choice + 2; Supplementary Fig. S4e and Supplementary Table 1). The only extended traditional RL model that outperformed the standard foraging-RL model was a 2-parameter choice-history dependent model (history-kernel 2 traditional RL^4,35^; AIC = 47,351, relative weight of the standard foraging-RL model < 0.001, log-likelihood = −22,660, 1016 parameters). In short, the simplest foraging-RL model provided an explanation for behavior that was comparable to or better than all but the most complex traditional RL models.

Critically, any one traditional RL model could have performed better than the standard foraging-RL model for two reasons: either because it benefits from the compare-alternatives architecture or because decades of incremental engineering were ultimately able to overcome some of the limitations of that architecture. Therefore, we next compared foraging-RL models with traditional RL models as a class. We developed an equivalent version of the foraging-RL model for each extension of the traditional RL model (see Methods; Fig. 3j, k and Supplementary Fig. S4e). Note that our goal here was not to build the best foraging-RL model we could, but instead to offer a fair comparison-to-threshold version of each common variant of the traditional RL model. We found that every extended foraging-RL model outperformed the equivalent traditional RL model at the group level (Figs. 3j, k and S4 and Supplementary Table 2), suggesting that compare-to-threshold models outperformed compare-alternatives models as a class. Across all models tested, the single best model was the 2-parameter history kernel foraging-RL model, which outperformed the 2-parameter history kernel traditional RL model, which had also been the best in its class at both the group level (Supplementary Table 2) and across individual participants (foraging-RL model: median individual AIC = 168.64, median individual log-likelihood = −79.32, traditional RL model: median individual AIC = 168.85, median log-likelihood = −80.43, significant difference in history kernel 2 AIC paired Wilcoxon signed-rank test, *W*(253) = 13,145,  *p* = 0.009, average difference = −0.893, difference range = [−129.724, 46.013]). Ultimately, the compare-to-threshold foraging-RL models consistently outperformed compare-alternatives models as a class and provided a better fit to the participants’ decisions as a group. At the individual level, we assessed model fit by summing the AIC values for each participant within each class (i.e., each traditional RL extension vs. its corresponding foraging-RL extension) and compared them across participants. We found that foraging-RL as a class better accounts for participants' decisions (significant Wilcoxon signed-rank test, *W*(253) = 13,098, *p* = 0.008, average difference = −35.856, difference range = [−862.534, 222.997]), suggesting again that compare-to-threshold models were, as a class, a better fit to behavior than traditional, compare-alternatives RL models.

### Foraging-RL better explained the participants’ tendency to repeat

We next asked which aspects of behavior the foraging-RL model described that the traditional RL model was unable to account for. We previously found that choices are more repetitive than traditional RL models, at least in humans and other primates^15,36^. This may explain why many extensions of the traditional RL model make the model more repetitive. This is most obvious in the case of the choice history kernel models: added parameters increase the probability of repeating the previous choice. In decay models, similarly, repetition increases as the value of the alternative decays without selection. Even in the asymmetrical learning models, learning more from wins than losses tends to increase repetition^37,38^. Therefore, we first asked if the foraging-RL model naturally better captured the repetitiveness of the participants’ choices.

**Fig. 4:**
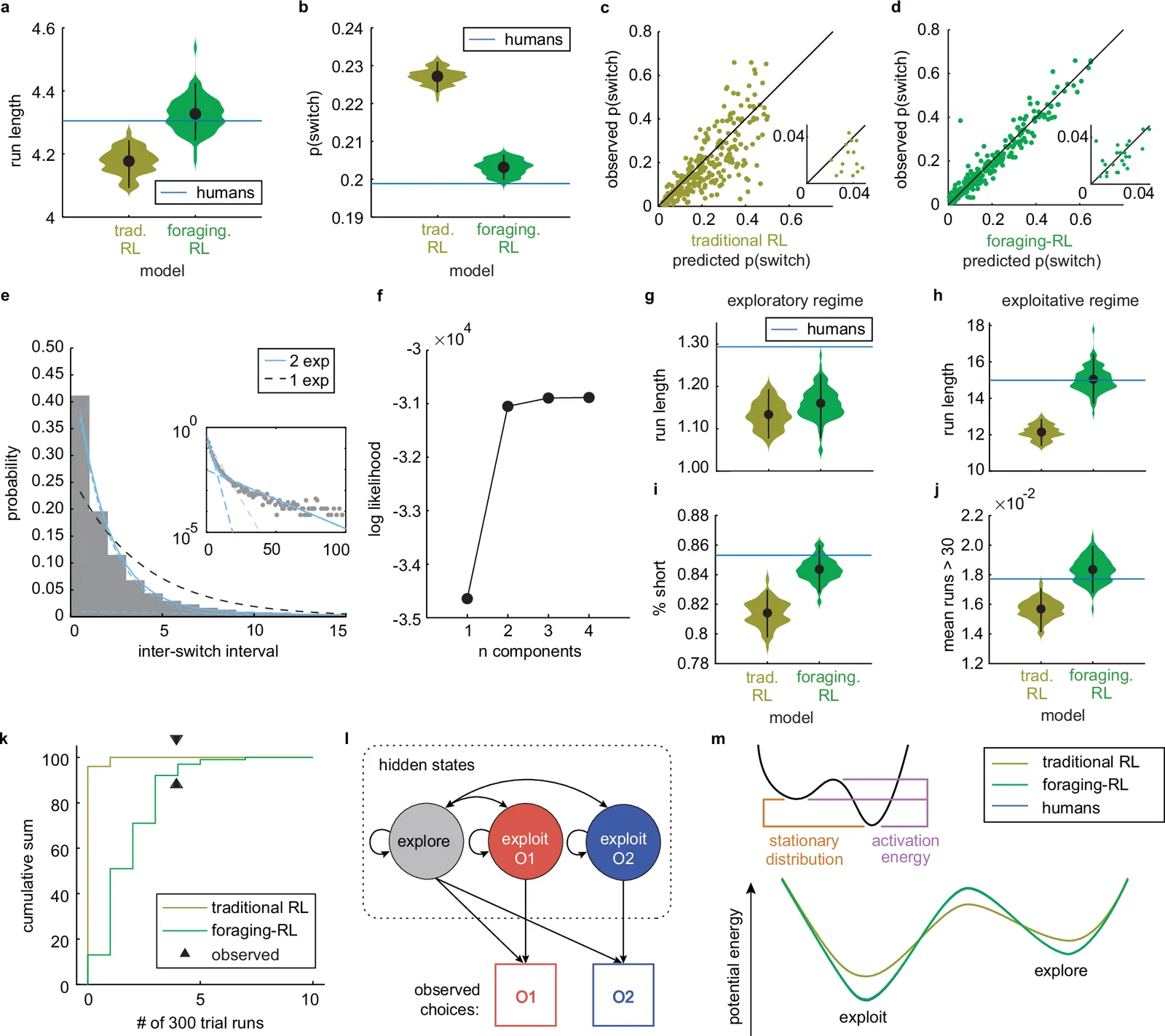
Foraging-RL and traditional RL predict different choice dynamics. **a** Average number of trials between switch decisions (i.e., run length) of traditional RL (gold) and foraging-RL (green) agents in simulated Experiment 1. Blue line = average for Experiment 1 participants; circle = mean; black line = IQR, *n* = 100. Same conventions as in (**a**), (**b**) and (**g**–**j**). **b** Probability of switch. **c** Human observed probability of switch as a function of traditional RL predicted probability of switch (inset = zoomed in). **d** Human observed probability of switch as a function of foraging-RL predicted probability of switch (inset = zoomed in). **e** Distribution of choice run lengths of humans in Experiment 1. If the probability of switching were fixed, run lengths would be exponentially distributed (black dotted line). A mixture of two exponential distributions (blue line) suggests 2 distinct probabilities of switching. Dotted blue lines show each mixing distribution, one slow-switching (i.e., exploitative regime) and another fast-switching (i.e., exploratory regime). Inset= Same data presented on a logarithmic scale. **f** Log-likelihoods for different mixture models containing a range of 1 to 4 exponential distributions. **g** Average run length in the exploratory regime. **h** Average run length in the exploitative regime. **i** Proportion of exploratory regimes. **j** Proportion of very long exploitative regimes exceeding 30 consecutive trials. **k** Four participants were excluded from some analyses because they chose the same option 300 trials in a row. To determine the expected number of these no-switch participants under each model, 100 datasets were generated from the distribution of fitted parameters (each with 254 simulated participants). Here, we plot the cumulative percent of simulations (*y*-axis) as a function of the number of no-switch participants (*x*-axis). Black caret = proportion of no-switch participants in Experiment 1. **l** A Hidden Markov model (HMM) was used to infer the underlying internal state of the participants on each trial from their sequence of choices. The model included 3 hidden states, 2 exploitative states corresponding to each option, and an exploratory state, where participants could choose any of the options. **m** Overlaid state dynamic landscapes for foraging-RL (green), traditional RL (gold) agents, and Experiment 1 participants (blue).

We simulated data from traditional RL and foraging-RL agents that were matched to the participants’ environment and parameters (see Methods; and the 1-parameter WSLS: Supplementary Fig. S5). We then compared the level of repetitiveness in these simulated datasets with that of the participants. In participants, the average run length (length of same-choice sequences: 4.31 trials) was close to our expectation under foraging-RL (mean = 4.33 trials, 95% CI = [4.24, 4.43]) but greater than any observation from the traditional RL simulations (mean = 4.18 trials, 95% CI = [4.09, 4.24]; Fig. 4a). The foraging-RL was also more accurate in predicting how often individual participants would switch (i.e., the inverse of choice run lengths; Fig. 4b–d; foraging-RL, mean sum of squared errors (MSSE) = 0.72, 95% CI across simulations = [0.53, 0.94], traditional RL, MSSE = 2.78 (95% CI = [2.54, 3.01]; significantly greater in traditional RL; 2-sided paired *t*-test: *t*(1,253) = 6.44, *p* < 0.001, *d* = 0.40, 95% CI = [0.56%, 1.05%]). The average probability of switching under the traditional RL model was 22.7% (95% CI = [22.3%, 23.1%]) and under the foraging-RL it was 20.3% (95% CI = [20.0%, 20.7%]). The participants switched 20.0% of the time, less frequently than any of the samples from the traditional RL model, but not the foraging-RL. The traditional RL model systematically predicted that individual participants should switch more than they did (2.8% more, *t*(253) = 4.72, *p* < 0.001, *d* = 0.3, 95% CI across individuals = [1.7%, 4.0%]), while also failing to describe participants who switched very frequently (Fig. 4c inset). Conversely, the foraging-RL did not have a significant bias towards over- or under-estimating individual participants (predicted 0.4% more on average, *t*(253) = 1.66, *p* = 0.1, *d* = 0.3, 95% CI = [−0.09% to 1%]) and captured both extremes of the population (4d, inset). In short, compared to the traditional RL model, the foraging-RL better predicted the participants’ tendency to switch on both the individual and group levels: it was both more precise and less biased.

Given that the foraging-RL model was especially adept at predicting participants at the extremes (Fig. 4c, d), it remained possible that this model outperformed the traditional RL model solely because it was a better fit for participants who did not understand or engage with the task. However, there was no significant relationship between how foraging-like an individual participant was and how well they did in the task (see Methods; no correlation between the foraging index and the probability of choosing the best option [*R* = −0.01, *p* = 0.84] or above-chance reward probability [*R* = 0.03, *p* = 0.64]). With these results, we found no evidence that the use of compare-to-threshold computations was associated with either an advantage or a disadvantage in task performance.

### Foraging-RL better explained very persistent commitments to a single option

In mice, monkeys, humans, and optimized RL models, the distribution of choice run lengths in this task consistently includes two switching regimes: one rapid switching regime related to exploratory trial-and-error sampling and one slow switching regime related to exploitative, repetitive choices^15,24,36^. A selective increase in the duration of the slow switching regime sets humans and other primates apart from rodents in this task^36^, and it is also a behavioral feature that traditional RL models do not naturally capture^15^. Therefore, we next asked whether the foraging-RL might better account for the slow switching regime.

The behavior of both the participants and the two models was all well described as a mixture of 2 regimes (Fig. 4e, f and Supplementary Table 3; see Methods). However, the switching regimes in the foraging-RL model better matched the participants than those in the traditional RL model. Both the foraging-RL and the traditional RL models tended to switch more frequently than humans did during their own fast-switching, exploratory regimes (Fig. 4g; traditional RL = 1.13 trials, 95% CI = [1.08, 1.19]; foraging-RL = 1.16, 95% CI = [1.08, 1.23]; participants = 1.29). This may be due to the fact that neither model accounted for the complexity of exploratory decision-making, which can be information maximizing rather than random^39–42^. Nonetheless, only the foraging-RL was able to replicate the average length and frequency of the participants’ slow-switching, exploitative regimes (Fig. 4h, i; relative frequency of short versus long choice runs: foraging-RL = 84.4%, 95% CI = [82.8%, 86.0%]; traditional RL = 81.4%, 95% CI = [79.8%, 83.0%]; participants = 85.3%; long-run length: traditional RL = 12.15 trials, 95% CI = [11.38, 12.86]; foraging-RL = 15.05, 95% CI = [13.70, 16.42]; participants = 14.99). Participants had more long choice runs than we would expect under traditional RL (1.8% of runs were longer than 10% of the experiment [30 trials], more than the 1.6% predicted by traditional RL, 95% CI = [1.4%, 1.7%]; Fig. 4j), but not foraging-RL (1.8%, 95% CI = [1.7%, 2.0%]). Foraging-RL was also better able to account for the 4/258 participants we had to exclude from all previous analyses because they chose the same option 300 trials in a row, despite passing our initial screens. Because parameters were not identifiable in these participants, they represented held-out data that did not influence the simulations in any way. We found that 300-trial same-choice runs occurred occasionally in foraging-RL (178 of 25,400 or 0.7% of simulated sessions), but were an order of magnitude less common in traditional RL simulations (4 of 25,400 or 0.016% of simulations). While we would expect 4 or more participants to chose the same option 300 trials in 10.9% of experiments under foraging-RL, this outcome has less than a 1 in 1 million chance of occurring under traditional RL (Fig. 4k). In sum, the foraging-RL better accounted for the participants’ persistence, both in those who were included in the model fits and in those that were excluded because of their persistence.

### Foraging-RL better captures the dynamics of exploration and exploitation

To understand why the foraging-RL was better at reproducing the participants’ switching dynamics and persistence, we used latent state models to interrogate the latent dynamics of exploration and exploitation in both the participants and the models (see Methods; refs. ^15,24,40,41^). Briefly, we modeled exploration and exploitation as the latent states underlying sequences of decisions through training hidden Markov models (HMMs) on observed choice sequences (Fig. 4l; refs. ^15,24^), making statistical inferences about the latent dynamical structure of behavior^24,36,40^. The foraging-RL model better matched the participants’ explore/exploit state dynamics than did the traditional RL model. The state transition frequencies estimated with the HMMs were out of sample for the traditional RL model simulations, but within the sample of the foraging-RL model. For example, the participants repeated exploration 90.4% of the time, which was higher than the traditional RL model simulations (87.3%, 95% CI = [86.3%, 88.4%]) but close to the mean of the foraging-RL simulations (90.3%, 95% CI = [89.8%, 90.9%]). Similarly, the participants repeated exploitation 93.9% of the time, which was higher than traditional RL (90.9%, 95% CI = [90.5%, 91.3%]) but close to the mean foraging-RL model (93.7%, 95% CI = [93.4%, 94.1%]). Analyses of the ergodic distributions of the fitted HMMs (see Methods) supported the same conclusions: the participants’ long-term probability of exploration and exploitation (39.2% explore, 60.8% exploit) was out of sample for the traditional RL model (41.6% explore, 95% CI = [40.4%, 42.9%]; 58.4% exploit, 95% CI = [57.1%, 59.6%]), but well within sample for the foraging-RL model (39.3% explore, 95% CI = [38.4%, 40.2%]; 60.7% exploit, 95% CI = [59.9%, 61.6%]). To provide an intuitive summary of these results, we plot the energetic landscape of these dynamics (Fig. 4m; see Methods; refs. ^24,36,40^). Here, we see that exploration and exploitation are less stable states in the traditional RL model than they are in either the participants or the foraging-RL model (Fig. 4m). Together, these results suggest that the foraging-RL model is able to more precisely describe a wider range of participants because it captures the participants’ true dynamics of exploration and exploitation, while the traditional RL model is consistently off target.

### Foraging-RL better predicts behavior across different environments

It remained possible that the results in Experiment 1 were a consequence of the specific environment we chose (i.e., a slow-moving bandit task with two independent option values that was conducted online). We therefore fit the models to 4 additional datasets in which we manipulated (1) the independence of option values (Experiment 2; two options), (2) the volatility of values (Experiment 3, 3-options), (3) the number of options (Experiment 4; 2-, 3-, and 4-options), and (4) the experimental setting (Experiment 5; in person, multiple response modalities). Experiment 2 allowed us to hold richness constant across the task and, along with Experiment 3, to bidirectionally manipulate the uncertainty of the unchosen option(s). Experiments 3 and 4 allowed us to determine how the number of available alternatives influenced model fit. Finally, because Experiments 1–4 were all conducted online, Experiment 5 replicated the original experiment in person, using both oculomotor and touchscreen response modalities.

**Fig. 5:**
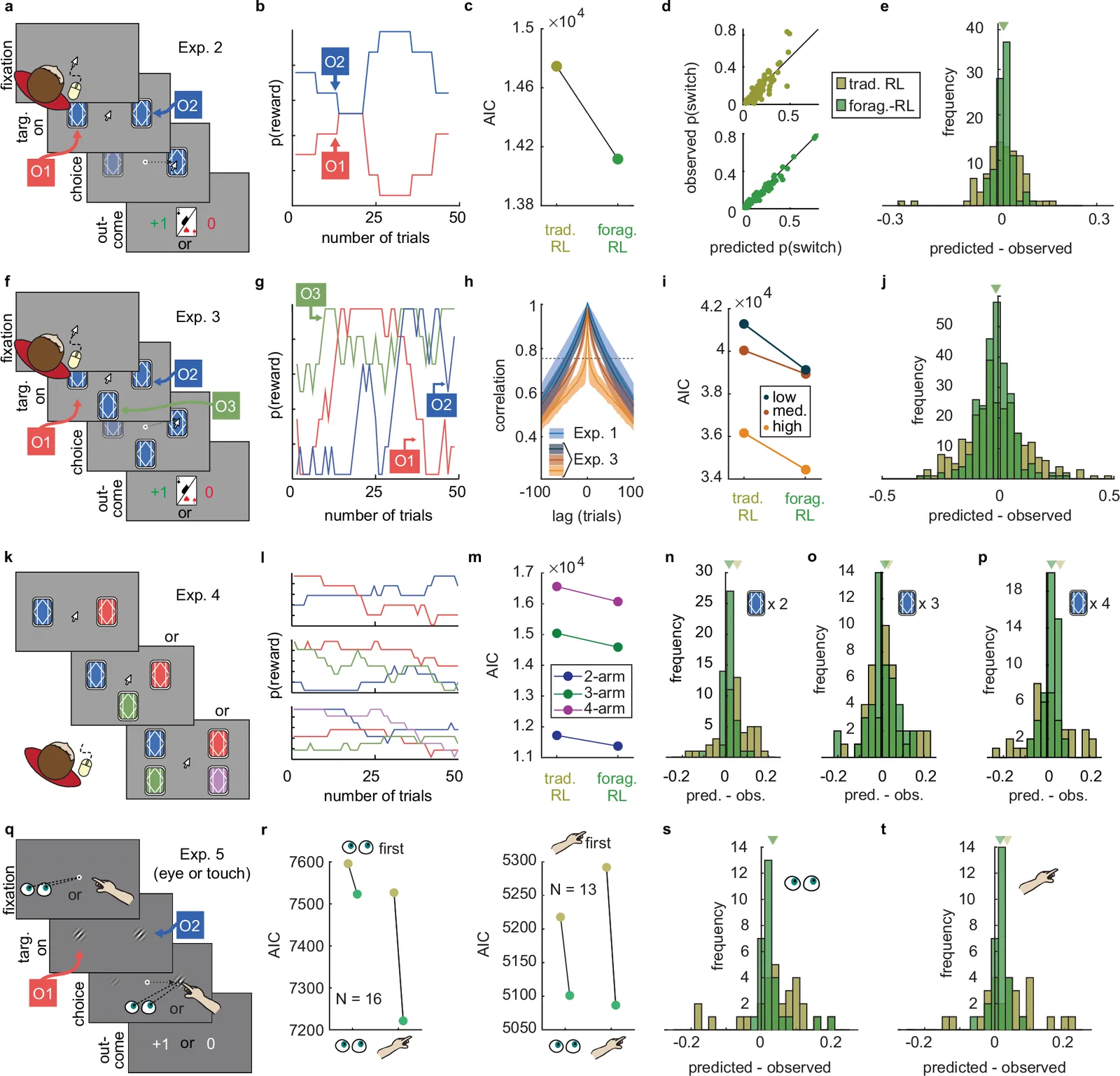
Foraging-RL better describes participants’ behavior across multiple tasks. **a** Experiment 2. Participants (*n* = 95) performed a 2-option bandit task in which option values always summed to 1. **b** Experiment 2 example reward schedule (red trace = option 1; blue trace = option 2). **c** The Akaike information criteria (AIC) of traditional RL and foraging-RL model fits to Experiment 2. **d** Switch probability as a function of predicted probability of switch from the fitted traditional RL model (top) and the foraging-RL model (bottom). Dots = participants. **e** Distribution of the difference in predicted versus observed switch probability for both models. Colors as in (**d**), carets = means. **f** Experiment 3. Participants (*n* = 270) performed a volatile 3-option bandit task where option values changed more rapidly than in Experiments 1 and 2. **g** Experiment 3 example reward schedule. **h** Autocorrelation in reward schedules averaged over Experiment 1 participants (blue), and the 3 volatility bins in Experiment 3 (dark purple, red, orange). Line = mean, shading = STD, dotted line = decay threshold. **i** The AIC of traditional RL and foraging-RL model fits to all data from Experiment 3. **j** Same as **e** for Experiment 3. **k** Experiment 4. Participants (*n* = 150) performed 2-, 3-, or 4-option bandit tasks (50 participants per condition). **l** Experiment 4 example reward schedules for 2- (top), 3- (mid), and 4- (bottom) option conditions. **m** The AIC of traditional RL and foraging-RL model fits to all data from Experiment 4. Same as **e** for 2- (**n**), 3- (**o**), and 4- (**p**) option conditions. **q** Experiment 5. Participants (*n* = 29) performed two versions of a 2-option bandit task. In the touch version, participants physically touched the chosen option on a touchscreen. In the eye version, participants saccaded to and held fixation on an option to select it. **r** (left) The AIC of traditional RL and foraging-RL model fits to data from Experiment 5 for either participants who experienced the eye (left) or touch version of the task first (right). Distribution of the difference in predicted versus observed switch probability for both models for the eye (**s**) and touch (**t**) tasks. caret = mean.

In Experiment 2, participants (95 people, 41 females, 1 other or non-reporting) performed a variation of the bandit task in which the sum of reward probabilities always totaled one for any given trial (Fig. 5a, b). This accomplished three things. First, especially in neuroscience, traditional RL models are often used to analyze behavior from tasks in which option values are tied (e.g., in probabilistic reversal learning tasks^43,44^), so this allowed us to determine if the foraging-RL model also does better in this circumstance. Second, having option values sum to 1 eliminates any variation of richness, which, as we previously showed, influences compare-to-threshold choices but not compare-alternatives (Fig. 2). We therefore reasoned that eliminating this variable might bias people towards compare-alternatives computations. Third and finally, this manipulation reduces the uncertainty of the unchosen option because its value can always be inferred from the chosen option (it was always 1 minus the value of the chosen option). At the group level, the foraging-RL model was again a better fit to human behavior than traditional RL (Fig. 5c, foraging-RL: AIC = 14,116.5, 3 parameters by 94 participants = 282 parameters; log-likelihood = −6776.25, traditional RL: AIC = 14,746, relative AIC weight  < 0.001, 2 × 94 participants = 188 parameters; log-likelihood = −7185.02, 28,200 total trials). The foraging-RL model was also a better fit on an individual basis (foraging-RL: median individual AIC = 131.85, median individual log-likelihood = −62.93; traditional RL: median individual AIC = 139.11, median individual log-likelihood = −67.56; significant paired Wilcoxon signed rank test: *W*(93) = 1658, *p* = 0.030, average difference = −6.697, difference range = [−108.517, 31.213]) (Note that we were unable to fit the model in 1/95 participants because that participant repeatedly selected the same option 300 times ([1.1%]; similar to the proportion that did so in Experiment 1: 4/258 [1.5%])). To understand why foraging-RL was a better fit, we again generated a traditional RL and foraging-RL agent for each participant’s parameters and reward walk (see Methods) and compared each to the participant. In line with Experiment 1, foraging-RL better accounted for the participants’ tendency to switch between options (Fig. 5d, e, foraging-RL: root mean squared error (RMSE) = 0.043 (95% CI = [0.035, 0.051]); traditional RL: RMSE = 0.079, (95% CI = [0.072, 0.086]); Wilcoxon signed rank test *W*(93) = 3624, *p* < 0.001, average difference = 0.004, range = [−0.006, 0.092]). Together, these results suggest that compare-to-threshold continues to be a better fit even when values are matched, and richness does not vary.

In Experiment 3, participants (270 people, 112 females, 2 other or non-reporting) performed a 3-armed version of the bandit task in which we manipulated the volatility of the option values (see Methods, Fig. 5f, g). The volatility manipulation was chosen because the traditional RL model failed to account for the stickiness of participants’ behavior in Experiment 1. Because increasing volatility tends to increase switching behaviors^45,46^, we reasoned that this manipulation might bring participants more in-line with the switching dynamics in the traditional-RL model, giving this model a better chance of capturing participants’ behavior. We increased the volatility of the environment across 3 levels: low, medium, or high levels (Fig. 5h). Regardless of how much we increased volatility, the foraging-RL model remained the better-fitting model at the group level (Fig. 5i, relative traditional RL AIC weights for low volatility < 0.001; medium volatility < 0.001; and high volatility < 0.001; see Supplementary Table 4). The foraging-RL model was also a better fit on an individual basis than traditional RL (all environments (grouped): foraging-RL: median individual AIC = 406.3, median individual log-likelihood = 200.1; traditional RL: median individual AIC = 444.3, median individual log-likelihood = −220.2; Wilcoxon signed rank test *W*(268) = 10,031, *p* < 0.001, average difference = −18.522, difference range = [−232.621, 142.421]). One of 270 participants (<1%) was excluded for choosing the same option for the entirety of the session. We again generated behavior from fitted traditional RL and foraging-RL agents and compared them with the participants. Again, the foraging-RL model better accounted for participants’ overall tendency to switch between options (Fig. 5j, foraging-RL: RMSE = 0.088 (95% CI = [0.083, 0.092]); traditional RL: RMSE = 0.137 (95% CI = [0.134, 0.142]); Wilcoxon signed rank test *W*(268) = 25,301, *p* < 0.001, average difference = 0.011, difference range = [−0.072, 0.305]). Together, these results suggest that foraging-RL better explained human behavior even in high volatility environments that should have been best suited to the kind of frequent switching seen in traditional RL models.

Because 3 targets were used in Experiment 3, we could also leverage this dataset to determine how the value of alternatives influenced choice. In a 3-alternative setting, compare-alternatives computations would be more likely to switch to the best alternative option, whereas compare-to-threshold computations would explore uniformly because alternative values are not tracked. During exploratory switching (Fig. S6a, b; see Methods), a minority of participants had some bias between the two alternative options. However, the majority of these chose the worst alternative option, not the best. By contrast, traditional RL models would predict a large number of individuals with a bias towards the best alternative, not the small number seen in practice (i.e., 2.4% -7.8%, 5–18 people, depending on method; 2.5% expected by chance). However, the fact that 35–46 participants had any bias at all was also not consistent with the assumption that exploration is random and that unchosen option values are forgotten instantaneously in the foraging-RL models. This was especially apparent in switch-backs—decisions that revert to a previously exploited option (compare Fig. S6c, d)—implying that adding some encoding of the last exploited option’s identity could improve the foraging model substantially. Similarly, adding some capacity to preferentially explore options that have not been recently sampled would likely both improve the foraging model and account for the results in Figs. 4g and S6a, b/d, because these options are statistically more likely to be lower value than those that the model has recently chosen, given that the model tends to choose good options (Fig. 3c–f). In short, while a minority of participants exhibited biases between alternative options in the 3-option case, rectifying these observations with the foraging model could be accomplished through adding a working memory component that recalls (1) the last exploited option, and (2) the most uncertain option, without tracking alternative values.

In Experiment 4, participants (150 people, sex and gender not recorded) performed 2-, 3-, or 4-armed versions of the bandit task (Fig. 5k, l). Because 3 alternatives were used in the volatility experiment (Experiment 3), but only 2 alternatives were used in Experiments 1 and 2, it was important to determine if changing the number of options was sufficient to systematically change the goodness of fit of each model. Because the dimensionality of compare-alternatives computations scales with the number of options, it seems likely that the foraging-RL model might offer the greatest efficiency advantage as the number of options grows. Indeed, at the group-level, we saw that the foraging-RL model was a better fit than traditional-RL at the group-level (Fig. 5m, relative traditional RL AIC weights for 2-armed < 0.001; 3-armed < 0.001; and 4-armed < 0.001; see Supplementary Table 5), with the superiority of the foraging-RL model increasing directly with the number of options (2-armed: ∣ΔAIC∣ = 347; 3-armed: ∣ΔAIC∣ = 455; 4-armed: ∣ΔAIC∣ = 492). The foraging-RL model was also a better fit at the individual level (all environments (grouped): foraging-RL: median individual AIC = 263.7, median individual log-likelihood = −128.9; traditional RL: median AIC = 278.5, median log-likelihood = −137.3; Wilcoxon signed rank test *W*(146) = 3973, *p* = 0.005, average difference = −8.802, difference range = [−145.159, 200.153]). We next compared data simulated from each fitted model with the corresponding participant and found that the foraging-RL model again better accounted for participants’ overall tendency to switch between options, although this difference was not statistically significant within the 50 participants in the 3-armed condition alone (Fig. 5n–p, 2-armed: foraging-RL: RMSE = 0.045 (95% CI = [0.036, 0.054]); traditional RL: RMSE = 0.11, 95% CI = [0.095, 0.12]; Wilcoxon signed rank test *W*(49) = 1227, *p* < 0.001, average difference = 0.009, difference range = [−0.001, 0.073]; 3-armed: foraging-RL: RMSE = 0.093 (95% CI = [0.078, 0.1]); traditional RL: RMSE = 0.13, (95% CI = [0.12, 0.14]); Wilcoxon signed rank test *W*(46) = 697, *p* = 0.159, average difference = 0.009, difference range = [−0.037, 0.085]; 4-armed: foraging-RL: RMSE = 0.056 (95% CI = [0.047, 0.067]); traditional RL: RMSE = 0.17, 95% CI = [0.16, 0.19]; Wilcoxon signed rank test *W*(49) = 1055, *p* < 0.001, average difference = 0.026, difference range = [−0.006, 0.271]). In sum, the relative fit of the foraging-RL model increased with the number of arms, perhaps reflecting the theoretical computational advantage that this strategy could offer by only tracking 1 value (versus 2, 3, or 4 values).

Finally, in Experiment 5, we performed an in-person experiment with 29 participants (19 females, 10 males) in which we manipulated the modality of the response within-subjects. This manipulation was meant to address the fact that participants in Experiments 1–4 were recruited via the online platform Amazon MTurk, which limits environmental control and could reduce participant engagement, and to determine if similar results would be obtained regardless of response modality. In the lab, each participant completed two versions of a 2-armed bandit task: one using a touchscreen and another using an eye-tracking device (Fig. 5q). Order was randomized and counterbalanced. Foraging-RL model parameters were correlated across testing blocks, though the threshold parameter *ρ* was the most stable (rho: *r* = 0.79, *p* < 0.001; alpha: *r* = 0.45, *p* = 0.015; beta: *r* = 0.35, *p* = 0.064). These parameter correlations were higher than those observed with a traditional RL model (alpha *r* = 0.42; *p* = 0.022; beta *r* = −0.062, *p* = 0.748). The foraging-RL model again provided a better fit to human behavior than the traditional RL model at the group level, independently of the response modality (touchscreen or eye-tracking) and condition order (Fig. 5r, relative traditional RL AIC weights for all presentation orders and response modalities < 0.001, see Supplementary Table 6). While model comparison results were not significant at the individual level in this small sample, we again found that, the foraging-RL better accounted for individual participants’ tendency to switch between options in both the eye-tracking task, and the touchscreen task (Fig. 5s, t; eye-tracking: foraging-RL model: RMSE = 0.045 (95% CI = [0.036, 0.054]); traditional RL: RMSE = 0.11, (95% CI = [0.095, 0.12]); significant Wilcoxon signed rank test, *W*(28) = 377, *p* < 0.001, average difference = 0.006, difference range = [−0.017, 0.034]; touchscreen: foraging-RL: RMSE = 0.061 (95% CI = [0.053, 0.069]); traditional RL: RMSE = 0.099, (95% CI = [0.091, 0.11]); significant Wilcoxon signed rank test, *W*(28) = 398, *p* < 0.001, average difference = 0.012, difference range = [−0.004, 0.097]). Further, we also found that the foraging-index, a measure of the relative goodness of model fit (see Methods), was correlated across modalities (*r* = 0.63, *p* < 0.001). Overall, these findings show that participants consistently used the compare-to-threshold strategy, regardless of the response modality (touchscreen vs. eye-tracking) and the environmental setting (online vs. in-person).

## Discussion

Although human and animal decisions in sequential decision-making tasks are typically modeled as a compare-alternatives process^7,13,14,35,47–52^, this is an assumption that has not been rigorously tested. Here, in restless, probabilistic *k*-armed bandit tasks, we found that human behavior is, on average, better explained by compare-to-threshold computations. We leveraged this finding to build a class of compare-to-threshold RL models (foraging-RL), which we compared to traditional compare-alternatives RL models^1,4,6,7^. The foraging-RL models were a better fit to participants’ behavior compared to traditional RL models^1,4,6,7^, even when comparing alternatives would have been the more advantageous strategy. Foraging-RL model fits were consistently better across multiple variations of the restless, probabilistic *k*-armed bandit task—including manipulations of volatility, response modality, number of options, uncertainty about the unchosen option, and experimental setting (i.e., both online and in person). Across the environments we tested, the foraging-RL model better matched participants’ tendency to switch options, both on average and on an individual basis—accounting for both people who switched often and people who switched rarely. The foraging-RL model was even able to predict the existence of multiple participants who never switched at all: participants that essentially should not have existed under traditional compare-alternatives RL. This occurred because this model was better able to capture the distinct dynamics of exploration and exploitation in this task.

Although the majority of participants’ choices were better described as a compare-to-threshold process, we did find evidence of compare-alternatives computations in a small minority of participants (1.2, 4.3, 2.2 or 7.8%, depending on the analysis). We cannot rule out the possibility that this proportion would be higher in different tasks or in unexplored variants of this task (e.g., change point tasks, static bandits, bandit problems where reward magnitude drifts rather than probability, or bandit problems without feedback noise). Because the compare-to-threshold strategy is both computationally simpler and less rewarding than the compare-alternative strategy, it seems plausible that different results might be obtained if the stakes were higher and/or the cognitive demands lower. Indeed, when we raised the demands, increasing the number of options and the dimensionality of the task, the advantage of the foraging-RL model also grew—an intriguing parallel to the computational advantage that could come from adopting a strategy that needs to track only 1 value in increasingly high-dimensional environments. Of course, future within-subjects work is needed to determine if this result reflects a change in strategy across individuals or just some improved ability to discriminate the models in higher-dimensional environments. One manipulation that would be especially important in future studies is full outcome feedback (where the outcome of both chosen and unchosen options is explicitly provided, e.g., refs. ^49,51^. Full feedback might encourage compare-alternatives strategies^51^, though it would be important to differentiate true compare-alternatives computations (where option values are tracked independently over time) from a compare-to-threshold strategy that operates on differenced values (i.e., where relative value is calculated at the time of encoding, rather than at selection). Of course, we do not mean to exclude the possibility that humans use a repertoire of strategies, switching between them as needed. For example, in the 3-alternative task, a substantive proportion of participants (16%) did not explore alternative options uniformly, though roughly equal proportions of these were biased towards the best alternative (consistent with using a compare-alternatives strategy for exploration) or towards the worst (consistent with an as-yet-unidentified exploratory strategy). While more work is clearly needed, these results do suggest that compare-to-threshold models should be evaluated even in tasks that are conventionally modeled using compare-alternatives computations.

There are several critical differences between the structure and behavior of the foraging-RL model compared to the traditional RL model. In the traditional RL model, values represent the subjective utility of each primitive action (choosing option 1 through N), and these values are compared in order to make a choice. By contrast, the foraging-RL model does not represent the value of primitive actions, but instead represents a single focal temporally extended action (i.e., of exploiting the current option). In this way, the foraging-RL model actually resonates with recent work on hierarchical reinforcement learning that suggests that people make choices at the level of policies rather than primitive actions^53–59^. Higher-level policies, macro actions, and options have been studied for over two decades in the RL literature^53^. They offer candidate mechanisms for achieving better generalization and transfer of knowledge in artificial agents and could improve the computational efficiency of sequential decision-making^53,56^. The fact that we can use a compressed representational structure to compare a single value to a threshold could also make the foraging-RL model better able to scale outside of laboratory tasks, where the number of options available is generally much larger than the small sets typically used in lab tasks. Where the representations and computations in traditional RL models become costly quickly in high-dimensional spaces, our foraging-RL model does not scale in the same way. Further, we noted here that compare-alternatives computations also predict that people should explore the most when discriminability is low and values are most confusable: exactly when the marginal utility of choosing another option is actually the worst (Fig. 2a). Considering that exploratory choices are both costly and time-consuming^15,24,25,41,60^, compare-to-threshold choices offer the additional advantage of exploring only when they offer the biggest potential return—when a previously exploited option begins to disappoint and the search for an alternative is truly warranted. In short, it seems likely that humans favor compare-to-threshold computations because this approach is more computationally efficient, better aligned with the brain’s biological constraints, and better suited to the scale and statistics of natural environments.

The foraging-RL model we introduce here draws much of its inspiration and some of its limitations from the foraging literature. The model assumes the existence of only two macro actions—explore and exploit—and defines a specific low-level policy and termination for each of them. Based on the foraging literature, we assumed that switching from exploit to explore causes instant forgetting of exploited values (i.e., the patch is left behind), that explore actions select targets randomly (i.e., that we travel until we encounter some new opportunity at random). On one hand, drawing these assumptions from the ethological literature is a powerful way to create new computational models and constrain the space of useful macro policies. On the other hand, each of these assumptions is also an opportunity for future research that could improve on the simple foraging-RL model presented here. For example, the foraging-RL model failed to account for the rapid speed of switching during the exploratory regime (Fig. 4g). This suggests that refining the exploratory policy in this model could substantially improve both model fit and agent performance. One promising approach would be to incorporate directed forms of exploration (e.g., self-avoiding search^41^) or intrinsic motivations (e.g., entropy maximizing policies^61^). Policies that would incorporate the capacity to track the identity of previously exploited options or the length of time since an option has been selected seem especially promising, given the data we observed here. Developing more sophisticated exploratory strategies is likely necessary for translating the simple foraging-RL model we present here into something that could be useful in AI. In that sense, our work could represent a bridge between observations in animal behavior and major challenges in AI, including the problem of identifying useful macro action spaces. In short, developing new, biologically-inspired models of human decision-making could have broader impacts: not only for understanding the human mind, but also for generating powerful new tools in AI.

One place in which our foraging model deviates from the ethological literature is in the threshold parameter. In foraging theory’s patch leaving models^16,17^, the threshold parameter is determined by the background rate of reward in the environment. Previous compare-to-threshold learning models similarly implemented a threshold that dynamically updates itself to estimate this background rate of reward^21^. By contrast, we decided to model the threshold as an individual difference parameter that simply controls the rate of exploration. We did this because the link between the threshold and the background rate of reward is normative, rather than empirical. While theory shows that average rewards can be used to identify the optimal time points at which a forager should leave a decaying patch, real-world foragers tend to systematically under- or over-harvest with respect to average-reward thresholds^18,21,62–66^. Modeling the threshold as a fixed individual difference parameter resonates with these results, but also with prior observations that people have long-term and stable preferences in the amount they explore (e.g., ref. ^67^). We know that the frequency of exploration changes with individual differences in personality characteristics and life circumstances that are not related to the rate of reward in any given task^24,49,50,68–72^, and we reported here that the threshold was far and away the most stable parameter across repetitions of the task in Experiment 5. Ultimately, it is probably best to conceptualize the foraging-RL threshold as a parameter that integrates multiple sources of information, such as previous reward history, inherent beliefs about environments, personality characteristics, and/or unmeasured variability in mood or arousal. Future work is needed to understand how these factors shape the threshold for exploration and over what time scales it may change.

The observation that compare-to-threshold foraging-RL models are a better fit to human behavior may superficially appear to contradict the evidence for RL-like computations in the neuroscience literature. However, the reward prediction errors and value functions for which we have strong neural evidence also exist in the foraging-RL model. Both models use reward prediction errors to learn their respective value functions. Further, although neural activity often correlates with action value^73–79^, and this signal is thought to play a role in specifying primitive actions, primitive action-values are likely to be highly collinear with the macro action-values calculated by the foraging-RL model. Because no direct comparison between these two value signals has been performed, evidence for the former cannot be considered evidence against the latter. Further, it is important to be cautious in interpreting correlations between value and neural activity because (1) time series data are prone to spurious correlations, (2) putative neural correlates of value could be caused by other mechanisms, like mnemnonic errors^80^, and (3) neural activity can have a complex, nonlinear relationship with value^81^, which significantly complicates the problem of determining whether a neural signal reflects the value of a primitive action or the value of exploiting that action. That said, some neural observations—such as nonlinearities in neural activity around shifts between exploration and exploitation^15,25,82–84^—are already difficult to reconcile with traditional RL models. By contrast, the abrupt value dynamics of the foraging-RL at state switches could begin to explain some of these observations. Ultimately, neuroscience research will hold the key to determining whether the computations humans perform are truly more consistent with compare-alternative or compare-to-threshold accounts.

In sum, this paper reinforces and extends a body of recent observations that humans, like many other animals, use foraging-like compare-to-threshold computations to make sequential decisions^19–21^. However, where previous studies of foraging computations in psychology and neuroscience used foraging-specific tasks—tasks that are explicitly designed to encourage foraging strategies or computations^18–23,85,86^—the present study did the opposite. Through developing a foraging-inspired RL model that is capable of navigating dynamic, unpredictable environments, we determined that humans will use foraging-like compare-to-threshold computations even in tasks that are the traditional testbeds for compare-alternatives RL models.

## Methods

### Data collection

#### Ethics approval

All experimental procedures were in line with the standards set by the Declaration of Helsinki and were approved by the relevant ethical review boards. Experiment 1 was approved by the Comité d’Éthique de la Recherche en Sciences et Santé (CERSES) of the University of Montreal [Project #2021-1090]. Experiment 2 was approved by the CERSES of the University of Montreal [Project #2021-1090]. Experiment 3 was approved by the Institutional Review Board of the University of Minnesota [STUDY00008486]. Experiment 4 was approved by Princeton University Institutional Review Board [IRB #7873]. Experiment 5 was approved by the CERSES of the University of Montreal [Project #2021-1090].

#### Sample size

Sample sizes were chosen to be sufficiently powered to detect expected effect sizes of interest (1−*β* = 0.8).

#### Sex and gender

Sex and gender were considered to the extent that each study had a balanced sample of self-identified male and female participants; no sex and gender-based analyses were reported here because these are topics for future research.

### Participants

#### Online experiments (1-4)

Participants in Experiments 1–4 were recruited via the online platform, Amazon Mechanical (MTurk). To avoid bots and improve data quality, participants were only accepted when they had a minimum of 5000 approved human intelligence tasks (HIT) and a minimum percentage of 98% in proportion of completed tasks that are approved by requesters. Geographical restrictions were set for US participants only. To help ensure that participants understood the task, there was an initial 25-trial block with fixed but randomly assigned reward probabilities (20, 30, and 70%). To continue to the main task, participants were required to (1) switch between options at least twice, and (2) get a reward more than 42% of the time during the practice block. The criteria and number of trials were chosen to ensure that people choosing at random would rarely pass through to the main task. Participants were not allowed to repeat the experiment. All participants who successfully submitted the HIT, either the practice block or the full experiment, were paid a base rate of $0.50, plus a bonus of $3.61 mean ± $0.97 SD based on their performance (participants were given a $0.02 compensation for each trial that ended with a reward). All participants completed 300 trials in each experiment. Participants provided informed consent following a full explanation of the experimental procedure that reminded them of their right to withdraw at any time during the study.

For Experiment 1 (2-option bandit task), a total of 258 participants (120 females, 137 males, 1 preferred not to say) completed the task. For Experiment 2 (2-option bandit task with linked rewards), a total of 95 participants (41 females, 51 males) completed the task. For Experiment 3 (3-option bandit task with variable volatility), a total of 270 participants (112 females, 156 males, 2 prefer not to say) completed the task. For Experiment 4 (multi-option bandit task with variable k), a total of 150 participants (sex and gender were not collected) completed the task. Some analyses of Experiment 1, 4, and 5 are included in a manuscript comparing switching behavior between mice, monkeys, and humans^36^, but all analyses presented here are new.

Experiment 1: 2-option restless bandit task: in Experiment 1, participants were required to repeatedly choose one of two options. Each option was associated with a probability of reward, which changed across trials and independently across options (step size = 0.1, hazard rate = 0.1). Each participant’s reward schedule was randomly generated to avoid environmental biases.

Experiment 2: linked 2-option bandit task: to understand how fixing richness and decreasing uncertainty in unchosen option value influences behavior, participants performed a modified version of the two-armed restless bandit task in which option values were linked. One option was set according to the same statistics used in Experiment 1, while the value of the other option was set to 1 minus this value. Because the values always summed to 1, there was no variation in the richness of the environment across trials.

Experiment 3: volatile 3-option bandit task: to explore how increased uncertainty influences behavior, participants performed a 3-option restless bandit task under multiple different levels of volatility in the environment. All volatility levels were higher than those used in Experiment 1. Specifically, both hazard rates and step sizes were variable (hazard rates were between 0.1 and 1; step sizes ranged from 0.050 to 0.667). Participants were classified into one of 3 volatility bins based on terciles of the decay of the autocorrelations in the reward walks (trial at which the autocorrelation decayed to 0.75 of its initial value; Fig. 5h). We also added an additional option in this task, which has no significant effect on the probabilities of switching or exploring^36^, but did allow us to also analyze any dependence of alternative option value on choices (Fig. S6).

Experiment 4: multi-option bandit task: to understand how the number of alternatives options influenced behavior, participants performed either 2-, 3- or 4- option restless bandit task (50 participants in each group). Experiment 4 also used a slightly higher hazard rate than Experiment 1 (0.2) to encourage exploration.

#### In-person experiment (5)

Experiment 5: in-person 2-option bandit task: we conducted an in-lab experiment using a 2-option restless bandit task to achieve two goals: (1) determine if compare-to-threshold computations are still preferred in an in-person setting (as opposed to online platforms like Amazon MTurk) and (2) test if different response modalities (eye tracking vs. touchscreen) affected the choice of algorithm. The experiment consisted of 20–25 trial practice blocks to familiarize with the task, followed by 2 testing blocks (1 for each modality: eye movements and touch screen presses). While online experiments used playing cards as stimuli, in-person experiments cued option locations with small equiluminant gabors and included small (<1 degree) central fixations that had to be looked at (in eye modality) or touched (in touch modality) to initiate a trial (after ^15^). For eye modality responses, monocular eye data were sampled at 2000 Hz with an Eyelink 1000+ infrared eye tracker. Saccades were identified in real-time using custom Matlab routines. Touch responses were recorded via an infrared touch frame (Chengying CYS-27) placed on top of the monitor and processed via standard Paychtoolbox-3 routines. The modality assigned to the first block was randomized to counterbalance order effects. Practice trials were excluded from all analyses. Data was collected between 9 AM and 5 PM EST. A total of 29 participants (19 females, 10 males) completed the task. Participants completed an average of 483.28 ± 43.01 (SD) trials in the eye-tracking version, and an average of 499.48 ± 1.70 (SD) in the touchscreen version. All participants of Experiment 5 provided informed consent.

### Data analysis

#### General analysis methods

Data was analyzed with custom software written in MATLAB and Python. Statistical tests were two-sided unless otherwise specified. Significance was compared against the standard alpha = 0.05 threshold. Correlations are Pearson correlation unless otherwise indicated. A small number of participants (Experiment 1: 4/258 participants, 1.6%; Experiment 2: 1/95, 0.95%; Experiment 3: 1/270, 0.37%, 0.95%; Experiment 4: 3/150, 2%) were excluded from model-based analyses because they chose only one option in the main task, which made the model parameters unidentifiable. These participants were included in other analyses wherever it was possible to do so.

#### Choice predictive generalized linear model (GLM)

To determine whether the value of previously unchosen options influenced choice, we used logistic regression to predict the probability of repeating choice on a trial-by-trial basis for each participant. We defined the log odds of *p*(repeat_*t*_) as

1 $$\log \left(\frac{p({{{{\rm{repeat}}}}}_{t})}{1-p({{{{\rm{repeat}}}}}_{t})}\right)={\beta }_{0}+{\beta }_{1}V({{{{\rm{chosen}}}}}_{t-1})+{\beta }_{2}V({{{{\rm{unchosen}}}}}_{t-1})\,,$$

where *V*( ⋅ ) is the objective value of the option that was chosen or unchosen at time *t* − 1. In this formulation, decisions that depend on comparing values would have positive beta weights for *V*(chosen_*t*−1_) and negative beta weights for *V*(unchosen_*t*−1_), indicating that the probability of choice is proportional to *V*(chosen_*t*−1_) − *V*(unchosen_*t*−1_). Conversely, decisions that depend on comparing to a threshold would only have positive beta weights for *V*(chosen_*t*−1_), because only the focal option value is tracked.

Objective values were used in Fig. 1f, g because these offered the fairest comparison between views. This is because estimating subjective values requires making assumptions about whether the participants are using compare-alternatives or compare-to-threshold computations. That said, it is possible to approximately estimate subjective values by making sensible assumptions about how value could be tracked without fitting any parameters, and repeat the same analyses that we did with objective values.

First, we considered the parameter-free ideal Bayesian learner that is used in static bandit tasks (static bandit Bayesian update; Fig. S1). Assuming participants hold ideal beliefs about the probability of reward of each arm (subjective value from now on) via a Beta(*a*, *b*) distribution with parameters *a*, *b*, following a choice to arm *j* and a reward *r* at time *t*, the parameters of arm *j* are updated according to

2 $${a}_{j,t+1}={a}_{j,t}+r,\quad {b}_{j,t+1}={b}_{t}+(1-r)\,,$$

whereas the unchosen arms do not get updated. These equations directly translate to an updated subjective value *v*_*i*,*t*+1_ = *a*_*j*,*t*+1_/(*a*_*j*,*t*+1_ + *b*_*j*,*t*+1_) and an updated concentration *κ*_*j*,*t*+1_ = *a*_*j*,*t*+1_ + *b*_*j*,*t*+1_. This update gives weight to all rewards received and ignores the drift, which does not hold optimally in restless arm bandits like our task. Subjective values estimated with this model were less predictive of choice than objective rewards (likely because the model assumes perfect integration over history, which is better suited to a static, rather than dynamic environment) and the results were unchanged (Supplementary Fig. S1a, b, only chosen value consistently predicted choice and very few participants appeared to compare values).

Second, we considered an alternative ideal Bayesian learner for whom subjective values decayed optimally (diffusing bandit Bayesian update; Fig. S1), but this again did not change the major result (Fig. S1c, d, only chosen value consistently impacted choice and very few participants appeared to compare values). Briefly, we assume the learner maintains the same type of belief (Beta distribution for each arm) and updates it knowing that the objective values drift over time. To do this, we assume the learner has knowledge of the specific parameters of the random walks (probability of an upwards or downwards step *p* and step size Δ), and use a continuous approach to the diffusion of the probability density, by integrating a diffusion equation (Fokker-Planck without drift). Due to the boundary conditions, this integration naturally obtains that the unchosen subjective values should regress to the prior mean (1/2) with maximum uncertainty, with a decay towards the mean given by the leading order coefficient of the Fourier series that solves the diffusion equation. That is, after each trial *t* the subjective value *v*_*i*,*t*_ of arm *i* should decay following:

3 $${v}_{i,t+1}\approx \frac{1}{2}+\exp \left(-\frac{{\pi }^{2}}{2}p{\Delta }^{2}\right)\left({v}_{i,t}-\frac{1}{2}\right)\,.$$

The variance of the Beta distribution is also updated: Var_*i*,*t*+1_ ≈ Var_*i*,*t*_ + *p*Δ^2^, from which the concentration *κ*_*i*,*t*+1_ of each arm can be updated, where the approximation is coming from assuming the drift is independent of the actual value of the subjective value, which does not hold only at the boundaries. Before each trial, we apply this decay to all the arms. Then, following a choice and a reward, we apply the standard Bayesian Beta update (Eq. (2)) to the chosen arm *j* to obtain a new subjective value *v*_*j*,*t*_ and concentration *κ*_*j*,*t*_.

Because models were fit on a trial-by-trial basis, *p*(repeat) was a binary vector labeled as 1 whenever the choice on trial *t* was the same as the choice on trial *t* − 1. To estimate the relative contribution of each predictor, we also fit two reduced models: one containing only *V*(chosen_*t*−1_) and another containing only *V*(unchosen_*t*−1_). In Fig. 1f, g, the *p* values for the coefficients of interest (*V*(chosen_*t*−1_) and *V*(unchosen_*t*−1_)) obtained from the full model were adjusted using a Holm-Bonferroni correction within each predictor. Model comparison was evaluated with the Bayesian Information Criterion because we found that the Akaike Information Criterion had an insufficient penalty for the number of parameters (i.e., the full model was generally preferred even when option values were completely randomized). Participants who never switched were excluded from this analysis because model parameters were not identifiable.

#### Strategy fingerprints

Compare-alternatives and compare-to-threshold decision-making should, in theory, depend on different aspects of the environment. To quantify these predicted dependencies, we characterized the fingerprints of each strategy as a function of two features of the environment: richness (the sum of the values) and discriminability (the range of the values, normalized to the sum). In order to make predictions for each strategy, we first separated the 258 reward walks experienced by the participants into 100-trial bins (3 bins per session, 774 bins). We then calculated the richness and discriminability within each bin (Fig. 2c, bottom) and the environmental predicted p(switch) for each bin, given each of the two strategies (Fig. 2d, e). Because exploration should occur most often when option values are close together in the compare-alternatives strategy, the environment-predicted p(switch) under this strategy was calculated as the probability that the difference in option values was below some Δ reward for various Δ rewards (4 examples Δ rewards in Fig. 2d). Because exploration should only occur when the focal option is below threshold in the compare-to-threshold strategy, the environment-predicted p(switch) under this strategy was the probability that any randomly selected focal option value would be below some threshold for various thresholds (4 examples thresholds in Fig. 2e).

To quantify how similar participants’ switching patterns were to the compare-alternatives and compare-to-threshold fingerprints, we estimated the shape of the environmental predicted p(switch) at each parameter value for each strategy (i.e., threshold or Δ reward). We did this via fitting generalized linear models (GLMs) to the environment-predicted p(switch) from both richness and discriminability. Two models were fit, a linear (2-parameter) GLM and a quadratic (3-parameter) GLM, for each combination of parameter, strategy, and independent variable. We then extracted 3 key features from each pair of fitted models: (1) the relative deviance of the linear and quadratic models (a measure of the extent of curvature as a function of either richness or discriminability), (2) the quadratic term of quadratic model (a measure of the sign and magnitude of curvature in the relationship), and (3) the linear term of the linear model (a measure of the overall slope of the relationship). This approach allowed us to precisely quantify how switch probabilities were predicted to change as a function of both richness and discriminability under each hypothesized strategy. Distributions of the features for every possible threshold and Δ reward are illustrated in Supplementary Fig. S2.

To consider the fingerprints holistically, rather than as individual features, we then took the 6-feature dimensions described above, added the overall probability of switching to account for DC offsets in switch probabilities as a 7th feature, and then calculated the distance from the human fingerprint to the predicted fingerprint for every possible parameter value for each of the two strategies (Fig. 2h). PCA was performed only to illustrate the approach in Fig. 2g, where distances were calculated in the full space. In this plot, bounding boxes are drawn around all environment-predicted fingerprints under each strategy to illustrate the range of fingerprints that each strategy was capable of generating. Similar results were found regardless of whether GLMs were fit with binomial or identity link functions. The former appears in Fig. 2g, the latter is shown in Fig. S2a–c).

#### Decision-making models

To determine whether participants were using foraging-like compare-to-threshold computations or compare-alternatives-like computations, we fit a series of 18 cognitive models (8 traditional RL, 7 foraging-RL, and 3 dynamic belief models). All models were initialized with 20 random seeds and fit via maximum likelihood (minimizing the negative of the log likelihood; fminsearch, MATLAB, scipy.optimize.minimize, Python). The Akaike Information Criteria was used to compare models with differing numbers of parameters, and Akaike weights were calculated to estimate the relative ability of each model to minimize information loss. Here, we will first describe the standard formulations of both traditional RL and foraging-RL models. The following section will then explain the various extensions of each model.

The majority of models, traditional RL and foraging-RL alike, had a classic delta-rule value updating process at their core,

4 $${v}_{t+1}={v}_{t}+\alpha ({r}_{t}-{v}_{t})\,.$$

That is, values (*v*_*t*_ + 1) were updated at each time step *t* according to the difference between the observed reward (*r*_*t*_) and the previous value (*v*_*t*_; *v*_0_). The magnitude of the update is scaled by a learning rate (*α*; constrained as 0 < *α* < 1 in all models). The delta-rule update dynamically calculates value as an exponentially decaying recency-weighted average of reward^1^. This is a canonical computation, with widespread neural and behavioral evidence supporting its existence in humans and other animals^87–92^. Though both models have the delta-rule computation at their core, they differ in what the value represents: the value of primitive choice options (in traditional RL) or the value of exploiting a particular option (in foraging-RL).

##### Traditional RL models

In traditional RL models, each primitive action *i* has its own value that is updated (or not) at each time step^6^. One common approach, which we used in our standard version of traditional RL models here, is to update only the value of chosen options^1^,

5 $${v}_{i,t+1}=\left\{\begin{array}{ll}{v}_{i,t}+\alpha ({r}_{t}-{v}_{i,t}),\quad &{{{\rm{if}}}}\ i\ {{{\rm{is}}} \, {{\rm{chosen}}}}\\ {v}_{i,t},\hfill \quad &{{{\rm{otherwise}}}}\hfill\end{array}\right.\,,$$

with *v*_*i*,0_=0 for all actions *i* and models in this class.

A softmax function then compares and transforms values into choice probabilities with the usual Boltzmann exploration strategy. This meant that the probability of choosing option *i* at time *t* in our traditional RL models was

6 $$p({{{{\rm{choice}}}}}_{t}=i)=\frac{{\exp }^{\beta {v}_{i,t}}}{{\sum }_{j=1}^{N}{\exp }^{\beta {v}_{j,t}}}\,,$$

where *β* is an inverse temperature parameter that controls the noise in the value comparison and is constrained as [high noise] 0 < *β* < 100 [low noise] in all models.

Because Experiment 1 only had *N* = 2 options, we can expand the sum and rearrange this equation,

7 $$p({{{{\rm{choice}}}}}_{t}=i)=\frac{1}{1+{e}^{-\beta ({v}_{i,t}-{v}_{j,t})}}\,,$$

which may clarify the point that this function directly compares the value of the two options in order to decide which to choose.

##### Foraging-RL models

In the foraging-RL models, values do not represent the value of a single focal option. Instead, the only value tracked in the model is the value of staying with or “exploiting” the option that was just chosen. This means that the update equation is precisely Eq. (4), with no subscripts because the value is not assigned to any specific primitive action.

One useful way to think about the foraging-RL model is to characterize it as a hierarchical action algorithm: there are higher-level, temporally-extended actions that determine the probability of allocating choices at each trial to each target. We can use the options framework^53^ to define this hierarchical model. In this framework, an option *o* consist of three components: a policy *π*, a termination condition ${{{\mathcal{T}}}}$,and an initiation set ${{{\mathcal{I}}}}$,

8 $$o=\left\{\begin{array}{l}\pi :{{{\mathcal{S}}}}\times {{{\mathcal{A}}}}\to [0,1]\,,\quad \\ {{{\mathcal{T}}}}:{{{{\mathcal{S}}}}}^{+}\to [0,1]\,,\quad \hfill\\ {{{\mathcal{I}}}}\subseteq {{{\mathcal{S}}}}\,,\quad \hfill\\ \quad \end{array}\right.$$

where ${{{\mathcal{S}}}}$ is the state space, and ${{{\mathcal{A}}}}$ is the primitive action space. If an option $o=\langle {{{\mathcal{I}}}},\pi,{{{\mathcal{T}}}}\rangle$ is taken, then actions are selected according to *π* until the option terminates stochastically according to ${{{\mathcal{T}}}}$. This framework can be extended to semi-Markov options, where a history Ω over states, actions and rewards can be taken as input to the low level policy *π*. This is important because the foraging-RL model makes use of this history.

In the foraging-RL model, the primitive action space ${{{\mathcal{A}}}}$ is the set of targets that can be chosen, the state space is a trivial one-state space (so initiation sets are trivial), and we define the option space as

9 $${{{\mathcal{O}}}}=\{{{{\rm{explore}}}},{{{\rm{exploit}}}}\}\,,$$

with each option defined the following way:

1. The exploit option has a lower level policy *π*_exploit_ that executes always the same action (given by the previous action from the history), and terminates with probability
  10 $${{{{\mathcal{T}}}}}_{{{{\rm{exploit}}}}}=1-p({{{\rm{exploit}}}}),({{{\rm{given}}}}\,{{{\rm{in}}}}\,{{{\rm{Eq}}}}.12)\,,$$
2. The explore option has a lower level policy ${\pi }_{{{{\rm{explore}}}}}$ that selects targets uniformly randomly (excluding the previous action from the history), and terminates with probability
  11 $${{{{\mathcal{T}}}}}_{{{{\rm{explore}}}}}=p({{{\rm{exploit}}}}).$$

We define the termination condition for exploitation with a softmax rule, in a similar fashion to how the traditional RL model randomly selects other targets, in the following way:

12 $$p({{{\rm{exploit}}}})=\frac{1}{1+{\exp }^{-\beta (v-\rho )}}\,,$$

where *v* is the value of exploitation, which gets updated using Eq. (4), *v*_0_ = 1 for all foraging-RL models and *β* is the inverse temperature parameter constrained as 0 < *β* < 100, and the threshold *ρ*, constrained as 0 < *ρ* < 2, could be thought of as a threshold for exploration, which, by analogy to foraging theory^16,17^, could reflect expectations about the background rate of reward or value of alternative options in the environment.

This way of interrupting or terminating options is the usual choice in the context of hierarchical actions and options^53,93^, where we decide to interrupt the execution of a temporally-extended action. However, where previous models might decide to terminate via comparing the value of that option against alternatives, we compare it to a fixed threshold here. With this space of options, we can now build a policy over options to define the compare-to-threshold algorithm: whenever an option terminates, the other starts.

It is important to note that the foraging-RL algorithm only tracks one value, the value of the exploited option. Whenever the model switches to a new primitive action (explores), the value of exploiting that action is unknown, so the value of exploitation is reset to the threshold. In this way, the value of exploitation always represents the value of terminating a sequence of choices to a particular option, even if the model has only just begun choosing that primitive action.

#### Extensions to decision-making models

In addition to the standard formulations of the traditional RL and foraging-RL models introduced above, we considered a variety of extensions that are commonly used to improve the fit of RL models and developed versions of the foraging-RL model that incorporated each extension.

##### Asymmetrical

First, we considered asymmetrical learning, meaning that we added a parameter that allowed the models to learn at different rates from rewards and omissions^28–30^. Adding asymmetry often improves the fit of RL models^30,94^ and it improved the fit of both models here. In both the traditional RL asymmetry and foraging-RL asymmetry models, the delta-rule update was rewritten

13 $${v}_{t+1}=\left\{\begin{array}{ll}{v}_{t}+{\alpha }_{W}(1-{v}_{t}),\quad &{{{\rm{if}}}} \, {r}_{t}=1\\ {v}_{t}+{\alpha }_{L}(0-{v}_{t}),\quad &{{{\rm{if}}}} \, {r}_{t}=0\end{array}\right.\,,$$

where *v* was the value of the chosen option in the traditional RL model or the value of exploitation in foraging-RL and both learning rates were constrained as 0 < *α*_*W*_ < 1, 0 < *α*_*W*_ < 1 in both models.

##### Decay

Next, we considered decay, meaning a way for the values of options that have not been sampled to diminish over time. This approach emerged from observations in neuroscience^31–33^ and is widely used in the reinforcement learning literature^95–98^, where it has been interpreted as indicative of limited memory resources. It consistently improves traditional RL model fits^96,99–101^ and improves the fit of both models here. In the decay version of the traditional RL model, the value of unchosen options does not carry forward unchanged in time, but instead decays in proportion to *δ* (constrained as 0 < *δ* < 1),

14 $${v}_{i,t+1}=\left\{\begin{array}{ll}{v}_{i,t}+\alpha ({r}_{t}-{v}_{i,t}),\quad &{{{\rm{if}}}}\ i \, {{{\rm{is}}}} \, {{{\rm{chosen}}}}\\ \delta {v}_{i,t},\hfill \quad &{{{\rm{otherwise}}}}\hfill\end{array}\right.\,.$$

Although the foraging-RL model has no unchosen values to decay, it does have a threshold parameter, *ρ*, which was previously modeled as static, but now allowed to change over time

15 $$p({{{{\rm{exploit}}}}}_{t})=\frac{1}{1+{e}^{-\beta ({v}_{t}-{\rho }_{t})}}\,.$$

Specifically, *ρ* could now decay with each successive choice to the same option through updating it in a state-dependent way as a function of some decay parameter *δ* (constrained as 0 < *δ* < 1),

16 $${\rho }_{t+1}=\left\{\begin{array}{ll}\delta {\rho }_{t},\quad &{{{\rm{if}}}} \, {{{\rm{exploit}}}}\\ {\rho }_{0},\quad &{{{\rm{if}}}} \, {{{\rm{explore}}}}\end{array}\right.\,,$$

where *ρ*_0_ is the value at which *ρ* is initialized at time 0 (randomly sampled between 0 and 2) or *ρ*_0_ = *ρ* at the onset of exploration.

##### Choice history

We also considered 3 variations of choice-history dependence, meaning an outcome-independent tendency to repeat past behavior, also borne out of observing that primate behavior was better explained with a choice ’kernel’^34,35,102^. Adding dependence on previous choices tends to improve the fit of traditional RL models^4,24,34,35^, likely because of the strong tendency towards hysteresis in the choices of humans and other animals. Adding history dependence also improved the fit of both models here. We began by simply adding a choice kernel (*k*) for each option *i* in the traditional RL model. This is updated in parallel with each action value as

17 $${k}_{i,t+1}=\left\{\begin{array}{ll}{k}_{i,t}+{\alpha }_{c}(1-{k}_{i,t}),\hfill \quad &{{{\rm{if}}}} \, {{{\rm{choice}}}}=i\\ {k}_{i,t}+{\alpha }_{c}(0-{k}_{i,t}),\hfill \quad &{{{\rm{otherwise}}}} \hfill \end{array}\right.\,,$$

where *α*_*c*_ is a choice kernel learning rate and is constrained as 0 < *α*_*c*_ < 1 for both models. The choice kernel value is then combined with value in the softmax decision rule^34^,

18 $$p({{{\rm{choice}}}}=i)=\frac{{\exp }^{\beta ({v}_{i}+{k}_{i})}}{\mathop{\sum }_{j=1}^{N}{\exp }^{\beta ({v}_{j}+{k}_{j})}}\,.$$

Thus far, we have added only 1 choice-history dependent parameter to this traditional-RL model, the *α*_*c*_ parameter, and this model is the history kernel 1 traditional-RL model referred to in the text (RL choice+1, in Fig. 3j, k). However, it is possible to give choice history its own inverse temperature parameter *β*_*c*_^35^, constrained as 0 < *β*_*c*_ < 100 for both models. This is the history kernel 2 traditional-RL model (RL choice+2, in Fig. 3j, k),

19 $$p({{{\rm{choice}}}}=i)=\frac{{\exp }^{\beta {v}_{i}+{\beta }_{c}{k}_{i}}}{\mathop{\sum }_{j=1}^{N}{\exp }^{\beta {v}_{j}+{\beta }_{c}{k}_{j}}}\,.$$

Although there was no natural way to add a choice history kernel to the foraging-RL model, we could use a nearly identical approach to add a state history kernel. This approach adds hysteresis at the level of exploitation itself, rather than at the level of the primitive actions. We introduced a state history kernel that was updated alongside the value of exploitation as

20 $${k}_{t+1}=\left\{\begin{array}{ll}{k}_{t}+{\alpha }_{c}(1-{k}_{i,t}),\quad &{{{\rm{if}}}} \, {{{\rm{exploit}}}}_{t}\\ 0,\hfill \quad &{{{\rm{otherwise}}}}\end{array}\right.\,,$$

meaning that the state history kernel was re-set to 0 when the animal explored. In the history-kernel 1 foraging-RL model (foraging choice + 1, in Fig. 3j, k), the state-history kernel was then added directly to the decision rule,

21 $$p({{{{\rm{exploit}}}}}_{t})=\frac{1}{1+{\exp }^{-\beta ({v}_{t}-\rho+{k}_{t})}}\,.$$

In the history-kernel 2 foraging-RL model (foraging choice + 2, in Fig. 3j, k), the kernel was inserted, but scaled with its own inverse noise parameter, *β*_*k*_, as

22 $$p({{{{\rm{exploit}}}}}_{t})=\frac{1}{1+{\exp }^{-\beta ({v}_{t}-\rho )-{\beta }_{k}{k}_{t}}}\,.$$

Because there were significant computational differences between how choice-history dependence functioned in the foraging-RL model, compared to traditional RL, we also considered a 1-parameter repetition bonus extension of a traditional RL model, in which some probability of repeating the immediate last choice was added into the softmax decision rule as

23 $$p({{{{\rm{choice}}}}}_{t}=i)=\frac{{\exp }^{\beta ((1-b){v}_{i,t}+b{c}_{i,t})}}{\mathop{\sum }_{j=1}^{N}{\exp }^{\beta ((1-b){v}_{i,t}+b{c}_{i,t})}}\,,$$

where *c*_*i*,*t*_ is a dummy variable indicating whether or not option *i* was chosen on the last trial (1 = chosen). *b* constrained as 0 < *b* < 1, is a repetition bonus parameter that controls the relative weight of choice repetition in the model. Because value is calculated using reward prediction errors, 0 ≤ *v*_*i*,*t*_ ≤ 1 and 0 ≤ *v*_*i*,*t*_ ≤ 1, *b* = 1 implies that the last choice is weighed as much as value, whereas *b* = 0 implies that value dominates the last choice completely.

##### Richness

One of the major differences between compare-alternatives and compare-to-threshold strategies was that only the former linearly depends on the richness of the environment, paralleling the pattern seen in humans (Fig. 2d–f). Although it does so only as a byproduct of the fact that any random option value is more likely to be above some threshold in a rich environment, it remained possible that explicitly incorporating information about richness into either the traditional RL or foraging-RL models might improve model fit. It makes intuitive sense that using richness to re-normalize values—scaling decision-noise down when options are generally good, and up when they are bad—could be a useful way to both improve overall performance and generate the kind of results seen in Fig. 2f. Therefore, we next added richness-dependent noise to both the traditional and foraging-RL models. In both foraging-RL and traditional RL models, richness was tracked through a variable *μ*_*t*_, that was updated through an exponential filter:

24 $${\mu }_{t+1}={\mu }_{t}+{\alpha }_{\mu }({r}_{t}-{\mu }_{t})\,.$$

where *r*_*t*_ is the reward received at trial *t*, independently of which option was chosen, and *α*_*μ*_ is the learning rate for richness, fitted to each participant. This means that *μ* is a choice-independent, subjective estimate of richness that depends on the actual rewards received across all options. This richness *μ* is then used to adapt the softmax decision rule by scaling the value *v* through the richness,

$$\begin{array}{l}{{{\rm{in}}}}\,{{{\rm{traditional}}}}\,{{{\rm{RL}}}},\quad p({{{{\rm{choice}}}}}_{t}=i)\to \frac{\exp ((\beta+{\beta }_{\mu }\mu ){v}_{i,t})}{\mathop{\sum }_{j=1}^{N}\exp ((\beta+{\beta }_{\mu }\mu ){v}_{j,t})}\\ {{{\rm{in}}}}\,{{{\rm{foraging}}}}-{{{\rm{RL}}}}\quad p({{{\rm{exploit}}}})\to \frac{\exp ((\beta+{\beta }_{\mu }\mu )v)}{1-\exp (-\beta (v-\rho )-{\beta }_{\mu }\mu v)}\,,\end{array}$$

where *β*_*μ*_ ≥ 0 is an inverse temperature for the specific contribution of the richness. This extension adds two additional free parameters: *α*_*μ*_  ∈ [0, 1] and *β*_*μ*_  ∈ [0, 100]. We can see from the equations that this approach remaps the inverse temperature of the standard model, *β* → *β* + *β*_*μ*_*μ*. This says that decision noise is adapting to the experienced richness. If richness is high in either model (i.e., close to 1), and *β*_*μ*_ ≠ 0, then the overall inverse temperature increases, choice curves sharpen, and the probability of choosing the highest value option goes up. In contrast, if richness is low (i.e., close to 0), the overall inverse temperature decreases, choice curves flatten, and exploratory noise increases. Because the learning rates for option value (or exploitation value) and richness can be different, this model is flexible in how it adapts to experienced richness.

#### Additional decision-making models

In addition to the models described above, we also considered both a simpler win-stay lose-shift model and a more complex dynamic belief model.

##### Win-Stay Lose-Shift model

A very simple, value-free strategy that can solve a restless multi-armed bandit is the Win-Stay Lose-Shift (WSLS) algorithm^103^. As the name suggests, the algorithm simply repeats a choice if a reward was delivered, and switches away if the reward was omitted. Because this model was only fit to 2-alternative data here, that meant switching to the only alternative option. For flexibility, in this work, we implemented an *ϵ*-greedy version of WSLS where there is a probability *ϵ* to make the opposite choice (i.e., to stay following a loss or switch following a win), and a probability 1 − *ϵ* to follow the outcome-contingent WSLS algorithm described before. In short,

$$p({{{\rm{stay}}}})=\left\{\begin{array}{ll}1-\epsilon \quad \quad &{{{\rm{if}}}}\quad r=1\hfill\\ \epsilon \hfill\quad \quad &{{{\rm{if}}}}\quad r=0,\end{array}\right.$$

where *ϵ* ∈ [0, 1]. It follows from this that *p*(switch) = 1 − *p*(stay), *p*(choice = *i*) = *p*(stay) if and only if choice *i* was made on the last trial, and $p({{{\rm{choice}}}}=i)=\frac{p({{{\rm{switch}}}})}{n-1}$ otherwise, where *n* is the number of options.

##### Dynamic belief model

Another way of solving a restless multi-armed bandit is to track a variable that signals if there has been a sudden change in the environment. One particular instantiation of this partially-observable modeling is to track a belief about the environment that changes over time, depending on the outcomes received, called The Dynamic Belief Model (DBM)^104,105^. DBM is a hidden Markov model with latent variable *γ*, which denotes the probability of observing *x* = 1. It assumes the observations are drawn from a Bernoulli distribution whose rate parameter undergoes unsignaled changes with probability 1 − *α* at each time step. Calculating the predictive probability that one will obtain a success given a past history of observations *P*_*t*+1_ ≡ *P*(*x*_*t*+1_ = 1∣*x*_1:*t*_) entails marginalizing over every possible timing of the most recent change point, or discretizing the belief space. To fit this model on our data, we followed the standard approach of discretizing the belief space (distribution over *γ*).

The standard DBM suffers from being computationally expensive due to the implicit marginalization of the probability distributions. Another possibility is to use an exponential filter approximation which works up to order *α*^2^^105^,

25 $${P}_{t+1}\approx (1-\alpha ){P}_{0}+\alpha (G{x}_{t}+{P}_{t}(1-G))\,,$$

where *P*_0_ is the initial probability of observing a reward, here chosen to be equal to 1/2 for all arms, and $G=\frac{1}{a+b+1}$, with *a* and *b* the parameters of the Beta distribution that governs the prior distribution of *γ*, the probability of observing a success^105^.

With this predictive probability, one can build an algorithm that maximizes future reward, through a decision rule like a softmax over this predictive probability for each arm:

$$P({{{\rm{choosing}}}}\,{{{\rm{arm}}}})=\frac{\exp (\beta {P}_{t+1}({{{\rm{arm}}}}))}{{\sum }_{{{{\rm{arm}}}}}\exp (\beta {P}_{t+1}({{{\rm{arm}}}}))}\,.$$

Therefore, both the full DBM and its exponential-filter approximation (EXP-DBM) have in principle 4 free parameters (*α*, *β*, *a*, *b*). However, the concentration *κ* = *a* + *b* of the Beta prior distribution governing *γ* usually gets fitted at the group level^104^. In short, *κ* measures mainly how peaked this prior distribution is, where a more peaked means stronger beliefs about what values *γ* will take if it is redrawn, values that are governed by the mean *p*_0_ = *a*/(*a* + *b*). We followed this approach and fitted *κ* at the group level (but separately for DBM and EXP-DBM), and we fitted *α*, the softmax *β*, and the prior mean *p*_0_ at the individual level, following^104^.

A different approach is to use a full exponential filter on this predictive probability *P*_*t*_, that linearly sums past observations, while exponentially discounting into the past.

$${P}_{t+1}=C(1-\delta )+\eta \delta {x}_{t}+\delta {P}_{t}\,,$$

where the free parameters (*C*, *η*, *δ*) are constrained as $0\le C,\eta \le 1,0\le \delta < 1,C+\frac{\eta \delta }{1-\delta } < 1$^105^. Along with the inverse temperature *β*, this model has thus 4 free parameters.

#### Simulations from decision-making models

Performance optimization: in order to determine if foraging-RL had a performance advantage (or disadvantage) compared to traditional RL, we compared the upper bound on performance for the two models. This meant that we used simulation to identify the optimal parameter combination for each model (minimizing the negative of the above-chance average reward; Python scipy.optimize.minimize) in randomly generated environments that matched the statistics of Experiment 1 and were matched across models. We then compared the performance of the optimized models.

Cohort simulation: when simulating data from the standard foraging-RL and traditional RL models, our goal was to produce datasets under each model that would resemble the set of human participants as closely as possible. To do this, we took the stochastic reward schedule experienced by each participant and simulated a new sequence of choices in that reward environment from the traditional RL and foraging-RL models. Simulations were generated using fitted parameters from the participant who experienced that environment.

#### Exponential mixture model

We used a mixture model to determine whether certain temporal patterns existed within the participants’ choice sequences. If a single time constant (probability of switching) governed the behavior, we would expect to see exponentially distributed inter-switch intervals. That is, the distribution of inter-switch intervals should be well described by the following model:

26 $$f(x)=\frac{1}{\beta }{\exp }^{-\frac{x}{\beta }}\,,$$

where *β* is the survival parameter of the model (mean inter-switch interval). Although the time between switch decisions was largely monotonically decreasing and concave upwards, the distribution was not well described by a single exponential distribution. Therefore, we next fit mixtures of varying numbers of exponential distributions (1–4) in order to infer the number of switching regimes in these choice processes. For continuous-time processes, these mixture distributions would be of the form:

27 $$f(x)=\mathop{\sum }_{i=1}^{n}{\uppi }_{i}{\exp }^{-\frac{x}{{\beta }_{i}}}\,,$$

where 1 ≥ *π*_*i*_ ≥ 0 for all *π*_*i*_, and $\mathop{\sum }_{i=1}^{n}{\pi }_{i}=1$. Here, each *β*_*i*_ reflects the survival parameter (average inter-switch interval) for each component distribution *i*, and the *π*_*i*_ reflects the relative weight of each component. Because trials were discrete, we fit the discrete analog of this distribution: mixtures of 1–4 discrete exponential (geometric) distributions^106^. Mixtures were fit via the expectation-maximization algorithm, and we used standard model comparison techniques^107^ to determine the most probable number of mixing components. Two regimes (log-likelihood = −31,048, 3 parameter) were significantly better fit to data than one (log-likelihood = −34,645, 1 parameter, likelihood ratio test: ratio = 7193.0, *p* < 0.001) and, while adding additional regimes continued to improve model fit (3 regimes: log-likelihood = −30,893, 5 parameters; 4 regimes: log-likelihood = −30,884), improvement was already saturated at 2 regimes (Fig. 5f and Supplementary Table 3).

#### Hidden Markov model

To determine when models and participants were exploring (versus exploiting), we used a hidden Markov model (HMM)^15,24,36,40,41^. In an HMM, choices (*y*) are ‘emissions’ that are generated by an unobserved decision process that is in some latent, hidden state (*z*). Latent states are defined by both the probability of making each choice (*k*, out of *N*_*k*_ possible options), and by the probability of transitioning from each state to every other state. Our model consisted of two types of states, an explore state and the exploit states. The emissions model for the explore state was the maximum-entropy distribution for a categorical variable, a uniform distribution:

28 $$p({y}_{t}=k| {z}_{t}={{{\rm{explore}}}})=\frac{1}{{N}_{k}}\,,$$

meaning that we made no assumptions about the choices that were made during exploration in order to avoid biasing the model towards or away from any particular type of policy. Notes that modeling exploratory choices with a uniform distribution does not imply, require, or enforce random decision-making during these states^40,41^. Because exploitation involves repeated sampling of each option, exploit states only permitted choice emissions that matched one option:

29 $$\begin{array}{l}p({y}_{t}=k| {z}_{t}={{{{\rm{exploit}}}}}_{i},k=i)=1\\ p({y}_{t}=k| {z}_{t}={{{{\rm{exploit}}}}}_{i},k\ne i)=0\end{array}\,,$$

meaning latent states in this model are Markovian. The current state (*z*_*t*_) depends only on the most recent state (*z*_*t*−1_),

30 $$p({z}_{t}| {z}_{t-1},{y}_{t-1},...,{z}_{1},{y}_{1})=p({z}_{t}| {z}_{t-1})\,,$$

meaning that we can describe the entire pattern of dynamics in terms of a time-invariant transition matrix between 3 possible states (two exploit states and one explore state). This matrix is a system of stochastic equations that describe the one-time-step probability of transitioning between every combination of past and future states (*i*, *j*),

31 $$p({z}_{t}=i| {z}_{t-1}=j)\,.$$

Because we had only a limited number of trials for each participant (300), parameters were tied across exploit states: each exploit state had the same probability of beginning (from exploring) and of sustaining itself. For conceptual reasons, the model also assumed that participants started in exploration and had to pass through exploration in order to start exploiting a new option, even if only for a single trial^15,24,36,40,41^. We have previously shown that models that lack these constraints by design tend to approximate them when fit to sufficiently large datasets^15,24^.

Because the emissions model was fixed, certain parameters were tied, the structure of the transmission matrix was constrained, and the initial state was specified. The final HMM had only two free parameters: one corresponding to the probability of exploring, given exploration on the last trial, and one corresponding to the probability of exploiting, given exploitation on the last trial. We have previously reported that this constrained model does not underperform an unconstrained model^15,24^. and that unconstrained models tend to closely resemble the constrained model when fit to large amounts of data^15^.

The HMM was fit via expectation-maximization using the Baum-Welch algorithm^108^. For Experiment 1, this algorithm finds a (possibly local) maxima of the complete-data likelihood. The algorithm was reinitialized with random seeds 20 times, and the model that maximized the observed (incomplete) data log likelihood across all the sessions for each participant was ultimately taken as the best. To decode latent states from choices, we used the Viterbi algorithm to discover the most probable a posteriori sequence of latent states. For Experiment 3, the high volatility of the environment resulted in some participants never being classified as entering the exploitation state, thereby violating our initial assumptions. In these cases, we re-seeded the analysis for those specific sessions (exploit-to-exploit transition < 0.01). If no new local minimum could be found, the session was excluded. This meant that 39/270 participants were excluded from HMM-related analyses in Experiment 3.

#### Explore/exploit state dynamics

In order to understand the dynamics of exploration and exploitation in the participants, traditional RL models, and foraging-RL models, we analyzed the dynamics of HMMs fit to each dataset^24,36,41^. The fitted HMMs are a set of equations that describe the probability of transitions between exploration and exploitation and vice versa: the state dynamics of each agent. Methods from statistical thermodynamics can then be used to analyze these equations and generate insight into the potential energy of each state in each class of agent (Fig. 4m).

In statistical thermodynamics, the potential energy of a state (*E*_*i*_) is related to the long-time scale probability of observing a process (here, a decision-maker) in that state (*p*_*i*_) via the Boltzmann distribution,

32 $${p}_{i}=\frac{1}{Z}{exp}^{\,\frac{-{E}_{i}}{{k}_{B}T}} \,,$$

where *Z* is the partition function of the system, *kB* is the Boltzmann constant, and *T* is the temperature. In a two-state system, the partition functions cancel out, the relative occupancy of the states is just a function of the difference in energy between them, and we can rearrange to express the difference in energy between two states as a function of the difference between them,

33 $$\,{{{\rm{ln}}}}\,\left(\frac{{p}_{i}}{{p}_{j}}\right){k}_{B}T={E}_{j}-{E}_{i}\,,$$

where *E*_*j*_ − *E*_*i*_ is the difference in the energetic depth between the two states (i.e., the Gibbs Free Energy), which is proportional to the log odds of each state, up to some multiplicative factor, *kBT*.

To calculate the probability of exploration and exploitation (*p*_*i*_ and *p*_*j*_), we solved for the stationary distribution π^*^ of the fitted HMMs, where π^*^ is the probability distribution that satisfies

34 $${\uppi }^{*}={\uppi }^{*}{A}_{k}\,,$$

where *A*_*k*_ is the transition matrix for agent *k*. If it exists, this distribution is a (normalized) left eigenvector of *A*_*k*_ with an eigenvalue of 1, so we solved for this eigenvector to determine the stationary distribution over explore and exploit states for each agent. We then took an average of these stationary distributions across all sessions for each species, and plugged these back into the Boltzmann equations to calculate the relative energy (depth) of exploration and exploitation (Fig. 4m).

In order to calculate the height of the energetic barrier between exploration and exploitation, we built on the Arrhenius equation from chemical kinetics that relates the rate of transitions (*k*) between some pair of states to the activation energy required to affect these transitions (*E*_*a*_*)*:

35 $$k=C{exp}^{\frac{{E}_{a}}{{k}_{B}T}} \,,$$

where *C* is a constant pre-exponential factor and *kBT* is again the product of temperature and the Boltzmann constant. Rearranging to solve for activation energy yields our equation for the height of the energy barrier,

36 $${E}_{a}=- \, {{{\rm{ln}}}} \, \left(\frac{k}{C}\right){k}_{B}T \,,$$

which has an obvious similarity to the Boltzmann distribution illustrated earlier, where the relative depth of each state was proportional to the probability of occupying each state and the activation energy is now proportional to the rate of transitions between states.

To create the dynamical landscape graphs (Fig. 4m), transition matrices were calculated individually for all participants and for simulated data from both compare-to-threshold and compare-alternatives algorithms. Energy measurements were then averaged within each class of agents. Note that our approach only identifies the energy of three discrete states (an explore state, an exploit state, and the peak of the barrier between them). These are illustrated by tracing a continuous potential through these three points to provide a physical intuition for the differences in explore/exploit dynamics between models and participants.

#### Foraging index

In order to determine if individual participants were more foraging-like (compare-to-threshold) in their approach to the task, we calculated the compare-to-threshold index as the difference in the individual model likelihood (${{{\mathcal{L}}}}$) between the best-fitting foraging-RL model (Thres*) and the best-fitting traditional RL model (Alt*) for each participant i of Experiment 1:

37 $${{{{\rm{Foraging}}}}}\,{{{\rm{index}}}}_{i}={{{{\mathcal{L}}}}}_{Thre{s}^{*},i}-{{{{\mathcal{L}}}}}_{Al{t}^{*},i}$$

### Reporting summary

Further information on research design is available in the Nature Portfolio Reporting Summary linked to this article.

## Supplementary information


Supplementary Information
Reporting Summary
Transparent Peer Review file


## Data Availability

The behavioral data, model fits, and simulation outputs generated and analyzed in this study are available on Figshare at https://doi.org/10.6084/m9.figshare.32193990^109^.

## Code availability

The scripts and Jupyter notebooks used to generate the data, conduct the analyses, and reproduce the figures in this manuscript are available in the following repository: https://doi.org/10.5281/zenodo.20184471^110^.
