# Supplementary material for: Foraging models explain human exploration in uncertain tasks
Source: Nat Commun. 2026 Jul 31;17:9292. doi: 10.1038/s41467-026-75773-4 (PMC13526786; doi:10.1038/s41467-026-75773-4)
Supplement: Supplementary file 1 [file 41467_2026_75773_MOESM1_ESM.pdf]

# Supplementary Information

# Supplementary Figures

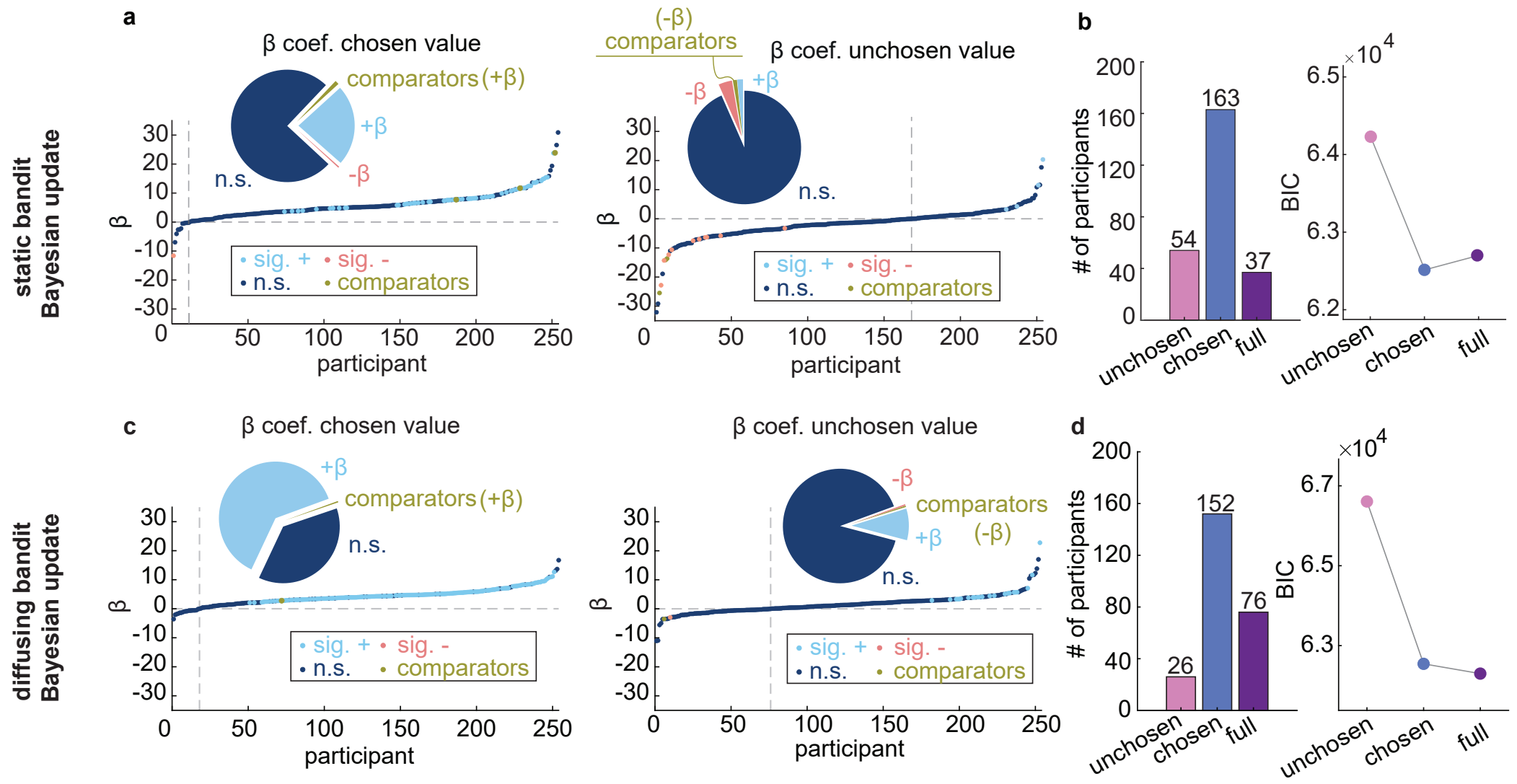


**Figure S1:** Participant largely ignored the subjective value of the unchosen option. In Figure 1.f-g we used objective reward probabilities as proxies for option values; however, since these are only partially observable to the participants, another approach would be to use an Ideal Bayesian Learner (IBL) to estimate subjective values from the sampling history. The subjective values were estimated either via a parameter-free IBL that perfectly integrates the sampling history over time (static bandit bayesian update, a-b) or via an IBL that knows how the values drift over time (without observing them) and optimal decays the subjective values due to diffusion (diffusing bandit Bayesian update, c-d). See Choice predictive Generalized Linear Model (GLM) section of the Methods for more details. a/c) Predicting choice from the value of chosen and unchosen options. Beta coefficients for ($V(\text{chosen}_{t-1})$) (left) and $V(\text{unchosen}_{t-1})$ (right). Each circle represents a single participant, sorted by coefficient value. The dashed horizontal line is at zero (no effect). Participants with a significant positive betas are light blue, significant negative betas in red, and non-significant (n.s.) betas in dark blue. Gold circles identify "comparators" who had a significant positive weight for the chosen option and negative weight for unchosen option value. Significance is Holm-Bonferroni corrected within predictor ($\alpha = 0.05$). Insets illustrate the proportion of participants in whom each term was significant, colored by sign and identifying all "comparators". b/d) (left) Bayesian information criterion (BIC) for each model ($V(\text{unchosen}_{t-1})$-only (pink), $V(\text{chosen}_{t-1})$-only (blue), and the full model (purple) which contains both terms). (right) The number of individual participants best fit by each model, based on the BIC.

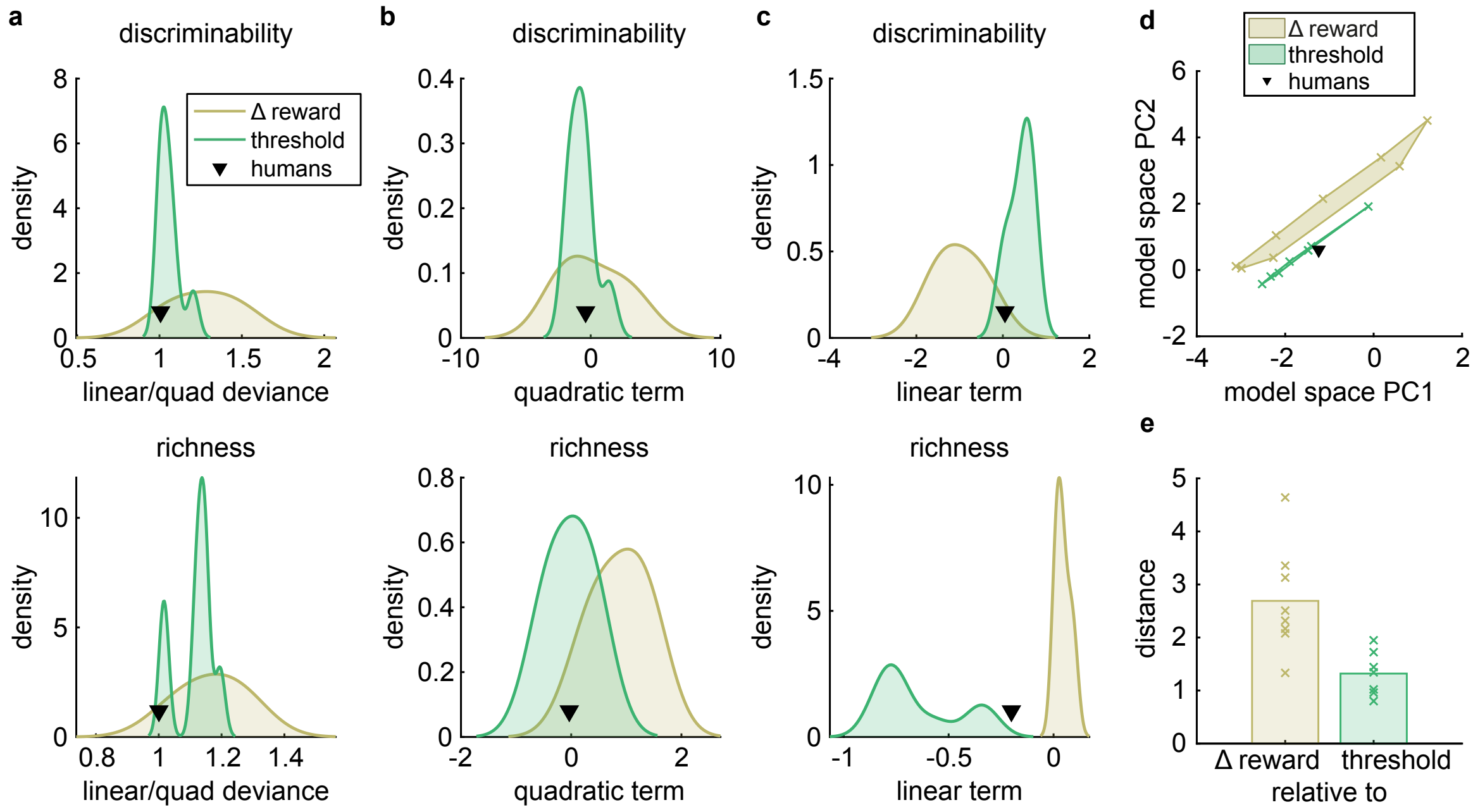


**Figure S2:** Quantifying the fingerprints of compare-to-threshold and compare-alternatives decision to explore. a) Distribution of deviance ratio between linear and quadratic model fits to compare-alternatives (gold) and compare-to-threshold (green) environmental predicted probability of switch (Figure 2.d-e) as a function of the discriminability(top) and richness (bottom) of the environment (black caret = humans). b) Distribution of the quadratic term (i.e., curvature) of the quadratic model fit to compare-alternatives (gold) and compare-to-threshold (green) environmental predicted p(switch) (Figure 2.d-e) as a function of discriminability(top) and richness (bottom) of the environment (black caret = humans). c) Distribution of the linear term (i.e., slope) of the linear model fit to compare-alternatives (gold) and compare-to-threshold (green) environmental predicted probability of switch (Figure 2.d-e) as a function of discriminability(top) and richness (bottom) (black caret = humans). d) Same as Figure 2.g, but for the linear GLM, rather than the binomial model. First 2 PCs of the multivariate feature space of compare-alternatives fingerprints (gold) and compare-to-threshold fingerprints (green). Bounds encompass all values for the $\Delta reward$ and threshold parameters. x = each parameter value, black caret = humans. e) Same as Figure 2.h, but for the linear GLM (identity link function), rather than the binomial model. Distance between humans and every (gold) and (green) in the full 7-dimensional space. Significant paired t-test : $p < 0.001$, Bars = mean distance, x = individual parameter values.

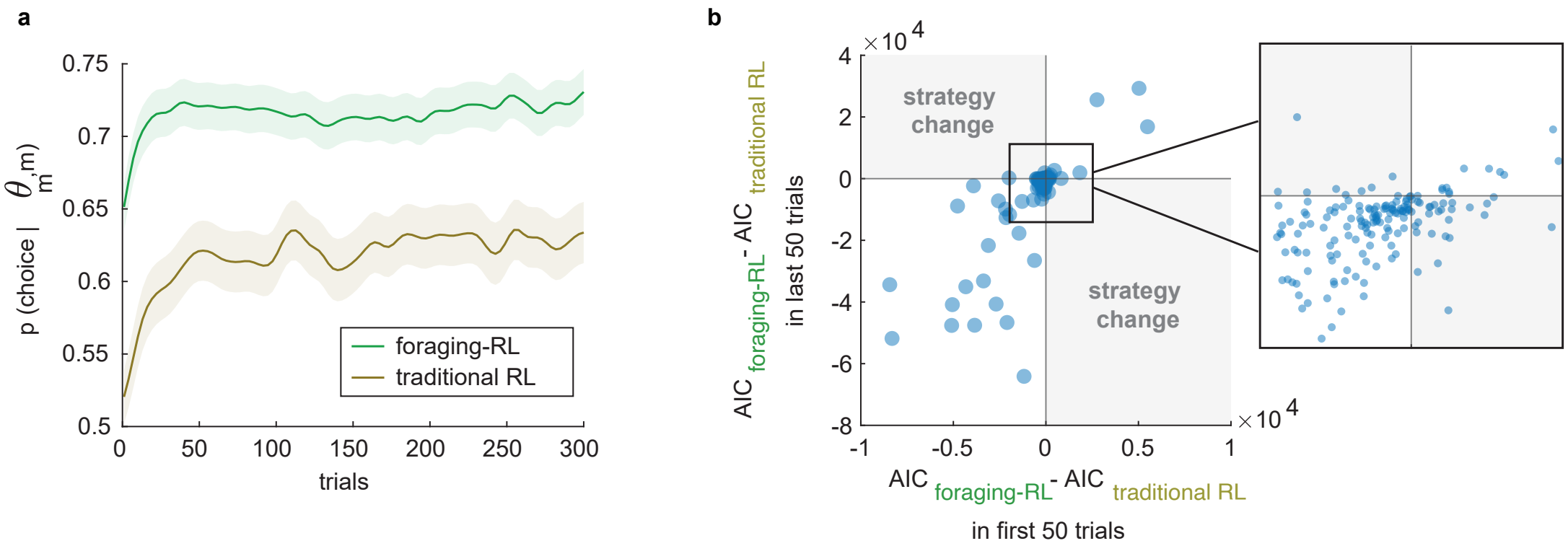


**Figure S3:** Stability of model fits across the sessions. a) Choice likelihood for each model as a function of trial number. Lines represent smoothed mean (bin = 5) and shading represent $\pm SEM$ across participants in Experiment 1.b) To further explore whether people switched strategies, we compared model fit in the first 50 trials versus the last 50 trials of each session. For each participant, we calculated the AIC for both epochs and then computed the difference in AIC (foraging-RL - traditional RL). Each dot represents the AIC difference in the last 50 trials as a function in the first 50 trials. Participants who switched strategies would fall into either gray areas in the plot. We found that some participants did go from preferring one strategy to preferring another (42 / 254; 16.5%). However, the difference in relative evidence for the two models was an order of magnitude lower in these strategy switching participants (50 first trials; all participants mean $\Delta AIC$ [foraging - traditional] = -263.02 ; participants who switched strategies median $\Delta AIC$ [foraging - traditional] = -38.45; 50 last trials; all participants median $\Delta AIC$ [foraging - traditional] = -2306 ; participants who switched strategies mean $\Delta AIC$ [foraging - traditional] = -121). This makes it difficult to determine how many of these participants truly switched strategies between the beginning and end of the task, versus how many had ambiguous evidence for the models in one or the other 50 trial bin and only appeared to switch due to noise in model fitting. Inset = zoomed in.

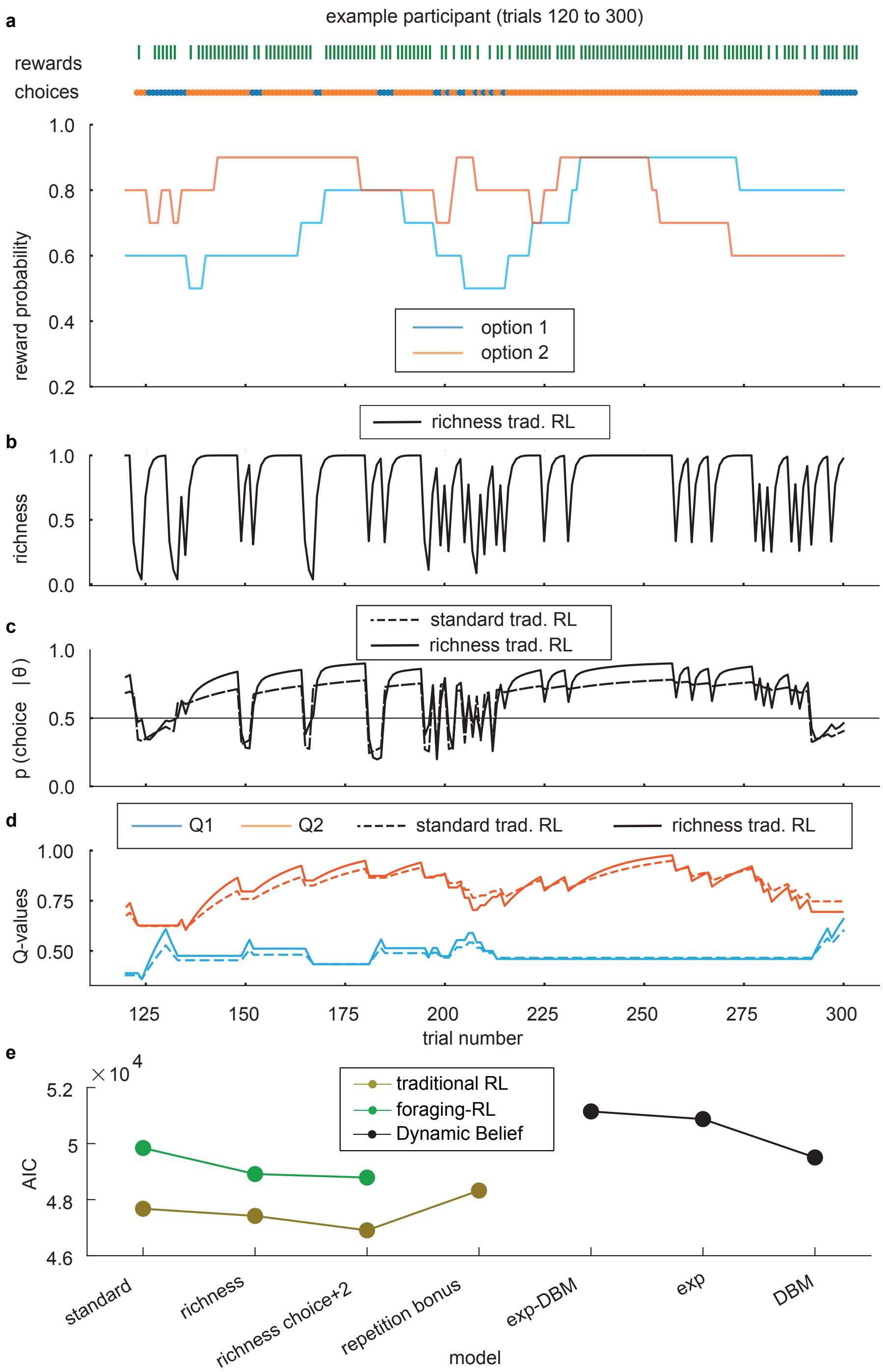


**Figure S4:** Additional model fits. a) Example reward probabilities from a single participant in Experiment 1. Trials 120 to 300 are shown for illustration. Color of the circles on top correspond to the option chosen by the participant. Green ticks indicate that the participant received reward on that trial, and red ticks indicate omission of reward. b) Richness, as estimated by the richness traditional RL model, with maximum-likelihood parameters that best explain the tracking of richness under this model. c) The likelihood of fitted standard traditional RL (standard trad. RL; dotted) and richness traditional RL (richness trad. RL; solid) models for each choice made by the participant. Chance at 0.5. Richness trad. RL has a better likelihood because the richness allows this model to have a more deterministic choice than standard-RL, even with similar Q-values.d) Q-values calculated by each model (see Methods). e) Akaike information criterion (AIC) values (n = 254) for additional extensions of traditional RL and foraging-RL models, fits to behavior from Experiment 1. Richness choice models incorporate both choice history dependence and richness terms (see Methods). exp-DBM is the exponential filtering approximation to the Dynamic belief model (Ryali et al. 2018, see Methods), exp is a fully fitted exponential filter (Ryali et al. 2018) and DBM is the Dynamic Belief model as in (Guo et al. 2018).

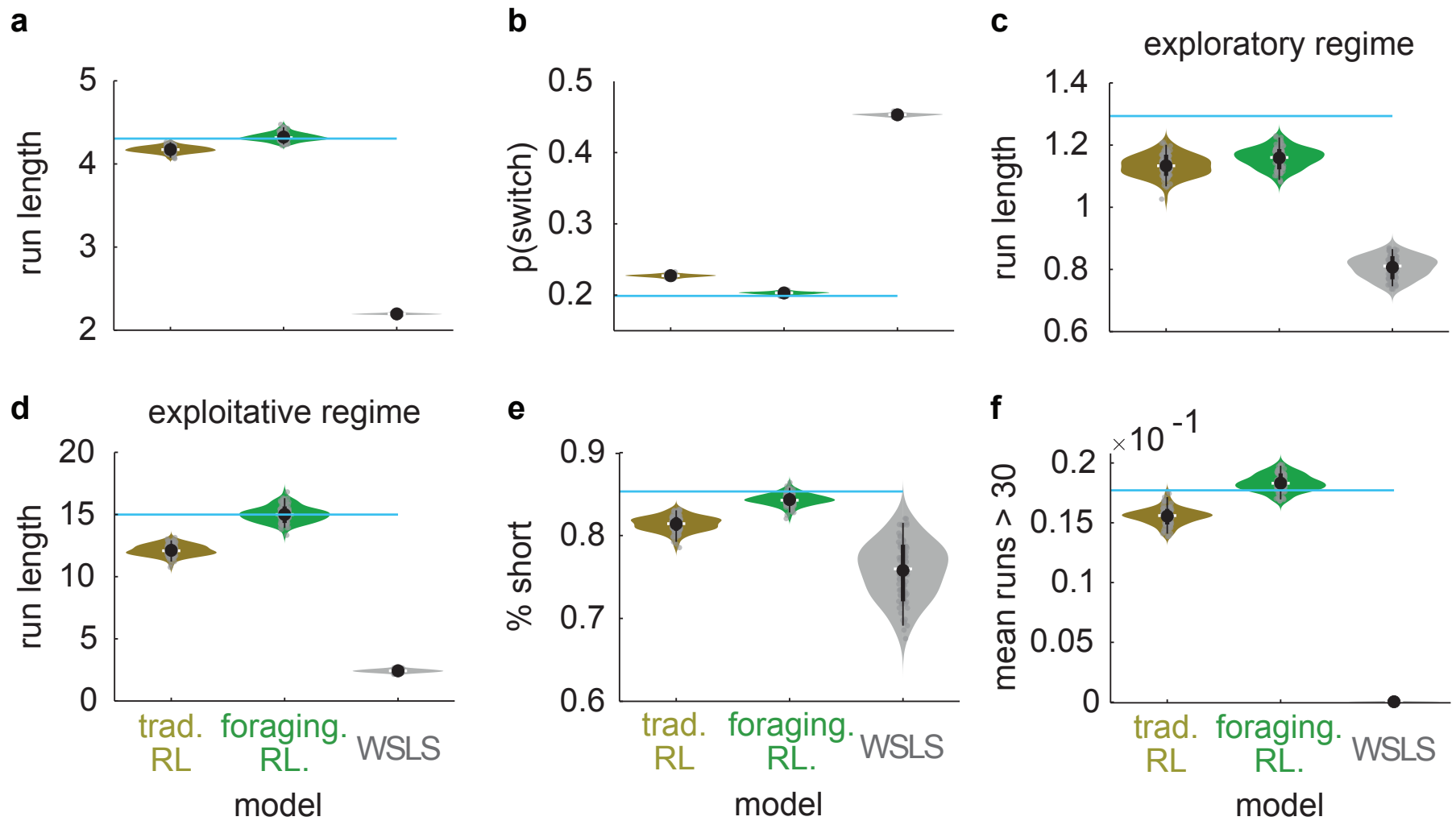


**Figure S5:** traditional RL and foraging-RL models predict behavior more accurately than a simple win-stay lose-shift model a) Average number of trials between switch decisions (i.e., run length) of traditional RL (gold) and foraging-RL (green) and win-stay lose-shift (grey) agents in simulated Experiment 1 (blue line = average for Experiment 1 participants, black dot = mean, black line = IRQ, n = 100). Same conventions as in a, in panels b-f. b) Probability of switch. c) Average run length in the exploratory regime. d) Average run length in the exploitative regime. e) Proportion of exploratory regimes. f) Proportion of very long exploitative regimes exceeding 30 consecutive trials.

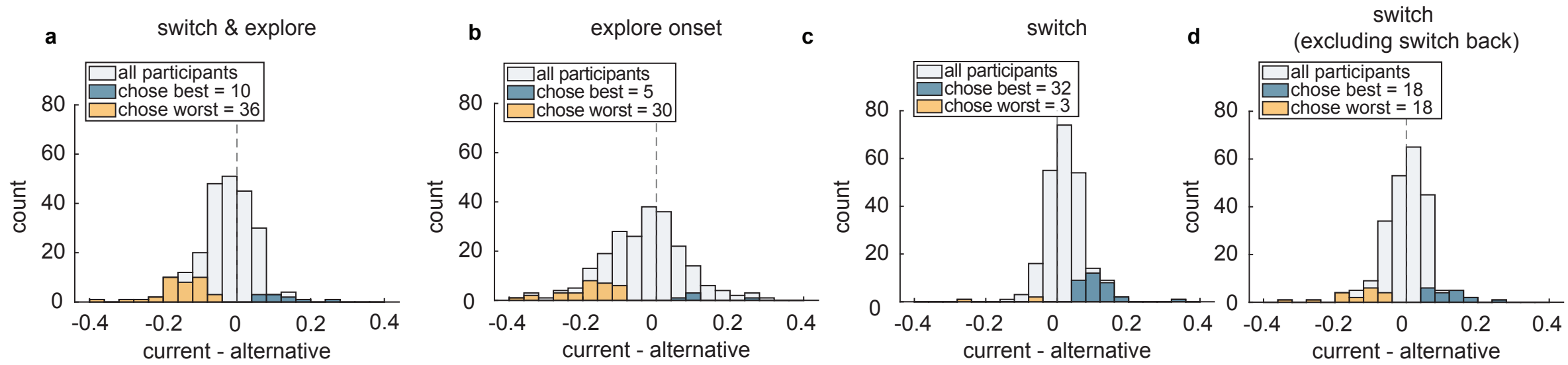


**Figure S6:** Switching preferences among the alternative options in Experiment 3. a) Because 3 options were used in Experiment 3, we were able to ask if participants preferentially switched to the best or worst of the alternatives. We first focused on exploratory switches: switch decisions that were also identified as exploratory via the HMM (see Methods). Distributions (grey) show the average difference in objective option values for these decisions within each participant. Positive values indicate participants who switched to the objectively better alternative, negative values indicate participants who switched to the objectively worse alternative. Participants that chose the best alternative significantly more frequently than chance are in blue. Participants that chose the worst alternative more frequently than chance are in orange. Chance was estimated by running 50,000 bootstrap simulations of random choices for each individual, significance tested at $p < 0.05$, uncorrected. b) Same as a, but specifically for the first explore trial in a sequence (onset of exploration) which is a switch decision by definition. c) Same as a, but for all switches, many of which are switch backs, returning to the previously exploited option.d) Same as c, but excluding the switch backs to the last exploited option.

# Supplementary Tables

| | model | total parameters ($k$) | AIC | log-likelihood | $AIC_w$ |
|---|---|---|---|---|---|
| **traditional RL** | repetition bonus | 762 | 48323.8 | -23,399.9 | < 0.001 |
| | richness | 1016 | 48,913.3 | -23,440.7 | < 0.001 |
| | richness and history kernel 2 | 1524 | 48,787.7 | -22,869.8 | < 0.001 |
| **dynamic belief** | exp-DBM | 508 | 53,150 | -26,067 | < 0.001 |
| | DBM | 508 | 51,025.9 | -25,004 | < 0.001 |
| | exp | 1016 | 50,873 | -24,420 | < 0.001 |

**Supplementary Table 1: Pairwise, group-level model comparison against standard foraging-RL for Experiment 1**. AIC weights are computed relative to standard foraging-RL ($AIC_w$ for standard foraging-RL was 1 in all pairwise comparisons).

| extension | model | total parameters ($k$) | AIC | log-likelihood | $AIC_w$ |
|---|---|---|---|---|---|
| **asymmetrical** | foraging-RL | 1016 | 47,585 | -22,777 | 1 |
| | traditional RL | 762 | 49,450 | -23,963 | < 0.001 |
| **decaying** | foraging-RL | 1016 | 47,295 | -22,632 | 1 |
| | traditional RL | 762 | 47,887 | -23,181 | < 0.001 |
| **history kernel 1** | foraging-RL | 1016 | 47,148 | -22,558 | 1 |
| | traditional RL | 762 | 48,028 | -23,252 | < 0.001 |
| **history kernel 2** | foraging-RL | 1270 | 47,125 | -22,292 | 1 |
| | traditional RL | 1016 | 47,351 | -22,660 | < 0.001 |
| **richness** | foraging-RL | 1270 | 47,419.4 | -22,439.7 | 1 |
| | traditional RL | 1016 | 48,913.3 | -23,440.7 | < 0.001 |
| **richness history kernel 2** | foraging-RL | 1778 | 46,904.3 | -21,674.2 | 1 |
| | traditional RL | 1524 | 48,787.7 | -22,869.8 | < 0.001 |

**Supplementary Table 2: Group-level class comparison for Experiment 1.** AIC weights are computed relative to the equivalent foraging-RL model.

| group | regimes | Log-likelihood | parameters | n |
|---|---|---|---|---|
| participants | 1 | -34,645 | 1 | 14,847 |
| | 2 | -31,048 | 3 | 14,847 |
| | 3 | -30,893 | 5 | 14,847 |
| | 4 | -30,884 | 6 | 14,847 |
| traditional RL | 1 | -3,907,700 | 1 | 1,699,789 |
| | 2 | -3,527,300 | 3 | 1,699,789 |
| | 3 | -3,502,000 | 5 | 1,699,789 |
| | 4 | -3,500,600 | 6 | 1,699,789 |
| foraging-RL | 1 | -3,550,000 | 1 | 1,517,656 |
| | 2 | -3,128,100 | 3 | 1,517,656 |
| | 3 | -3,105,700 | 5 | 1,517,656 |
| | 4 | -3,103,900 | 6 | 1,517,656 |

**Supplementary Table 3: Model comparison for switching regimes across participants and simulated agents.** The 2 regime model provided a significantly better fit than the 1 regime model for all groups (likelihood ratio test; participants : $ratio = 7193.0, p < 0.001$; traditional RL : $ratio = 760,950, p < 0.001$ ; foraging-RL : $843,650, p < 0.001$), no significant improvement was found from 2 to 3 and from 3 to 4 regimes.

| volatility | model | total parameters ($k$) | AIC | log-likelihood |
|---|---|---|---|---|
| **low** | foraging-RL | $3 \times 90$ | 34,441 | -16,950.5 |
| | traditional RL | $2 \times 90$ | 36,159.2 | -17,899.6 |
| **medium** | foraging-RL | $3 \times 91$ | 38,931 | -19,192.5 |
| | traditional RL | $2 \times 91$ | 40,037.3 | -19,836.7 |
| **high** | foraging-RL | $3 \times 88$ | 39,122.2 | -19,297.1 |
| | traditional RL | $2 \times 88$ | 41,280.1 | -20,464.1 |

**Supplementary Table 4: Group-level model comparison for Experiment 3.**

| # of arms | model | total parameters ($k$) | AIC | log-likelihood |
|---|---|---|---|---|
| **2-armed** | foraging-RL | $3 \times 50$ | 11,371.3 | -5,535.7 |
| | traditional RL | $2 \times 50$ | 11,718.7 | -5,759.4 |
| **3-armed** | foraging-RL | $3 \times 47$ | 14,572.1 | -7,145.1 |
| | traditional RL | $2 \times 47$ | 15,027.1 | -7,419.5 |
| **4-armed** | foraging-RL | $3 \times 50$ | 16,070.7 | -7,885.4 |
| | traditional RL | $2 \times 50$ | 16,562.2 | -8,181.1 |

**Supplementary Table 5: Group-level model comparison for Experiment 4.**

| presentation order | response modality | model | total parameters ($k$) | AIC | -log lik |
|---|---|---|---|---|---|
| **eye-tracking first** | **eye-tracking (1$^{st}$)** | foraging-RL | $3 \times 16$ | 7,523.5 | -3,758.7 |
| | | traditional RL | $2 \times 16$ | 7,596.3 | -3,796.1 |
| | **touchscreen (2$^{nd}$)** | foraging-RL | $3 \times 16$ | 7,221.4 | -3,607.7 |
| | | traditional RL | $2 \times 16$ | 7,470.0 | -3,733.0 |
| **touchscreen first** | **eye-tracking (2$^{nd}$)** | foraging-RL | $3 \times 13$ | 5,101.1 | -2,547.6 |
| | | traditional RL | $2 \times 13$ | 5,218.1 | -2,607.0 |
| | **touchscreen (1$^{st}$)** | foraging-RL | $3 \times 13$ | 5,086.7 | -2,540.4 |
| | | traditional RL | $2 \times 13$ | 5,256.7 | -2,626.3 |

**Supplementary Table 6: Group-level model comparison for Experiment 5.**
