# Supplementary material for: Foraging models explain human exploration in uncertain tasks
Source: Nat Commun. 2026 Jul 31;17:9292. doi: 10.1038/s41467-026-75773-4 (PMC13526786; doi:10.1038/s41467-026-75773-4)
Supplement: Supplementary file 2 [file 41467_2026_75773_MOESM2_ESM.pdf]

nature portfolio

Corresponding author(s): Meriam Zid, Becket Ebitz, Alexander Herman

Last updated by author(s): May 25, 2026

Reporting Summary

Nature Portfolio wishes to improve the reproducibility of the work that we publish. This form provides structure for consistency and transparency in reporting. For further information on Nature Portfolio policies, see our Editorial Policies and the Editorial Policy Checklist.

Statistics

For all statistical analyses, confirm that the following items are present in the figure legend, table legend, main text, or Methods section.

n/a	Confirmed	
☐	☒	The exact sample size (*n*) for each experimental group/condition, given as a discrete number and unit of measurement
☐	☒	A statement on whether measurements were taken from distinct samples or whether the same sample was measured repeatedly
☐	☒	The statistical test(s) used AND whether they are one- or two-sided<br>*Only common tests should be described solely by name; describe more complex techniques in the Methods section.*
☐	☒	A description of all covariates tested
☒	☐	A description of any assumptions or corrections, such as tests of normality and adjustment for multiple comparisons
☐	☒	A full description of the statistical parameters including central tendency (e.g. means) or other basic estimates (e.g. regression coefficient) AND variation (e.g. standard deviation) or associated estimates of uncertainty (e.g. confidence intervals)
☐	☒	For null hypothesis testing, the test statistic (e.g. *F*, *t*, *r*) with confidence intervals, effect sizes, degrees of freedom and *P* value noted<br>*Give P values as exact values whenever suitable.*
☒	☐	For Bayesian analysis, information on the choice of priors and Markov chain Monte Carlo settings
☒	☐	For hierarchical and complex designs, identification of the appropriate level for tests and full reporting of outcomes
☐	☒	Estimates of effect sizes (e.g. Cohen's *d*, Pearson's *r*), indicating how they were calculated

Our web collection on statistics for biologists contains articles on many of the points above.

Software and code

Policy information about availability of computer code

Data collection	The data for the experiments 1-4 were collected using Amazon Mechanical Turk (mTurk).<br>Participants were required to have a minimum of 5000 approved HITs and a 98% approval rating to ensure data quality. The data for the experiment 5 was collected in-person.
Data analysis	Data was analyzed using custom software written in MATLAB and Python. Traditional RL and foraging-RL models were fitted to the data using maximum likelihood estimation. Code to generate the results and various figures is available at https://doi.org/10.5281/zenodo.20184471.

For manuscripts utilizing custom algorithms or software that are central to the research but not yet described in published literature, software must be made available to editors and reviewers. We strongly encourage code deposition in a community repository (e.g. GitHub). See the Nature Portfolio guidelines for submitting code & software for further information.

Data

Policy information about availability of data

All manuscripts must include a data availability statement. This statement should provide the following information, where applicable:

- Accession codes, unique identifiers, or web links for publicly available datasets
- A description of any restrictions on data availability
- For clinical datasets or third party data, please ensure that the statement adheres to our policy

All data generated in this study have been deposited in the figshare database under https://doi.org/10.6084/m9.figshare.32193990.

Research involving human participants, their data, or biological material

Policy information about studies with human participants or human data. See also policy information about sex, gender (identity/presentation), and sexual orientation and race, ethnicity and racism.

Reporting on sex and gender	Sex and gender were considered to the extent that each study had a balanced sample of self-identified male and female participants, no sex and gender-based analyses were reported here because these are a topic for future research.
Reporting on race, ethnicity, or other socially relevant groupings	We did not collect any information on race and ethnicity.
Population characteristics	For Exp. 1-4, population was US-based MTurk workers. For Exp. 5, population was Canadian-based.
Recruitment	For Exp. 1-4, participants were recruited via Amazon mTurk. For Exp. 5, participants recruited via flyers.
Ethics oversight	ll experiments procedures were in line with the standards set by the Declaration of Helsinki and were approved by the relevant ethical review boards. Experiment 1 was approved by the Comité d'Éthique de la Recherche en Sciences et Santé (CERSES) of the University of Montreal [Project #2021-1090]. Experiment 2 was approved by the CERSES of the University of Montreal [Project #2021-1090]. Experiment 3 was approved by the Institutional Review Board of the University of Minnesota [STUDY00008486]. Experiment 4 was approved by Princeton University Institutional Review Board [IRB #7873]. Experiment 5 was approved by the CERSES of the University of Montreal [Project #2021-1090].

Note that full information on the approval of the study protocol must also be provided in the manuscript.

Field-specific reporting

Please select the one below that is the best fit for your research. If you are not sure, read the appropriate sections before making your selection.

☐ Life sciences ☒ Behavioural & social sciences ☐ Ecological, evolutionary & environmental sciences

For a reference copy of the document with all sections, see nature.com/documents/nr-reporting-summary-flat.pdf

Behavioural & social sciences study design

All studies must disclose on these points even when the disclosure is negative.

Study description	This study uses a quantitative data. Participants performed a restless k-armed bandit task (1 of 5 variations), and computational models (foraging-like and traditional RL models) were fit to their behavior. The study compares model fits across conditions/ participants combined with model-free approaches to characterize human sequential decision making.
Research sample	For Exp. 1-4, participants were recruited via Amazon Mechanical Turk (mTurk). The sample included 258 participants for Experiment 1, For Experiment 2, a total of 95 participants (41 females, 51 males) completed the task. For Experiment 3, a total of 270 participants (112 females, 156 males, 2 prefer not say) completed the task. For Experiment 4, a total of 150 participants (sex and gender was not collected) completed the task. For Experiment 5 (in person experiment), a total of 29 participants (19 females, 10 males) completed the task.
Sampling strategy	Samples sizes were chosen to be sufficiently powered to detect expected effect sizes of interest (1 - beta = 0.8).
Data collection	Data collection involved participants completing tasks on the mTurk platform (Experiment 1- 4) or in person (Experiment 5) Participants first completed a practice block followed by the main task (1 of 5 variations of a restless k-armed bandit).
Timing	Data collection occured between 9am and 5pm (eastern time zone) from monday to friday.
Data exclusions	Participants were excluded from model fitting if they chose only one option throughout the task, making model parameters unidentifiable. Specifically, 1.6% of participants in Experiment 1, 0.95% in Experiment 2, 0.4% in Experiment 3 and 2% in Experiment 4 excluded for this reason. However, these participants were included in all other analyses, including analyses that focused on these participants. In Experiment 3, 14% of participants were excluded from 1 analysis because explore and exploit states were unidentifiable.
Non-participation	All recruited participants completed the tasks as intended.
Randomization	The reward probabilities experienced by each participant were randomized.

Reporting for specific materials, systems and methods

We require information from authors about some types of materials, experimental systems and methods used in many studies. Here, indicate whether each material, system or method listed is relevant to your study. If you are not sure if a list item applies to your research, read the appropriate section before selecting a response.

Materials & experimental systems

n/a	Involved in the study
☒	☐ Antibodies
☒	☐ Eukaryotic cell lines
☒	☐ Palaeontology and archaeology
☒	☐ Animals and other organisms
☒	☐ Clinical data
☒	☐ Dual use research of concern
☒	☐ Plants

Methods

n/a	Involved in the study
☒	☐ ChIP-seq
☒	☐ Flow cytometry
☒	☐ MRI-based neuroimaging

Plants

Seed stocks	*Report on the source of all seed stocks or other plant material used. If applicable, state the seed stock centre and catalogue number. If plant specimens were collected from the field, describe the collection location, date and sampling procedures.*
Novel plant genotypes	*Describe the methods by which all novel plant genotypes were produced. This includes those generated by transgenic approaches, gene editing, chemical/radiation-based mutagenesis and hybridization. For transgenic lines, describe the transformation method, the number of independent lines analyzed and the generation upon which experiments were performed. For gene-edited lines, describe the editor used, the endogenous sequence targeted for editing, the targeting guide RNA sequence (if applicable) and how the editor was applied.*
Authentication	*Describe any authentication procedures for each seed stock used or novel genotype generated. Describe any experiments used to assess the effect of a mutation and, where applicable, how potential secondary effects (e.g. second site T-DNA insertions, mosiacism, off-target gene editing) were examined.*
