# Supplementary material for: Foraging models explain human exploration in uncertain tasks
Source: Nat Commun. 2026 Jul 31;17:9292. doi: 10.1038/s41467-026-75773-4 (PMC13526786; doi:10.1038/s41467-026-75773-4)
Supplement: Supplementary file 3 [file 41467_2026_75773_MOESM3_ESM.pdf]

nature portfolio

Peer Review File

The singular value: How humans explore in uncertain tasks.

Corresponding Author: Mx Meriam ZID

This file contains all reviewer reports in order by version, followed by all author rebuttals in order by version.

Parts of this Peer Review File have been redacted as indicated to remove third-party material.

Version 0:

Reviewer comments:

Reviewer #1

(Remarks to the Author)
This manuscript is about modelling classical drifting bandit tasks, not as a comparison between independent, learned value estimates, but instead a single variable that tracks an option unspecific recency weighted reward history to determine switching based on a threshold comparison with the current reward estimate of staying.
Overall, this is very interesting idea that seems to have been in the air for some time and boils down to people having a sense of what reward level constitutes a "good" option and not exploring unless the option falls below a certain level (strictly speaking the average reward rates, but as participants have long stretches of exploitation it often boils down to the same thing).
I liked the idea to use tasks that favour option comparison mindset (or are at least primarily modelled like that) to test their switch vs stay decision making models, but the current set of experiments and weight of evidence, do not, at this point, fully support their very strong claims of a lack of option based comparisons in favour of foraging / switch vs stay decision making, both due to the problematic nature of either/or statements from the data and because only a limited set of behaviours were tested.

Below I have listed my specific concerns point by point (grouped into minor and major):

1) Fundamentally, the notion that humans either ONLY do option specific comparisons or foraging like switch stay decisions is unlikely to be true. Furthermore, this conclusion is not supported by the statistical tests, that only test whether one or the other model is a better fit, not whether a mixture of both is even better. Ultimately, it is most likely that humans are, to some degree, capable of both types of cognition/decision making and what model dominates is dependent on person, circumstances and decision task at hand. To be fair, I don't think the authors are truly proposing the absolutist position that comparison processes cannot exist, but the framing of the paper as a complete either or is maybe a bit strong.

2) One of the authors key findings seems to be that the overall value of the two options (they call it richness) appears to have a specific impact on decisions, namely that the richness of an environment drives switches, not the relative value between the options. However, participants could have an option specific comparison mechanism but in part infer the value of the alternative using the environment richness, instead of only relying on option specific reward histories. In short, if I received a lot of negative feedback recently, I would also be more willing to switch between all available options to look for evidence of one option being good, as there being a good option in the set somewhere is a reasonable assumption to make. This relates to point 1 as it suggests that an adapted option specific model could predict the environmental richness related effects without giving up the notion of option specific comparisons altogether.

3) I was very confused by why the authors thought exp 2 was favouring an option comparison algorithm. Surely, if you want to design a task to favour option specific representations you'd give explicit counterfactual feedback instead of linking the options outcomes with each other. By linking the options, a threshold-based mechanism can be a perfect way to do the task as there is a threshold at which the alternative automatically has to be better. Additionally, there should be many previously published datasets available with counterfactual feedback, where they can test their hypothesis and directly pit two known and concurrently tracked option value models with switch stay comparisons that don't use the value difference, as this would be a nice and useful test of their theory.

4) While I very much like their results of Choice Run and HMM results, I feel like very similar data could be obtained with a stickiness model/ choice perseveration kernel. Fundamentally, the problem is that the currently state of the art models of option comparison would be perfectly fine with adding choice perseveration (as they have added in some comparisons

between model classes and which reduced the difference in model fit considerably (see F3J+K). The question is whether addition of some type of normalization and/or inference using the richness of the currently experienced environment to change the value expectation about alternatives, together with stickiness would be sufficient to explain their data completely. The difference between such a model from theirs would be that fundamentally comparisons would still be based on option specific representations, not option indifferent stay-switch comparisons. Ultimately, the best test of their theory would be whether in a multi-option data set, participants retain the object specific value representations after repeated switches, and therefore attach the value to specific options, not simply switch stay behaviours (which could be done easily with a task with multiple option values and counterfactual feedback as suggested in point 3). Additional tests could manipulate the value of the environment independently to see what impact that has on switching behaviour (if any) and whether these effects are better explained through normalization mechanisms based on environmental richness or an explicit switch vs stay decision explanation. However, I suspect that they'd find a mixture of behavioural drives, i.e. a limited retention of option specific reward information (likely based on working memory) and a tendency to stick with previous options, particularly if they appear good enough (in fact, satisficing has been a very empirically useful concept in ecological behavioural modelling, psychology and economics).

5) I didn't understand why the authors did not use simple logistic GLM approaches throughout the manuscript? Specifically, I don't understand the whole point of the strategic finger print fitting of the simulated binned data. I might be mistaken but the data can be understood and analyzed perfectly well with a regression approach in which you test for the richness (Value A+B) and value discriminability/Value difference (ValueA-B) in GLMs. If you then predict the y=switch, you can simply test the impact of richness on switching, while linearly controlling for value difference. If you want you can even throw other additional terms and shapes in, if you are concerned about non-linearities. So far, I don't understand the additional value of a PCA derived 7 dimensional fingerprint instead of going with commonly accepted, appropriate and more easily interpretable logistic regressions, particular given the nature of their data. With such an approach you can simply test the intercept of the GLM (or if you want to argue bias you can fit your own softmax equivalence point) to measure staying bias and the impact of richness on switching behaviours to proof sensitivity of environmental context on switching. However this would probably show that there is an additional impact of RL derived option value difference on switching, which would suggest a mixture of effects. If they see literally no impact of value difference in such a GLM (in a paradigm in which the option values fluctuate independently, meaningfully and beknownst to the participants), I would consider they have evidence of a lack of option comparison in their data.

6) In the same vein of unusual behavioural modelling, I didn't understand why the authors use a delta reward rule for switching for the simulations rather than just simply using a softmax on the value difference with a fitted or different simulated biases terms? If I understood their procedures simulation correctly it is almost what they are doing, except that they are using unsigned, rather than signed difference, but it would be way easier to understand if they just described it as a switch-stay bias term of a softmax.

7) The discriminability richness relationship (shown in figure 2) is not a necessity of decision making, but more an artefact of the task design. I am not sure why they did not collect at least one data set without those constraints to prove their points?

8) I am also a bit puzzled why they sometimes write "RL vs Foraging" as the "Foraging" model also uses an RL algorithm to update value. the option based choice vs environment comparison vs switch seems more valid.

Minor comments:

A) It would be very helpful to have a better breakdown of the existing 3 experiments at the start. Specifically, what are the exact tasks and what is manipulated between the experiments and for what purpose. As far as I understood it, Experiment 1 is a simple two option Bandits tasks, experiment 2 has linked probability options and experiment 3 has more options and volatility/uncertainty manipulations?

B) Actually, switching a lot at low discriminability levels, isn't necessary utility optimizing as both options are similar in value and therefore the marginal value of picking the better one is low, particularly if one assumes as cost to deliberation, which is also hardest with low value differences.

C) For the first experiment switching to “random” is same as switching to the only other option, no? If so that should be clearer.

D) Similar to A, but if the authors don't want to change the overall structure of how the present the experiments (which I leave up to them), they should at the very least improve how the illustrate the Exp 2 and 3 to make it clearer what they look like and what is being manipulated schedule wise.

(Remarks on code availability)

Reviewer #2

(Remarks to the Author)
In this paper, Zid and colleagues investigate whether human sequential choices under uncertainty with partial information (i.e., only about the selected option, creating an explore-exploit tradeoff) are more similar to computations that compare option values to make a choice ("compare-alternatives") or computations that compare only the value of a currently selected option to a fixed switching threshold ("compare-to-threshold"). They report evidence that compare-to-threshold models better capture subjects' choices in non-stationary (drifting) 2- and 3-armed bandits, and provide a better fit to detailed aspects of the temporal dynamics of subjects' repetition and switching. The authors interpret these results as indicative of "foraging computations" and suggest that this has broader implications for models of decision making under uncertainty.
We broadly agree with the aims and importance of the article. Indeed, there are long-standing puzzles about the nature and causes of human decision-making biases in general, and biases in reward-based sequential decision tasks in particular, relative to standard (compare-alternatives) formulations of utility-theoretic models. Despite much research on such biases, and despite their likely theoretical importance, conclusive answers remain incomplete, and the direction taken by the authors is a promising one.
Overall, we find the study's methodology to be sound and rigorous, and the paper to be clear and well argued. To name a few aspects of the study that we appreciate: first, the framing of decisions in bandit problems in terms of foraging theory is, to our knowledge, a rather novel contribution that helps relate foraging frameworks to a large body of work in psychology and neuroscience. Second, the finding that compare-to-threshold models perform worse on task than compare-alternatives, but provide a better fit to subjects' choices, is indeed intriguing and suggestive. Third, the model comparison and the sequence of model extensions are mostly convincing. Finally, the detailed analysis of repetition patterns in terms of multiple decay rates of choice history effects and relating these to latent explore-exploit states helps to make sense of why the compare-to-threshold models provide a better fit to subjects and situates the study as a natural and important extension of the lab's previous work.

Nevertheless, we find important but remediable shortcomings in the currently reported results, which are currently inconclusive with respect to the main research questions and limit the impact of the paper. We also believe that the framing of the study can be sharpened to clarify the paper's contributions. We summarize our main criticisms and suggestions as follows.

[1] It appears that some very direct tests for potential rejection of the compare-to-threshold model are not included. A core assumption of this model is that the probability to repeat/switch is not sensitive to the value of the other (=switch) option(s), but only to the repeat option (which is compared to some threshold).
A very direct test of this assumption in the 2-armed bandit case would be to run a logistic regression of choices, with the decision to repeat as the dependent variable and as regressors a constant, the value of the switch option, and the repeat option. The compare-to-threshold model predicts no effect of the switch option value, while the compare-alternative model predicts a negative effect. Note that there are several ways to perform this analysis: using the generative reward probabilities for the option value, or using model-based estimation, e.g., with a delta-rule model, with an ideal observer model, etc.
Another very direct test of this assumption in the 3-armed bandit case is that when a switch occurs, the choice probability should be uniform among the other two alternatives. In contrast, the compare-alternatives models predict that this probability should reflect the reward probability of those alternatives. The authors conducted a 3-armed bandit task in the experiment, which is a strength of the paper, so testing this prediction is only a matter of additional analysis.

[2] To rule out the compare-alternatives model requires ruling out the entire class of RL models. This is not easy because it requires testing many variants of the RL model. The authors made the commendable effort to try several alternatives. We are not aware of any method that would guarantee that all possible variants have been tested. We make the more modest suggestion that two more variants be included.
(i) The models appear to differ in their ability to capture subjects' repetition behavior. A simple extension of the RL model (and possibly the food model) is to include a repetition bonus in the softmax. In the RL model, this would correspond to a dummy regressor indicating whether option a_i was selected in the previous trial (in the case of the 2-armed bandit, it is also possible to reparametrize the model into choice repetition versus switch and include a constant). The history kernels come close, but in more sophisticated forms that do not capture the simplest form of repetition bias.
(ii) In the compare-to-threshold model, the threshold can be interpreted as a prior about the richness of the environment. Such a prior is not included in the RL model, but it is possible to do so, see for example the work of Angela Yu. In her learning model, the estimation of the option value includes a prior about the expected value of options in general (thus capturing an optimism/pessimism bias of the participant). This prior is persistent and recurring in the value update, is different from the initial value of the delta rule, and actually requires extending the delta rule into a slightly more complex learning model.

[3] There are problems with model comparison:
(i) The baseline model for compare-to-threshold has 3 free parameters (threshold, softmax temperature, and learning rate), and the compare-alternatives model has 2 free parameters (softmax temperature and learning rate). It is generally misleading to report log-likelihood as the primary metric for models that have different numbers of free parameters (e.g., reported in the first place, l. 170; in Fig. 5; used for the foraging index). Consider replacing it with a cross-validated estimate of the log-likelihood, or reporting it as a secondary metric after a metric that accounts for the difference in the number of parameters (e.g., AIC, as used in the paper).
(ii) The comparison at the group level is based on a fixed effect. Random effects analysis for subjects in a group is more powerful (see e.g. Stephan et al; note that it is not the only methods for random effects analysis, we do not ask the authors to use this one in particular, although it would address our point).
(iii) For the individual-level model comparison (l. 173-177), the metric used seems to be the log-likelihood rather than AIC, which is not appropriate in this case given the difference in model complexity. It may be a typo, with log-likelihood instead of

AIC, since the reported value is positive (which is impossible for log-likelihood).

[4] How is a (stationary) threshold learned or calibrated in a particular decision context? Although the authors note that thresholds are assumed to be learned over long periods of time, they cannot be invariant to aspects of the decision context if compare-to-threshold is to be a viable model of actual animal decision making. Again, this may be out of scope, but the current wording of the paper seems overly simplistic: it would be helpful if the discussion commented on how such thresholds might actually be set.

[5] Isn't the most plausible model a hybrid between compare-alternatives and compare-to-threshold? (and possibly other strategies). It is known that people can of course explicitly compare alternatives, and it would be quite extraordinary if they never or rarely do so in reward-guided sequential decision tasks under uncertainty. The space of latent choice-generating states plausibly includes threshold-comparison and alternative-comparison states (e.g., as a further hierarchical level). For example, in the present study, there may be stronger evidence for compare-alternatives early in the task (when a threshold may not have stabilized), which is gradually overtaken by compare-to-threshold, which in turn may give way to compare-alternatives in instances of apparently large changes in the environment. Including this would be an important contribution, in our view, because it would (a) be more faithful to the rich structure of actual decision making, where a number of non-mutually exclusive mechanisms are likely to be at play, and (b) be informative for algorithmic accounts of how compare-to-threshold might be implemented. It may also point to [4] above as a potential mechanism for calibrating switching thresholds to particular decision contexts. If the authors feel this is out of scope, it seems worth mentioning in the discussion.

[6] The paper should acknowledge that the conclusions are drawn from a specific class of problems: k-arm bandit with drifting reward probabilities and binary observations. This makes the current observation, as well as past observations, less informative about the current latent option value than in bandit problems with fixed or infrequently abruptly changing values, and bandit problems with continuously valued observations. This may make the difference between RL and compare-to-threshold smaller than in other tasks. In addition, the data were collected on Amazon MTurk, where data quality is known to be often limited (although the average performance of the sample analyzed is reassuring). Acknowledging these two points seems useful.

Minor:
- it seems that the win-stay-loose-shift (in a probabilistic version that chooses randomly with a probability epsilon) would be a useful baseline to include in the paper. It is mentioned briefly at the beginning of the paper as a very good, simple model of the data; it could be included more exhaustively in all figures and analysis, being a simpler model than RL and forage.
- please show the model predictive checks for Fig 2D (i.e. corresponding values from the fitted models)
- the delta rule model for V-exploit seems not completely consistent. In particular, the model reinforces the exploit decision when the agent is rewarded for switching. I understand why it is the case, but it may be more intuitive to present the model as increasing the probability to repeat whenever the current choice is rewarded, with an intercept that corresponds to the (negative) threshold rho.
- l. 28: rath(er: aberrant bracket
- l.107: Fig 2A shows that when discriminability is low, richness can only be very low or very high. Fig 2A shows that it can be any level of richness. I think what the authors want to say is the other way around: when richness is very high/low, discriminability can only be low.
- l. 233: moreso: more so
- l. 391: option to to try: delete to
- l. 395: an more: a more

References
Guo, D, Yu, AJ (2018). Why so gloomy? A Bayesian explanation of human pessimism bias in the multi-armed bandit task. Advances in Neural Information Processing Systems, 32.

Ryali, C, Gautaum, R, Yu, AJ (2018). Demystifying excessively volatile human learning: A Bayesian persistent prior and a neural approximation. Advances in Neural Information Processing Systems, 32.

Stephan et alKE, Penny WD, Daunizeau J, Moran RJ, Friston KJ. Bayesian model selection for group studies. Neuroimage. 2009 Jul 15;46(4):1004–17

(Remarks on code availability)

Reviewer #3

(Remarks to the Author)
I co-reviewed this manuscript with one of the reviewers who provided the listed reports. This is part of the Nature Communications initiative to facilitate training in peer review and to provide appropriate recognition for Early Career Researchers who co-review manuscripts.

(Remarks on code availability)

Version 1:

Reviewer comments:

Reviewer #1

(Remarks to the Author)
My comments the authors replies below. In short, I don't think there is much left for the authors to address, except some very minor textual changes, if at all. Congratulations and a great study!

1) Good
2) I didn't mean to imply that the model explicitly calculates richness, just that the richness of the of environment participants experience, which is correlated with the value of both options, impacts decisions, even if the model doesn't calculate richness itself, but only switches when experience falls under a certain level. What I am saying is that even if you generally do option based learning, having option independent repeated negative reward experiences could make you more exploratory, which is another way of saying that people might have a mixture of option specific learning and "foraging" based exploratory drive when things e.g. get worse. I however, get your point, that I hadn't fully appreciated before, that I should have more carefully dissociated between the environmental richness as the average value of both option which will reduce exploration as both options are "good enough" and the other concept of comparison between experienced richness i.e. value of past choices independent of option, which the model does, unless I am mistaken, track explicitly to compare to their threshold. In short, I agree that the model doesn't really do average value of both options directly, but could be impacted by it (and behaviour seems to be, which is interesting) but the foraging model is directly linked to an exploratory urge when reward experiences go down, which could be seen as an additional drive to explore on top of option specific drives. However, as the authors point out, if this is true, it seems that for their tasks, the reward experience based drive outweighs the option specific one, on average.
3) I am a bit surprised that the authors couldn't find an openly available dataset, but belief them that they tried. I naively assumed there was more of an commonly shared resource than there appears to be, particularly for such canonical tasks as 2 armed bandits.

4) The authors state "Briefly, the majority of participants did not retain alternative option values and those who did tended to chose the worst alternative–not the best. This is difficult to reconcile with a traditional RL model, although perhaps a good compare- alternatives model could be engineered that is capable of reversing its representation of values from time". This is indeed a very cool finding and contradicts RL models. I find the authors explanation that participants might have a vague sense of not having tried an option for a long time and that being correlated with an option being historical bad, believable for explaining why people actively prefer the worse option, but either way, it does serve a very important piece of information against option specific mechanisms!
5) I agree with the authors on my misunderstanding of the direction of the unchosen option effect and that it shouldn't play a role! They are correct in pointing out that only an environmental richness mechanism would change their switching based on the value of the unchosen option and only if it maintain such a memory, which the foraging model doesn't. It's nice to see the prediction of the foraging model, a chosen option dominance, come out in a conventional GLM analysis.
6) –
7) I understand that probability space is highly constrained and bounded but disagree that most reward environments tie discriminability and richness together just because our experiments often do. In the real world we experience many environments with differing reward richness independently of the difference in value between options. It just happens to be the case that most researchers are tying themselves to reward probability for learning tasks instead of magnitudes but in magnitude space dissociations between differences and means/richness are very doable even without raising ethical concerns. However, in theirs and many other tasks they are correct about the relationship.
8) Good

Minor points are also all addressed.

(Remarks on code availability)

Reviewer #2

(Remarks to the Author)
We appreciate the authors' thorough revisions and careful consideration of our comments. We find that the additional analyses and revised manuscript address most of our initial concerns. However we do have two main outstanding concerns.

[1] First, in addressing our comment [1], the authors use the objective (generative) values of the chosen and unchosen options, in both the 2- and 3-armed bandit analyses. This seems like an unfair test of the potential use of the unchosen option's (subjective) value in subjects' decisions because of an asymmetry in how closely correlated the objective and

subjective values would be for the chosen versus the unchosen option. Specifically, the objective and subjective values would be more strongly correlated for the chosen option and less so for the unchosen one, precisely because the unchosen option is not sampled much when subjects often repeat their choices. This biases the analysis against finding a role for the unchosen option's value in decisions (and in favor of the compare-to-threshold model).

As the authors note, it is not clear which model's subjective value should be used, given the many model variants reported. A good possibility, we suggest, for a subjective value estimate can be derived from an ideal Bayesian learner, where the options' value reflects the sampling history. Note that this model advantageously has no free parameter, it is not fit to participant's behavior. There is no truly 'neutral', atheoretical version of this subjective value. The Bayesian value is conservative insofar as compare-alternatives models provide a closer approximation to an optimal learner, and is hence a strong test of the extent to which subjects may track the unchosen value. If it diverges strongly from the results with generative values currently used, perhaps it is most fair to report both results and discuss this point.

[2] Second, in their response to our comment [2], the authors implement a version of the dynamic belief updating model that does not allow for an optimism or pessimism bias because P0 is kept fixed. This value should be modeled as a free parameter to capture a persistent prior (where the value of this persistent prior is fitted from choices, just as the threshold in compare-to-threshold is fitted to choices). Therefore, this analysis provided in the response does not currently rule out that (some) subjects may have a persistent prior rather than that they are comparing to threshold.

Lastly, in our point [3] concerning model comparison, we recommended that the authors use cross-validation rather than BIC / AIC to avoid assumptions about the appropriate size of the penalty given correlated decision factors. Although we feel we should accept the authors' decision to stick with information criteria because these metrics are so widely used, our point is even more salient now that the authors use both BIC and AIC in different analyses. Specifically, they switch to BIC in response to point [1]: "Model comparison was evaluated with the Bayesian Information Criterion because we found that the Akaike Information Criterion had an insufficient penalty for the number of parameters (i.e., the full model was generally preferred even when option values were completely randomized)" (p. 25). It would be strongly preferable to use a consistent metric for model fit improvements throughout the paper. We once again urge the authors to consider using cross-validation in comparing fit improvements.

Very minor:
This is probably a typo, but the sample sizes vary in the reported analyses on p. 25 (i.e., 254 in some places, 258 in others).

(Remarks on code availability)
I had a superficial look at the code base. It seems that:
- the revisions are not part of the github repo linked to the submission (last commit 10 months ago)
- for the original version, the content is partial and it covers only experiment 1
So it seems that the current repo does not make it possible to reproduce the results of the paper (exhaustively). Maybe the authors can clarify whether the intention is to provide a partial demo, or to release the entire code base in the future.

Reviewer #3

(Remarks to the Author)
I co-reviewed this manuscript with one of the reviewers who provided the listed reports. This is part of the Nature Communications initiative to facilitate training in peer review and to provide appropriate recognition for Early Career Researchers who co-review manuscripts.

(Remarks on code availability)
Only had a superficial look together with my co-reviewer. Please refer to his note.

Version 2:

Reviewer comments:

Reviewer #1

(Remarks to the Author)
The authors have addressed all my remaining comments.

(Remarks on code availability)

Reviewer #2

(Remarks to the Author)
In their revision, the authors adequately updated the models used in the paper. This extensive model comparison makes the claim of the paper more convincing.
I believe there is an error in Fig S4E, as the best model seems to now be the traditional RL model. Some labels for RL model

and foraging model are probably swapped by mistake.
Regarding the metric used for model comparison, we let the final decision to the authors and editors. We simply note that, from the reader's perspective, having different metrics in the same paper raises the concern of post-hoc selection. An easy fix would be to remove the only BIC value reported in the paper, and replace it with AIC if that's the chosen metric.
Regarding our suggestion to use cross-validated likelihood, it is because unlike BIC and AIC, this method does not make assumption about how to penalize the model based on a number of free parameters, it is thus more general.

(Remarks on code availability)
The code base remains largely incomplete, although it has been updated recently. In particular I don't find the analyses for Experiment 2 (if it's there, it should be better documented in the readme) and several files and paths are hard coded on the author's computer and not available (e.g. I could not find volatilityLMH_newstates2025.mat that is required for unchosenOption_exploreStrategy.m)

Reviewer #3

(Remarks to the Author)
I co-reviewed this manuscript with one of the reviewers who provided the listed reports. This is part of the Nature Communications initiative to facilitate training in peer review and to provide appropriate recognition for Early Career Researchers who co-review manuscripts.

(Remarks on code availability)

Open Access This Peer Review File is licensed under a Creative Commons Attribution 4.0 International License, which permits use, sharing, adaptation, distribution and reproduction in any medium or format, as long as you give appropriate credit to the original author(s) and the source, provide a link to the Creative Commons license, and indicate if changes were made.
In cases where reviewers are anonymous, credit should be given to 'Anonymous Referee' and the source.
The images or other third party material in this Peer Review File are included in the article's Creative Commons license, unless indicated otherwise in a credit line to the material. If material is not included in the article's Creative Commons license and your intended use is not permitted by statutory regulation or exceeds the permitted use, you will need to obtain permission directly from the copyright holder.
To view a copy of this license, visit https://creativecommons.org/licenses/by/4.0/

In this document, we have included all the reviewers' comments in **bold**, our responses in black, and quotes from the revised manuscript in blue. In the manuscript all changes are visible in blue with previous language in ~~red~~.

1 Reviewer #1

This manuscript is about modelling classical drifting bandit tasks, not as a comparison between independent, learned value estimates, but instead a single variable that tracks an option unspecific recency weighted reward history to determine switching based on a threshold comparison with the current reward estimate of staying. Overall, this is very interesting idea that seems to have been in the air for some time and boils down to people having a sense of what reward level constitutes a "good" option and not exploring unless the option falls below a certain level (strictly speaking the average reward rates, but as participants have long stretches of exploitation it often boils down to the same thing). I liked the idea to use tasks that favour option comparison mindset (or are at least primarily modelled like that) to test their switch vs stay decision making models, but the current set of experiments and weight of evidence, do not, at this point, fully support their very strong claims of a lack of option based comparisons in favour of foraging / switch vs stay decision making, both due to the problematic nature of either/or statements from the data and because only a limited set of behaviours were tested. Below I have listed my specific concerns point by point (grouped into minor and major):

We appreciate the reviewer's thoughtful and detailed reading of our manuscript and kind words about the presentation of our data and the computational framework we used. The reviewer also raised several important issues, particularly surrounding the framing of the manuscript, and rationale for certain analyses. To address these comments, we have clarified the framing, better detailed our rationale, better described certain analyses, and added multiple new results.

These new analyses include (1) a choice predictive Generalized linear model (GLM) on behavior from Experiment 1, (2) analysis of alternative option value on switching behavior from Experiment 3, (3) fitting both traditional RL and foraging-RL to new 2 new Experiments, (4) developing 2 novel extensions to both traditional RL and foraging-RL to explore how integrating information about richness and choice history would influence model fit and (5) fitting these 4 models to behavior from Experiment 1. We have also added several new figures, including (1) Figure 1F-G, (2) Figure 2A-B/G-H, (3) Supplementary Figure S1, (4) Figure 3J-K, (5) Supplementary Figure S3, (6) much of Figure 5, and (6) new Supplementary Figure S5). We believe these changes have significantly improved the manuscript and we hope have satisfied the important concerns raised by the reviewer.

1) Fundamentally, the notion that humans either ONLY do option specific comparisons or foraging like switch stay decisions is unlikely to be true. Furthermore, this conclusion is not supported by the statistical tests, that only test whether one or the other model is a better fit, not whether a mixture of both is even better. Ultimately, it is most likely that humans are, to some degree, capable of both types of cognition/decision making and what model dominates is dependent on person, circumstances and decision task at hand. To be fair, I don't think the authors are truly proposing the absolutist position that comparison processes cannot exist, but the framing of the paper as a complete either or is maybe a bit strong.

Although the revised manuscript now provides stronger evidence that people, on average, tended to use compare-to-threshold computations in these tasks, we have revised the manuscript to highlight the small proportion of individuals who did not and the potential for a diversity of solutions across people. We have made the following changes:

In the introduction : Together, our findings suggest that humans are more likely to use compare-to-threshold computations than previously understood: across multiple variations of a task commonly used as a testbeds for compare-alternatives computations, people tended, on average, to use foraging-like algorithms.

In the discussion: Although human and animal decisions in sequential decision-making tasks are typically modeled as a compare-alternatives process [9, 30, 32, 10, 18, 31, 2, 16, 8], this is an assumption has not been rigorously tested. Here, in a restless, probabilistic k-armed bandit task, we found that human behavior is, on average, better explained by compare-to-threshold computations. (...)

Although the majority of participants' choices were better described as a compare-to-threshold process, we did find evidence of compare-alternatives computations in a small minority of participants (1.2%, 4.3%, 2.2% or 7.8%, depending on the analysis). **We cannot rule out the possibility that this proportion would be higher in different tasks or in unexplored variants of this task (e.g., change point tasks, static bandits, bandit problems where reward magnitude drifts rather than probability, or bandit problems without feedback noise).** Because the compare-to-threshold strategy is both computationally simpler and less rewarding than the compare-alternative strategy, it seems plausible that different results might be obtained if the stakes were higher and/or the cognitive demands lower. Indeed, when we raised the demands, increasing the number of options and he dimensionality of the task, the advantage of the foraging-RL model also grew—an intriguing parallel to the computational advantage that could come from adopting a strategy that need track only 1 value in increasingly high-dimensional environments. Of course, future within-subjects work is needed to determine if this result reflects a change in strategy across individuals or just some improved ability to discriminate the models in higher-dimensional environments. One manipulation that would be especially important in future studies is full outcome feedback (where the outcome of both chosen and unchosen options are explicitly provided, e.g., [16, 31]. Full feedback might encourage compare-alternatives strategies [16], though it would be important to differentiate true compare-alternatives computations (where option values are tracked independently over time) from a compare-to-threshold strategy that operates on differenced values (i.e., where relative value is calculated at the time of encoding, rather than at selection). Of course, we do not mean to exclude the possibility that humans use a repertoire of strategies, switching between them as needed. For example, in the 3-alternative task, a substantive proportion of participants (16%) did not explore alternative options uniformly, though roughly equal proportions of these were biased towards the best alternative (consistent with using a compare-alternatives strategy for exploration) or towards the worst (consistent with an as-yet-unidentified exploratory strategy). While more work is clearly needed, these results do suggest that compare-to-threshold models should be evaluated even in tasks that are conventionally modeled using compare-alternatives computations.

2) One of the authors key findings seems to be that the overall value of the two options (they call it richness) appears to have a specific impact on decisions, namely that the richness of an environment drives switches, not the relative value between the options. However, participants could have an option specific comparison mechanism, instead of only relying on option specific reward histories. In short, if I received a lot of negative feedback recently, I would also be more willing to switch between all available options to look for evidence of one option being good, as there being a good option in the set somewhere is a reasonable assumption to make. This relates to point 1 as it suggests that an adapted option specific model could predict the environmental richness related effects without giving up the notion of option specific comparisons altogether.

This appears to be related to a misunderstanding of our richness analyses that we have labored to clarify in the revised manuscript. Briefly (and we will return to this point later), the foraging model does not calculate richness. It only depends on richness as a byproduct of the fact that any option is more likely to be higher than a threshold in a rich environment. That said, this comment made us curious about what would happen if both the traditional RL and foraging-RL models *did* incorporate information about richness. In short, while we find that richness is indeed a useful variable to track in the environment, tracking richness is not the essential feature that makes the foraging-RL model a better model than the traditional RL model.

In order to test this idea, we asked whether including an estimate of the environment's richness could improve model fit, implementing it along the lines suggested by the reviewer. Specifically, while there are many ways to formally add richness information to both the traditional RL and foraging-RL models, we chose an approach that 1) uses richness to scale exploratory noise, 2) explicitly computes richness in an option-agnostic way, and 3) is compatible with both models. To summarize the results, fitting these new models revealed that including richness does indeed improve the traditional RL model (i.e., AIC is lower for traditional RL richness, Fig. 3J). Crucially, the extension of the foraging-RL algorithm also benefits from this richness information, though to a lesser extent. Again, foraging-RL is better than

traditional RL, consistent with our previous observations that foraging-RL models are better fit as a class to participants' choices than traditional RL. We have added a description of these extensions to the methods, added these models to Figure 3J-K (i.e., richness), and added the the full results in Sec. "Foraging-RL models as a class better predict participant decisions". We have also added how richness traditional RL compares to standard traditional RL for one example participants in supplementary Figure S3A-D We believe these changes help clarify the relationship of each model to environmental richness. Revisions are as follows:

Methods:

Richness One of the major differences between compare-alternatives and compare-to-threshold strategies was that only the former linearly depends on the richness of the environment, paralleling the pattern seen in humans (Figure 2D-F). Although it does so only as a byproduct of the fact that any random option value is more likely to be above some threshold in a rich environment, it remained possible that explicitly incorporating information about richness into either the traditional RL or foraging-RL models might improve model fit. It makes intuitive sense that using richness to re-normalize values—scaling decision-noise down when options are generally good, and up when they are bad—could be a useful way to both improve overall performance and generate the kind of results seen in Figure 2F. Therefore, we next added richness-dependent noise to both the traditional and foraging-RL models. In both foraging-RL and traditional RL models, richness was tracked through a variable μ_t, that was updated through an exponential filter:

$$\mu_{t+1} = \mu_t + \alpha_\mu (r_t - \mu_t). \tag{1}$$

where r_t is the reward received at trial t, independently of which option was chosen, and α_μ is the learning rate for richness, fitted to each participant. This means that μ is a choice-independent, subjective estimate of richness that depends on the actual rewards received across all options. This richness μ is then used to adapt the softmax decision rule by scaling the value v through the richness,

$$\text{in traditional RL,} \quad p(\text{choice}_t = i) \rightarrow \frac{\exp((\beta + \beta_\mu \mu) v_{i,t})}{\sum_{j=1}^{N} \exp((\beta + \beta_\mu \mu) v_{j,t})}$$

$$\text{in foraging-RL} \quad p(\text{exploit}) \rightarrow \frac{\exp((\beta + \beta_\mu \mu) v)}{1 - \exp(-\beta(v - \rho) - \beta_\mu \mu v)},$$

where $\beta_\mu \geq 0$ is an inverse temperature for the specific contribution of the richness. This extension adds two additional free parameters: $\alpha_\mu \in [0, 1]$ and $\beta_\mu \in [0, 100]$. We can see from the equations that this approach remaps the inverse temperature of the standard model, $\beta \rightarrow \beta + \beta_\mu \mu$. This says that decision noise is adapting to the experienced richness. If richness is high in either model (i.e., close to 1), and $\beta_\mu \neq 0$, then the overall inverse temperature increases, choice curves sharpen, and the probability of choosing the highest value option goes up. In contrast, if richness is low (i.e., close to 0), the overall inverse temperature decreases, choice curves flatten, and exploratory noise increases. Because the learning rates for option value (or exploitation value) and richness can be different, this model is flexible in how it adapts to experienced richness.

Results: A variety of extensions and alternatives are known to outperform the standard 2-parameter traditional RL model, and it remained unclear whether any of these would fit behavior better than the foraging-RL model. Therefore, we next compared the foraging-RL model with 10 additional models: 4 common extensions of the traditional RL model (asymmetrical learning, value decay, and 2 forms of choice history dependence), 2 dynamic Bayesian models and a free-fitting exponential filter (see Supplementary Figure S3E), and **3 novel extensions meant to address limitations of the traditional RL model identified here (see Methods)**. (...) **Foraging-RL also outperformed 3 extensions to RL models that were developed to account for our behavioral observations: an RL model in which values are explicitly normalized by richness (see Methods; "richness"; Supplementary Figure S3E; Supplementary Table 1)**, a repetition bonus model that implements a tradeoff between repeating the last option and making decisions based on relative value (see Methods; "repetition bonus"; Supplementary Figure S3E; Supplementary Table 1), and a model that combined richness normalization with the best formulation of choice-history dependence (see Methods; "richness + choice + 2"; Supplementary Figure S3E; Supplementary Table 1). (...)

Critically, any one traditional RL model could have performed better than the standard foraging-RL model for two reasons: either because it benefits from the compare-alternatives architecture or because decades of incremental engineering were ultimately able to overcome some of the limitations of that architecture. Therefore, we next compared foraging-RL models with traditional RL models as a class. **We developed an equivalent version of the foraging-RL model for each extension of the traditional RL model** (see Methods; Figure 3J-K; Supplementary Figure S3E). Note that our goal here was not to build the best foraging-RL model we could, but instead offer a fair compare-to-threshold version of each common variant of the traditional RL model. **We found that every extended foraging-RL model outperformed the equivalent traditional RL model at the group level** (Figure 3J-K, S3; Supplementary Table 2), suggesting that compare-to-threshold models outperformed compare-alternatives models as a class. (...))

	**model**	**total parameters (k)**	**AIC**	**log-likelihood**	AIC_w
**traditional RL**	repetition bonus	762	48323.8	-23,399.9	< 0.0001
	richness	1016	48,913.3	-23,440.7	< 0.0001
	richness and history kernel 2	1524	48,787.7	-22,869.8	< 0.0001
**dynamic belief**	exp-DBM	508	53,150	-26,067	< 0.0001
	DBM	508	51,025.9	-25,004	< 0.0001
	exp	1016	50,873	-24,420	< 0.0001

Table 1: **Pairwise, group-level model comparison against standard foraging-RL for Experiment 1**. AIC weights are computed relative to standard foraging-RL (AIC_w for standard foraging-RL was 1 in all pairwise comparisons).

**extension**	**model**	**total parameters (k)**	**AIC**	**log-likelihood**	AIC_w
**asymmetrical**	foraging-RL	1016	47,585	-22,777	1
	traditional RL	762	49,450	-23,963	< 0.0001
**decaying**	foraging-RL	1016	47,295	-22,632	1
	traditional RL	762	47,887	-23,181	< 0.0001
**history kernel 1**	foraging-RL	1016	47,148	-22,558	1
	traditional RL	762	48,028	-23,252	< 0.0001
**history kernel 2**	foraging-RL	1270	47,125	-22,292	1
	traditional RL	1016	47,351	-22,660	< 0.0001
**richness**	foraging-RL	1270	47,419.4	-22,439.7	1
	traditional RL	1016	48,913.3	-23,440.7	< 0.0001
**richness history kernel 2**	foraging-RL	1778	46,904.3	-21,674.2	1
	traditional RL	1524	48,787.7	-22,869.8	< 0.0001

Table 2: **Group-level class comparison for Experiment 1.** AIC weights are computed relative to the equivalent foraging-RL model.

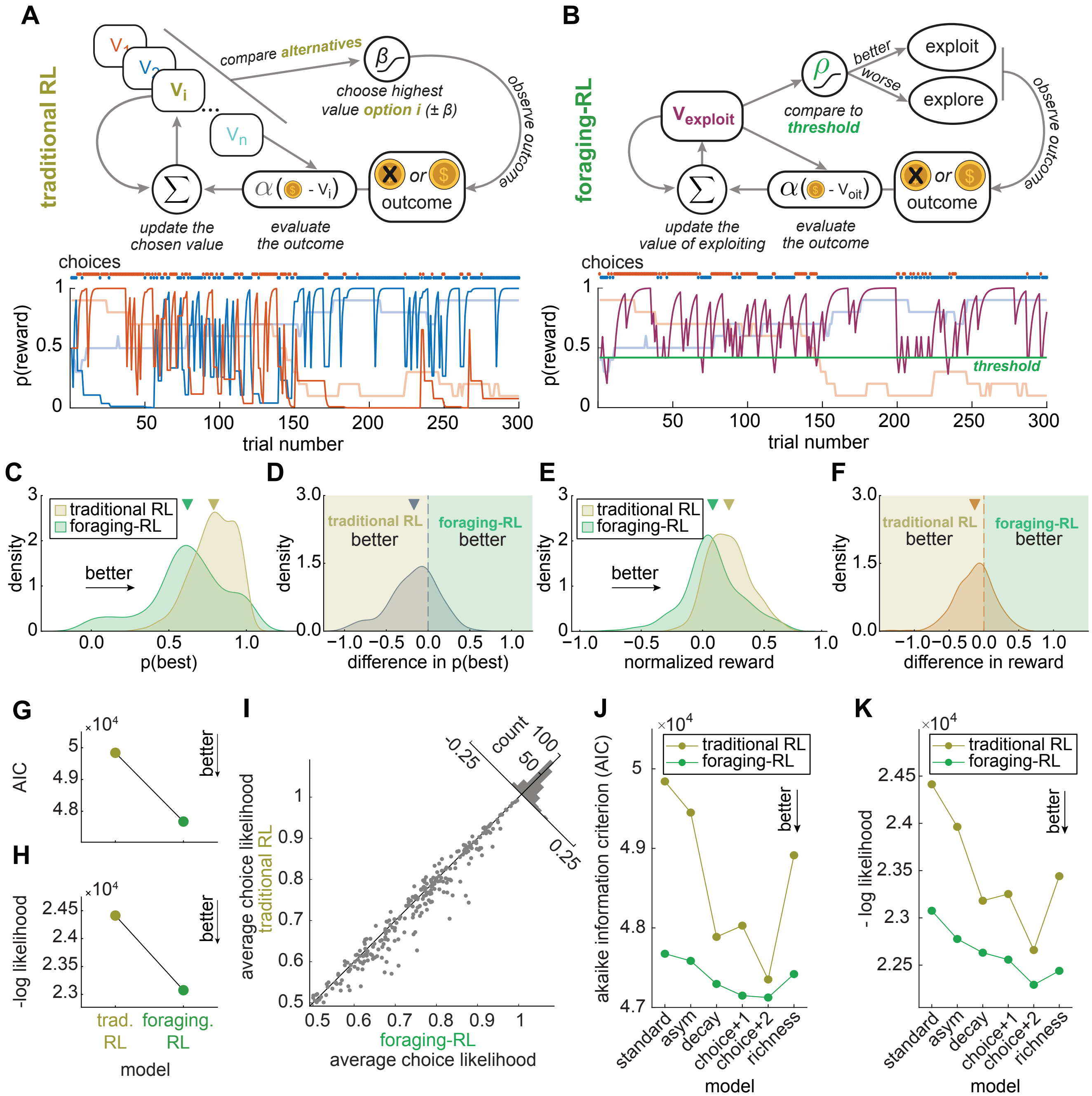

Figure 3: **Traditional RL and foraging-RL models produce different choice patterns, performance and quality fits to the data.** **J)** The Akaike information criteria of the extensions of traditional RL (*gold*) and foraging-RL models (*green*). **K)** The negative log-likelihood of the extensions of the traditional RL (*gold*) and foraging-RL models (*green*)

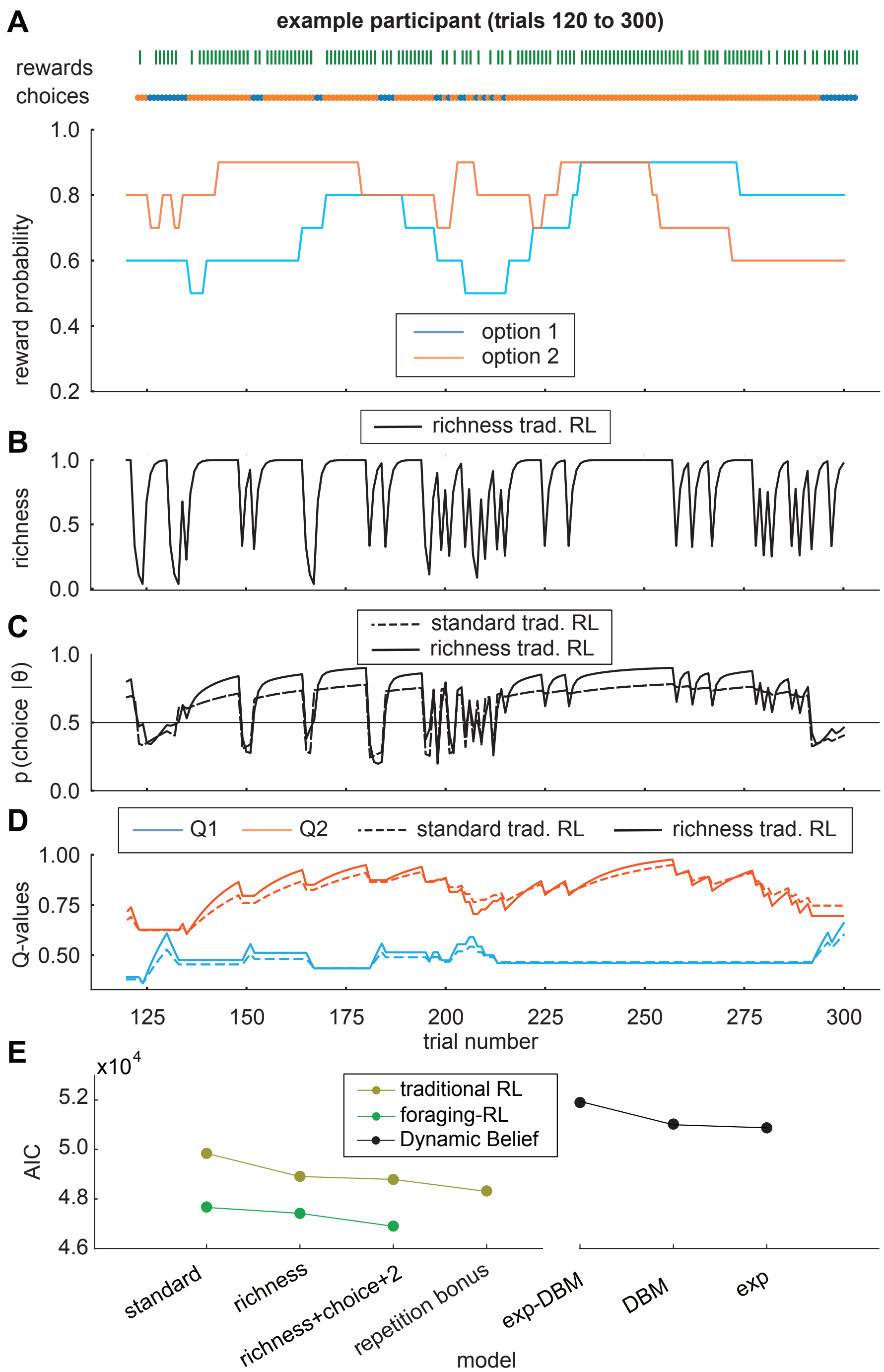

Figure S3: **Additional model fits. A)** Example reward probabilities from a single participant in Experiment 1. Trials 120 to 300 are shown for illustration. Color of the circles on top correspond to the option chosen by the participant. Green ticks indicate that the participant received reward on that trial, and red ticks indicate omission of reward. **B)** Richness, as estimated by the richness traditional RL model, with maximum-likelihood parameters that best explain the tracking of richness under this model. **C)** The likelihood of fitted standard traditional RL (standard trad. RL; dotted) and richness traditional RL (richness trad. RL; solid) models for each choice made by the participant. Chance at 0.5. Richness trad. RL has a better likelihood because the richness allows this model to have a more deterministic choice than standard-RL, even with similar Q-values. **D)** Q-values calculated by each model (see Methods). **E, left)** AIC values for additional extensions of traditional RL and foraging-RL models, fits to behavior from Experiment 1. "Richness choice" models incorporate both choice history dependence and richness terms (see Methods). **E, right)** Same for additional compare-alternative models from a dynamic belief class. exp-DBM is the exponential filtering approximation to the Dynamic belief model ([35], see Methods), DBM is the Dynamic Belief model as in [19] and exp is a fully fitted exponential filter ([35].

3) I was very confused by why the authors thought exp 2 was favouring an option comparison algorithm. Surely, if you want to design a task to favour option specific representations you'd give explicit counterfactual feedback instead of linking the options outcomes with each other. By linking the options, a threshold-based mechanism can be a perfect way to do the task as there is a threshold at which the alternative automatically has to be better. Additionally, there should be many previously published datasets available with counterfactual feedback, where they can test their hypothesis and directly pit two known and concurrently tracked option value models with switch stay comparisons that don't use the value difference, as this would be a nice and useful test of their theory.

We have amended the manuscript to better justify our choice of datasets and replicated the results in 2 additional datasets. Unfortunately, despite an extensive search and multiple emails and a personal conversation with the lead and last authors of the only counterfactual restless bandit papers we could identify (e.g., [16, 15]), we were unable to obtain this data to analyze. However, we did highlight this in the discussion as a vital direction for future research.

To the reviewers' point that different readers will have different intuitions than we did about what tasks should favor what computations, we have amended the manuscript to better justify the each experimental manipulation.

Results: "It remained possible that the results in Experiment 1 were a consequence of the specific environment we chose (i.e., a slow-moving bandit task with two independent option values that was conducted online). We therefore fit the models to 4 additional datasets in which we manipulated (1) the independence of option values (Experiment 2; two options), (2) the volatility of values (Experiment 3, 3-options), (3) the number of options (Experiment 4; 2-, 3-, and 4-options), and (4) the experimental setting (Experiment 5; in person, multiple response modalities). Experiment 2 allowed us to hold richness constant across the task and, along with Experiment 3, to bidirectionally manipulate the uncertainty of the unchosen option(s). Experiments 3 and 4 allowed us to determine how the number of available alternatives influenced model fit. Finally, because Experiments 1–4 were all conducted online, Experiment 5 replicated the original experiment in person, using both oculomotor and touchscreen response modalities.

In Experiment 2, participants (95 people, 41 females, 1 other or non reporting) performed a variation of the bandit task in the sum of reward probabilities always totaled one for any given trial (**Figure 5A-B**). This accomplished three things. First, especially in neuroscience, traditional RL models are often used to analyze behavior from tasks in which option values are tied (e.g., in probabilistic reversal learning tasks; [29, 23]), so this allowed us to determine if the foraging-RL model also does better in this circumstance. Second, having option values sum to 1 eliminates any variation of richness which, as we previously showed, influences compare-to-threshold choices but not compare-alternatives (Figure 2). We therefore reasoned that eliminating this variable might bias people towards compare-alternatives computations. Third and finally, this manipulation reduces the uncertainty of the unchosen option because its value can always be inferred from the chosen option (it was always 1 minus the value of the chosen option). At the group level, the foraging-RL model was again a better fit to human behavior than traditional RL (Figure 5C, *foraging-RL*: AIC = 14116.5, 3 parameters by 94 participants = 282 parameters; log-likelihood = -6776.25, *traditional RL*: AIC = 14746, relative AIC weight < 0.0001, 2 x 94 participants = 188 parameters; log-likelihood = -7185.02, 28,200 total trials). The foraging-RL model was also a better fit on an individual basis (*foraging-RL*: median individual AIC = 131.85, median individual log-likelihood = -62.93; *traditional RL*: median individual AIC = 139.11, median individual log-likelihood = -67.56; significant paired Wilcoxon signed rank test: $W = 1658, p < 0.04$).(Note that we were unable to fit the model in 1/95 participants because that participant repeatedly selected the same option 300 times ([1.1%]; similar to the proportion that did so in Experiment 1: 4/258 [1.5%]).) To understand why foraging-RL was a better fit, we again generated a traditional RL and foraging-RL agent for each participant's parameters and reward walk (see Methods) and compared each to the participant. In line with Experiment 1, foraging-RL better accounted for the participants' tendency to switch between options (Figure 5D-E, *foraging-RL*: root mean squared error (RMSE) = 0.043 (95% CI = [0.035,0.051]); *traditional RL*: RMSE = 0.079, (95% CI = [0.072,0.086]); Wilcoxon signed rank test $W = 3624, p < 0.0001$). Together, these results suggest that compare-to-threshold continues to be a better fit even when values are matched and richness does not vary.

We have also revised the rationale for Experiment 3:

Results: In Experiment 3, participants (270 people, 112 females, 2 other or non reporting) performed a 3-armed version of the bandit task in which we manipulated the volatility of the option values (see Methods, Figure 5F-G). The volatility manipulation was chosen because the traditional RL model failed to account for the stickiness of participants' behavior in Experiment 1. Because increasing volatility tends to increase switching behaviors [34, 11], we reasoned that this manipulation might bring participants more in-line with the switching dynamics in the traditional-RL model, giving this model a better chance of capturing participants' behavior. We increased the volatility of the environment across 3 levels: low, medium, or high levels (Figure 5H). (...)

We have also added new Experiments 4 and 5:

Results: In Experiment 4, participants (150 people, sex and gender not recorded) performed 2-, 3-, or 4-armed versions of the bandit task (Figure 5K-L). Because 3 alternatives were used in the volatility experiment (Experiment 3), but only 2 alternatives were used in Experiments 1 and 2, it was important to determine if changing the number of options was sufficient to systematically changed the goodness of fit of each model. Because the dimensionality of compare-alternatives computations scales with the number of options, it seems likely that the foraging-RL model might offer the greatest efficiency advantage as the number of options grows. Indeed, at the group-level, we saw that the foraging-RL model was a better fit than traditional-RL at the group-level (Figure 5M, relative *traditional RL* AIC weights for *2-armed* < 0.0001 ; *3-armed* < 0.0001; and *4-armed* < 0.0001; see Supplemental Table **??**), with the superiority of the foraging-RL model increasing directly with the number of options (2-armed : $|\Delta AIC| = 347$; 3-armed : $|\Delta AIC|$ =455 ; 4-armed : $|\Delta AIC| = 492$). The foraging-RL model was also a better fit at the individual level (*all environments (grouped)*: *foraging-RL*: median individual AIC = 263.7, median individual log-likelihood = -128.9; *traditional RL*: median AIC = 278.5, median log-likelihood = -137.3; Wilcoxon signed rank test $W = 3973, p < 0.005$). We next compared data simulated from each fitted model with the corresponding participant and found that the foraging-RL model again better accounted for participants' overall tendency to switch between options, although this difference was not statistically significant within the 50 participants in the 3-armed condition alone (Figure 5N-P, 2-armed: *foraging-RL*: RMSE = 0.045 (95% CI = [0.036, 0.054]); *traditional RL*: RMSE = 0.11, 95% CI = [0.095,0.12]; Wilcoxon signed rank test $W = 1227, p < 0.0001$; 3-armed: *foraging-RL*: RMSE = 0.093 (95% CI = [0.078, 0.1]); *traditional RL*: RMSE = 0.13, (95% CI = [0.12,0.14]); Wilcoxon signed rank test $W = 697, p = 0.16$; 4-armed: *foraging-RL*: RMSE = 0.056 (95% CI = [0.047, 0.067]); *traditional RL*: RMSE = 0.17, 95% CI = [0.16,0.19]; Wilcoxon signed rank test $W = 1055, p < 0.0001$). In sum, the relative fit of the foraging-RL model increased with the number of arms, perhaps reflecting the theoretical computational advantage that this strategy could offer by only tracking 1 value (versus 2, 3, or 4 values).

Finally, in Experiment 5, we performed an in-person experiment with 29 participants (19 females, 10 males) in which we manipulated the modality of the response within-subjects. This manipulation was meant address the fact that participants in Experiments 1-4 were recruited via the online platform Amazon mTurk, which limits environmental control and could reduce participant engagement, and to determine if similar results would be obtained regardless of response modality. In the lab, each participant completed two versions of a 2-armed bandit task: one using a touchscreen and another using eye-tracking device (**Figure 5Q**). Order was randomized and counterbalanced. Foraging-RL model parameters were reasonably correlated across testing blocks, though the threshold parameter ρ was the most stable (rho: $r = 0.79, p < 0.00001$; alpha: $r = 0.45, p < 0.002$; beta: $r = 0.35, p = 0.064$). These compared favorably with the parameter correlations observed with a traditional RL model (alpha r = 0.423; p = 0.0223; beta r = -0.062, p = 0.7477). The foraging-RL model again provided a better fit to human behavior than the traditional RL model at the group level, independently of the response modality (touchscreen or eye-tracking) and condition order (**Figure 5R**, relative *traditional RL* AIC weights for all presentation orders and response modalities $< 10^{-15}$, see Supplemental Table 6). While model comparison results were not significant at the individual level in this small sample, we again found that, the foraging-RL better accounted for individual participants' tendency to switch between options in both the eye-tracking task, and the touchscreen task (**Figure 5S-T**; eye-tracking: *foraging-RL model*: RMSE = 0.045 (95% CI = [0.036, 0.054]); *traditional RL*: RMSE = 0.11, (95% CI = [0.095,0.12]); significant Wilcoxon signed rank test, $W = 377, p < 0.0006$); touchscreen: *foraging-RL*: RMSE = 0.061 (95% CI = [0.053, 0.069]); *traditional RL*: RMSE = 0.099, (95% CI = [0.091,0.11]); significant Wilcoxon signed rank test, $W = 398, p < 0.0001$)). Further, we also found that the foraging-index, a measure of the relative goodness of model fit (see Methods), was correlated across modalities ($r = 0.63, p < 0.0005$). Overall,

these findings show that participants consistently used the compare-to-threshold strategy, regardless of the responses modality (touchscreen vs. eye-tracking) and the environmental setting (online vs. in-person).

Because we agree with the reviewer that giving explicit feedback about unchosen option would have been a useful addition to the manuscript, we did an extensive search for a counterfactual bandit dataset we could use here. We searched the literature using multiple keywords, including "counterfactual feedback," "complete vs. partial feedback," "unchosen option feedback", and "full outcome" However, all public datasets we identified were structured around stationary, block-based tasks rather than the restless bandit environments needed to estimate these models. We also reached out directly to researchers who have worked on counterfactual feedback in bandit paradigms (e.g., Valentin Wyart and Charles Findling), but have not yet received a response. Given the need to respond in a timely fashion, we ultimately chose to addresses the utility of this particular kind of dataset in the discussion with some thoughts about how we would approach it.

In the discussion, most relevant part bolded: Although the majority of participants' choices were better described as a compare-to-threshold process, we did find evidence of compare-alternatives computations in a small minority of participants (1.2%, 4.3%, 2.2% or 7.8%, depending on the analysis). We cannot rule out the possibility that this proportion would be higher in different tasks or in unexplored variants of this task (e.g., change point tasks, static bandits, bandit problems where reward magnitude drifts rather than probability, or bandit problems without feedback noise). Because the compare-to-threshold strategy is both computationally simpler and less rewarding than the compare-alternative strategy, it seems plausible that different results might be obtained if the stakes were higher and/or the cognitive demands lower. Indeed, when we raised the demands, increasing the number of options and he dimensionality of the task, the advantage of the foraging-RL model also grew—an intriguing parallel to the computational advantage that could come from adopting a strategy that need track only 1 value in increasingly high-dimensional environments. Of course, future within-subjects work is needed to determine if this result reflects a change in strategy across individuals or just some improved ability to discriminate the models in higher-dimensional environments. **One manipulation that would be especially important in future studies is full outcome feedback (where the outcome of both chosen and unchosen options are explicitly provided, e.g., [16, 31]. Full feedback might encourage compare-alternatives strategies [16], though it would be important to differentiate true compare-alternatives computations (where option values are tracked independently over time) from a compare-to-threshold strategy that operates on differenced values (i.e., where relative value is calculated at the time of encoding, rather than at selection).** Of course, we do not mean to exclude the possibility that humans use a repertoire of strategies, switching between them as needed. For example, in the 3-alternative task, a substantive proportion of participants (16%) did not explore alternative options uniformly, though roughly equal proportions of these were biased towards the best alternative (consistent with using a compare-alternatives strategy for exploration) or towards the worst (consistent with an as-yet-unidentified exploratory strategy). While more work is clearly needed, these results do suggest that compare-to-threshold models should be evaluated even in tasks that are conventionally modeled using compare-alternatives computations.

4, part 1) While I very much like their results of Choice Run and HMM results, I feel like very similar data could be obtained with a stickiness model/ choice perseveration kernel. Fundamentally, the problem is that the currently state of the art models of option comparison would be perfectly fine with adding choice perseveration (as they have added in some comparisons between model classes and which reduced the difference in model fit considerably (see F3J+K). The question is whether addition of some type of normalization and/or inference using the richness of the currently experienced environment to change the value expectation about alternatives, together with stickiness would be sufficient to explain their data completely. The difference between such a model from theirs would be that fundamentally comparisons would still be based on option specific representations, not option indifferent stay-switch comparisons.

We have added the additional choice perseveration + richness models suggested by the reviewer. Although these are the best models we fit, their inclusion does not substantively change the conclusions: foraging-RL is still a better explanation for human data, when we add comparable extensions to both algorithms.

This is described as follows in the results: A variety of extensions and alternatives are known to outperform the standard 2-parameter traditional RL model, and it remained unclear whether any of these would fit behavior better than the foraging-RL model. Therefore, we next compared the foraging-RL model with 10 additional models: 4 common extensions of the traditional RL model (asymmetrical learning, value decay, and 2 forms of choice history dependence), 2 dynamic Bayesian models and a free-fitting exponential filter (see Supplementary Figure S3E), and **3 novel extensions meant to address limitations of the traditional RL model identified here (see Methods)**. (...) Foraging-RL also outperformed 3 extensions to RL models that were developed to account for our behavioral observations: an RL model in which values are explicitly normalized by richness (see Methods; "richness"; Supplementary Figure S3E; Supplementary Table 1), a repetition bonus model that implements a tradeoff between repeating the last option and making decisions based on relative value (see Methods; "repetition bonus"; Supplementary Figure S3E; Supplementary Table 1), and **a model that combined richness normalization with the best formulation of choice-history dependence (see Methods; "richness + choice + 2"; Supplementary Figure S3E; Supplementary Table 1)**. (...)

Critically, any one traditional RL model could have performed better than the standard foraging-RL model for two reasons: either because it benefits from the compare-alternatives architecture or because decades of incremental engineering were ultimately able to overcome some of the limitations of that architecture. Therefore, we next compared foraging-RL models with traditional RL models as a class. We developed an equivalent version of the foraging-RL model for each extension of the traditional RL model (see Methods; Figure 3J-K; Supplementary Figure S3E). Note that our goal here was not to build the best foraging-RL model we could, but instead offer a fair compare-to-threshold version of each common variant of the traditional RL model. We found that every extended foraging-RL model outperformed the equivalent traditional RL model at the group level (Figure 3J-K, S3; Supplementary Table 2), suggesting that compare-to-threshold models outperformed compare-alternatives models as a class. (...)

(4, continued) Ultimately, the best test of their theory would be whether in a multi-option data set, participants retain the object specific value representations after repeated switches, and therefore attach the value to specific options, not simply switch stay behaviours (which could be done easily with a task with multiple option values and counterfactual feedback as suggested in point 3). Additional tests could manipulate the value of the environment independently to see what impact that has on switching behaviour (if any) and whether these effects are better explained through normalization mechanisms based on environmental richness or an explicit switch vs stay decision explanation. However, I suspect that they'd find a mixture of behavioural drives, i.e. a limited retention of option specific reward information (likely based on working memory) and a tendency to stick with previous options, particularly if they appear good enough (in fact, satisficing has been a very empirically useful concept in ecological behavioural modelling, psychology and economics).

We performed a detailed analysis of the effects of alternative option value in our largest 3 alternative dataset (Experiment 3; supplementary Figure S5). The results did not change our overarching conclusion, but do point to important future directions for both models. Briefly, the majority of participants did not retain alternative option values and those who did tended to chose the worst alternative–not the best. This is difficult to reconcile with a traditional RL model, although perhaps a good compare-alternatives model could be engineered that is capable of reversing its representation of values from time to time. Alternatively and more simply, we believe that a foraging model could account for all these observations if it had the working memory capacity to track (1) the identity of the last exploited option, (2) the identity of the most uncertain (least recently selected) option. This is described in the text as follows:

Results : Because 3 targets were used in Experiment 3, we could also leverage this dataset to determine how the value of alternatives influenced choice. In a 3-alternative setting, compare-alternatives computations would be more likely to switch to the best alternative option where compare-to-threshold computations would explore uniformly because alternative values are not tracked. During exploratory switching (Figure S5A-B; see Methods), a minority of participants had some bias between the two alternative options. However, the majority of these chose the worst alternative option, not the best. By contrast, traditional RL models would predict a large number of individuals with a bias towards the best alternative, not the small number seen in practice (i.e., 2.4%-7.8%, 5-18 people, depending on method; 2.5% expected by chance). However, the fact that 35-46 participants had any bias at all was also not

consistent with the assumption that exploration is random and that unchosen option values are forgotten instantaneously in the foraging-RL models. This was especially apparent in switch-backs—decisions that revert to a previously exploited option (compare Figure S5C and D)—implying that adding some encoding of the last exploited option's identity could improve the foraging model substantially. Similarly, adding some capacity to preferentially explore options that have not been recently sampled would likely both improve the foraging model and account for the results in Figure 4G and S5A, B, and D) because these options are statistically more likely to be lower value than those that the model has recently chosen, given that the model tends to choose good options (Figure 3C-F). In short, while a minority of participants exhibited biases between alternative options in the 3-option case, rectifying these observations with the foraging model could be accomplished through adding a working memory component that recalls (1) the last exploited option, and (2) the most uncertain option, without tracking alternative values.

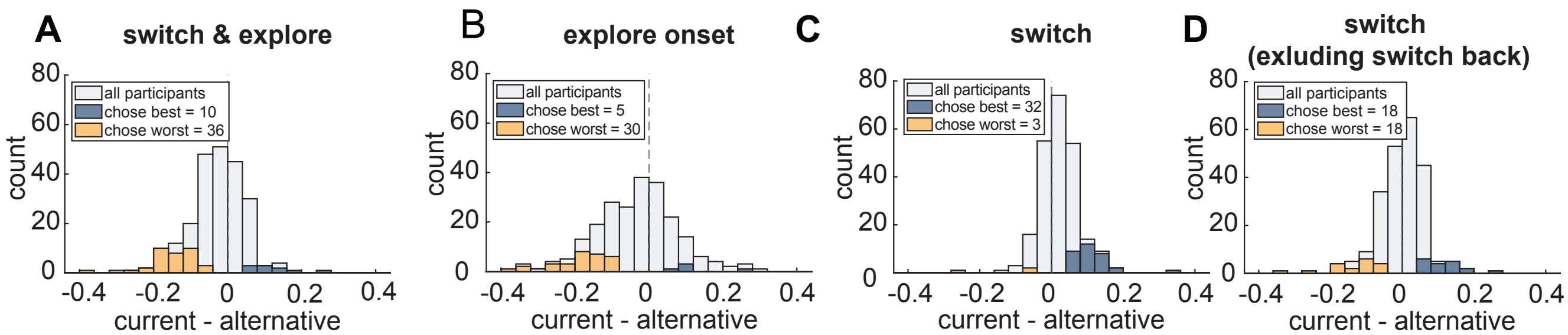

Figure S5: **Switching preferences among the alternative options in Experiment 3 A)** Because 3 options were used in Experiment 3, we were able to ask if participants preferentially switched to the best or worst of the alternatives. We first focused on exploratory switches: switch decisions that were also identified as exploratory via the HMM (see Methods). Distributions (*grey*) show the average difference in objective option values for these decisions within each participant. Positive values indicate participants who switched to the objectively better alternative, negative values indicate participants who switched to the objectively worse alternative. Participants that chose the best alternative significantly more frequently than chance are in blue. Participants that chose the worst alternative more frequently than chance are in orange. Chance was estimated by running 50,000 bootstrap simulations of random choices for each individual, significance tested at $p < 0.05$, uncorrected. **B)** Same as *A*, but specifically for the first explore trial in a sequence ("onset" of exploration) which is a switch decision by definition. **C)** Same as *A*, but for all switches, many of which are "switch backs", returning to the previously exploited option. **D)** Same as *C*, but excluding the switch backs to the last exploited option.

We believe we have already addressed the other points raised in this comment by (1) describing how we modified both models to include normalization by richness and (2) showing that Experiment 3 offers an environment in which richness is held constant, but the foraging-RL model still outperforms. We would add that we agree that further manipulations of richness would be a valuable direction for future work, but this work is being led by another student in the lab as their MSc project.

5) I didn't understand why the authors did not use simple logistic GLM approaches throughout the manuscript? Specifically, I don't understand the whole point of the strategic finger print fitting of the simulated binned data. I might be mistaken but the data can be understood and analyzed perfectly well with a regression approach in which you test for the richness (Value A+B) and value discriminability/Value difference (ValueA-B) in GLMs. If you then predict the y=switch, you can simply test the impact of richness on switching, while linearly controlling for value difference. If you want you can even throw other additional terms and shapes in, if you are concerned about non-linearities. So far, I don't understand the additional value of a PCA derived 7 dimensional fingerprint instead of going with commonly accepted, appropriate and more easily interpretable logistic regressions, particular given the nature of their data. With such an approach you can simply test the intercept of the GLM (or if you want to argue bias you can fit your own softmax equivalence point) to measure staying bias and the impact of richness on switching behaviours to proof sensitivity of environmental context on switching. However this would probably show that there is an additional impact of RL derived option value difference on switching, which would suggest a mixture of effects.

If they see literally no impact of value difference in such a GLM (in a paradigm in which the option values fluctuate independently, meaningfully and beknownst to the participants), I would consider they have evidence of a lack of option comparison in their data.

We have added a logistic GLM approach to the manuscript, which supports our overarching result that people compare-to-threshold rather than compare-alternatives. We have also clarified our fingerprint analyses.

The logistic GLM suggestion is related to major comment #1 from Reviewer 2 and 3. In order to address these comments we used a GLM to independently estimate the contributions of the chosen and unchosen option values to choice. If, as the reviewer suggests, the difference in value is calculated, then we should see positive beta weights for chosen options and negative beta weights for unchosen options (producing a difference in values). This was true in a very small minority of participants (3 / 258 had opposite signs for Value A and B, to use the reviewer's terms).

The idea that foraging would predict positive signs for both Value A and B is, we believe, a misunderstanding related the lack of clarity in our original description of the fingerprint analyses (this has been corrected here, see below). The compare-to-threshold approach does not calculate or operate on richness, it only calculates the value of the focal option. So, in the reviewer's terms, the specific prediction this view would make is that Value A would be encoded, but Value B would not. Indeed, that's what we see in 115 / 258 participants: positive beta weights for chosen options and beta weights near zero for unchosen options. Further, model comparison did not suggest that including a term for unchosen option values was necessary.

Changes to the manuscript to describe the new logistic model are as follows:

Methods:

Choice predictive Generalized Linear Model (GLM)

To determine whether the value of previously unchosen options influenced choice, we used logistic regression to predict the probability of repeating choice on a trial-by-trial basis for each participant. We defined the log odds of $p(\text{repeat}_t)$ as

$$log\left(\frac{p(\text{repeat}_t)}{1-p(\text{repeat}_t)}\right) = \beta_0 + \beta_1 V(\text{chosen}_{t-1}) + \beta_2 V(\text{unchosen}_{t-1}) \ , \qquad (2)$$

where $V(\cdot)$ is the objective value of the option that was chosen or unchosen at time $t-1$. In this formulation, decisions that depend on comparing values would have positive beta weights for $V(\text{chosen}_{t-1})$ and negative beta weights for $V(\text{unchosen}_{t-1})$, indicating that the probability of choice is proportional to $V(\text{chosen}_{t-1}) - V(\text{unchosen}_{t-1})$. Conversely, decisions that depend on comparing to a threshold would only have positive beta weights for $V(\text{chosen}_{t-1})$, because only the focal option value is tracked.

Objective values were used because these offered the fairest comparison between views. We could not estimate subjective values without making an assumption about whether the participants are using compare-alternatives or compare-to-threshold computations. Because models were fit on a trial-by-trial basis, $p(\text{repeat})$ was a binary vector labeled as 1 whenever the choice on the trial t was the same as the choice on trial $t-1$.

To estimate the relative contribution of each predictor, we also fit two reduced models: one containing only $V(\text{chosen}_{t-1})$ and another containing only $V(\text{unchosen}_{t-1})$. In Figure 1F-G, the p-values for the coefficients of interest ($V(\text{chosen}_{t-1})$ and $V(\text{unchosen}_{t-1})$) obtained from the full model were adjusted using a Holm-Bonferroni correction within each predictor. Model comparison was evaluated with the Bayesian Information Criterion because we found that the Akaike Information Criterion had an insufficient penalty for the number of parameters (i.e., the full model was generally preferred even when option values were completely randomized). Participants who never switched were excluded from this analysis because model parameters were not identifiable.

Then, a new result section was added to the revised manuscript:

Participants largely ignored the value of the unchosen option

The compare-to-threshold view predicts that the probability of choosing an option is only determined by the prior value of that option, not the value of any alternative options. Therefore, choices should be entirely insensitive to the value of unchosen alternatives. By contrast, decisions should depend on the difference between chosen and unchosen values in the compare-alternatives view. To determine which view better described participants' choices here, we developed a generalized linear model (GLM) that predicted choice from the reward probabilities of the previously chosen option ($V(\text{chosen}_{t-1})$) and the previously unchosen option ($V(\text{unchosen}_{t-1})$; see Methods). We found that the value of the chosen option was a strong positive predictor of the decision to repeat a choice (Figure 1F, *left* : sig. positive in 118 / 254 participants). However, the value of the unchosen option had minimal influence (Figure 1F, *right* : sig. positive in 2 / 254 participants; sig. negative in 11 / 254 participants). Further, of the 11 participants who had a negative $V(\text{unchosen}_{t-1})$ term, only 3 also had a significant positive $V(\text{chosen}_{t-1})$ term. This meant that only 3 out of 258 participants (1.2%) were value "comparators" (Figure 1F, *right*) in the sense predicted by the compare-alternatives view, and the vast majority of participants (98.8%) were not. Model comparison revealed that a model which included only $V(\text{chosen}_{t-1})$ outperformed a full model (containing both $V(\text{chosen}_{t-1})$ and $V(\text{unchosen}_{t-1})$) (Figure 1G). The $V(\text{chosen}_{t-1})$-only model was also better fit to the majority of individual participants (better fit in 174 / 254 participants; $wBIC$ of full and $V(\text{chosen}_{t-1})$-only models relative to $V(\text{unchosen}_{t-1})$-only model (both < 0.0001)). Together these results suggest that the value of the previously chosen option was the primary driver of choice, with minimal influence from unchosen option values, consistent with the compare-to-threshold view.

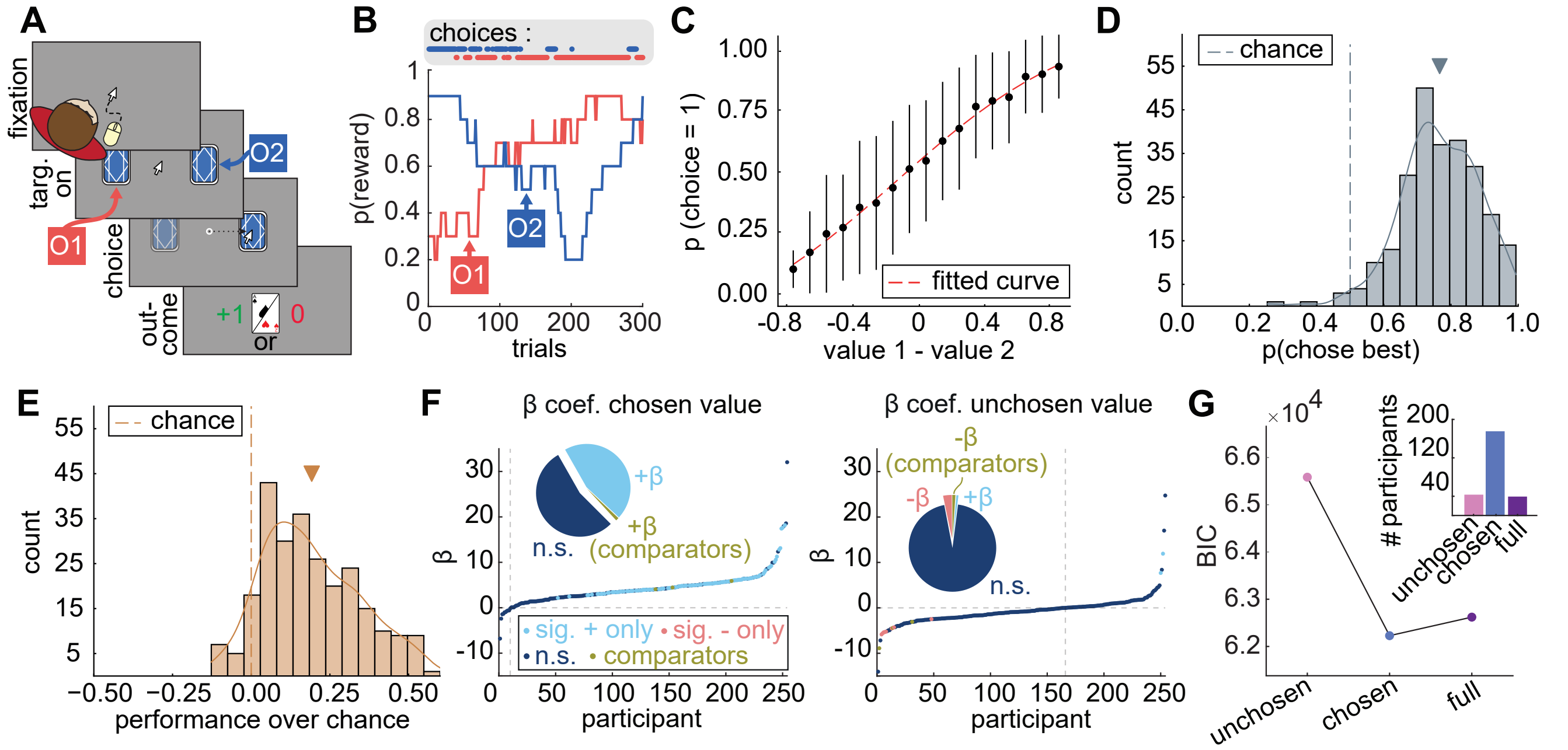

Figure 1: **Experiment 1 task and baseline behavior. A)** Participants (n = 258) chose between 2 face-down decks of playing cards that were composed of aces and 2's. Participants were told that the proportion of aces and 2's in each deck would change over time and that decks could be good, bad, or mediocre right now, but would not stay that way the whole time. Participants got a point (and \$0.02) for each ace they found, but no points for 2's. **B)** The reward probability of each option (red trace = option 1; blue trace = option from an example reward schedule, with participant choices along the top (red dots = option 1; blue dots = option 2). **C)** Average probability of choosing option 1 as a function of the difference in the objective reward probability ("value") between option 1 and 2. Error bars = standard error of the mean (SEM) across participants. **D)** Distribution of the number of trials in which each participant chose the objectively best option. Dotted line = chance, caret = mean across participants. **E)** Same as *D*, for the percent of trials in which participants were rewarded, normalized as percent of chance performance. **F)** Predicting choice from the value of chosen and unchosen options. Beta coefficients for ($V(\text{chosen}_{t-1})$) (*left*) and $V(\text{unchosen}_{t-1})$ (*right*). Each circle represents a single participant, sorted by coefficient value. The dashed horizontal line is at zero (no effect). Participants with a significant positive betas are light blue, significant negative betas in red, and non-significant betas in dark blue. Gold circles identify "comparators" who had a significant positive weight for the chosen option and negative weight for unchosen option value. Significance is Holm-Bonferroni corrected within predictor ($\alpha = 0.05$). Insets illustrate the proportion of participants in whom each term was significant, colored by sign and identifying all "comparators". **G)** sum of BIC for each model ($V(\text{unchosen}_{t-1})$-only, $V(\text{chosen}_{t-1})$-only, and the full model which contains both terms). Inset) The number of individual participants best fit by each model, based on the Bayesian Information Criterion (BIC).

We have also substantially revised the manuscript to clarify the fingerprint analyses and the relationship between the foraging algorithm and richness. Briefly, goal of the fingerprint analyses was to illustrate that certain environmental variables (i.e., richness and discriminability) can *differentiate* compare-alternatives and compare-to-threshold algorithms. This was not meant to imply that these variables are explicitly calculated by any particular algorithm, but we understand that the original manuscript was insufficiently clear on this point. Therefore, in multiple places in the revised manuscript, we now clarify the point that compare-to-threshold algorithms depend on richness only as a *byproduct* of the calculations they are performing. This is because the value of a focal/chosen option is more likely to be high in a rich environment, so a compare-to-threshold algorithm would be more likely to exploit in a rich environment, compared to a poor one. We have also added a new figure panel that makes this point explicit (Figure 2B). We agree that our fingerprint approach is novel, but continue to believe that it has value both as a complement to our model-based analyses and, in the revised manuscript, as an illustration of the central computations involved in each algorithm. Ultimately, the fingerprint method is presented as complementary to other approaches, not as a substitute for them, and we believe that other researchers may find value in this approach in the future.

The relevant sections of the text now read:

Methods:

Strategy Fingerprints

Compare-alternatives and compare-to-threshold decision-making should, in theory, depend on different aspects of the environment. To quantify these predicted dependencies we characterized the fingerprints of each strategy as a function of two features of the environment: richness (the sum of the values) and discriminability (the range of the values, normalized to the sum). In order to make predictions for each strategy, we first separated the 258 reward walks experienced by the participants into 100-trial bins (3 bins per session, 774 bins). We then calculated the richness and discriminability within each bin (Figure 2C, *bottom*) and the "environmental predicted p(switch)" for each bin, given each of the two strategies (Figure 2D-E). Because exploration should occur most often when option values are close together in the compare-alternatives strategy, the environment-predicted p(switch) under this strategy was calculated as the probability that the difference in option values was below some Δ reward for various $\Delta rewards$ (4 examples $\Delta rewards$ in Figure 2D). Because exploration should only occur when the focal option is below threshold in the compare-to-threshold strategy, the environment-predicted p(switch) under this strategy was the probability that any randomly selected "focal" option value would be below some threshold for various thresholds (4 examples thresholds in Figure 2E).

To quantify how similar participants' switching patterns were to the compare-alternatives and compare-to-threshold fingerprints, we estimated the shape of the environmental predicted p(switch) at each parameter value for each strategy (i.e., threshold or $\Delta reward$). We did this via fitting generalized linear models (GLMs) to the environment-predicted p(switch) from both richness and discriminability. Two models were fit, a linear (2 parameter) GLM and a quadratic (3 parameter) GLM, for each combination of parameter, strategy, and independent variable. We then extracted 3 key features from each pair of fitted models: (1) the relative deviance of the linear and quadratic models (a measure of the extent of curvature as a function of either richness or discriminability), (2) the quadratic term of quadratic model (a measure of the sign and magnitude of curvature in the relationship), and (3) the linear term of the linear model (a measure of the overall slope of the relationship). This approach allowed us to precisely quantify how switch probabilities were predicted to change as a function of both richness and discriminability under each hypothesized strategy. Distributions of the features for every possible threshold and $\Delta reward$ are illustrated in S1.

To consider the fingerprints holistically, rather than as individual features, we then took the 6-feature dimensions described above, added the overall probability of switching to account for DC offsets in switch probabilities as a 7th feature, and then calculated the distance from the human fingerprint to the predicted fingerprint for every possible parameter value for each of the two strategies (Figure 2H). PCA was performed only to illustrate the approach in Figure 2G, where distances were calculated in the full space. In this plot, bounding boxes are drawn around all environment-predicted fingerprints under each strategy to illustrate the range of fingerprints that each strategy was capable of generating. Similar results were found regardless of whether GLMs were fit with binomial or identity link functions. The former appears in Figure 2G, the latter is shown in Figure S1A-C).

Results :

Human behavior better resembles the fingerprint of compare-to-threshold decisions

The compare-alternatives and compare-to-threshold views also differ in their predictions about how people should decide to explore as a function of the environment (Figure 2A-B). Specifically, the compare-alternatives and compare-to-threshold views predict that switching will be most frequent in different situations because there are different mechanisms for exploration in the two cases. The compare-alternatives view predicts that people are the most likely to explore when it is difficult to discern which option is the best. Switching would therefore occur most frequently when the option values are close together (i.e., when the $\Delta reward$ between the options is small; Figure 2A). This happens most

often in **ambiguous** environments, rather than *discriminable* environments. Conversely, compare-to-threshold view predicts that people explore whenever an exploited option falls below a threshold (Figure 2B). Switching would therefore occur most frequently when the value of any option is more likely to fall below a threshold. This happens most often when all option values are low, in **poor** environments rather than *rich* environments, when no option can fall below threshold. Note that this is true even without assuming that the compare-to-threshold algorithm calculates richness: compare-to-threshold choices will inevitably depend on richness as a byproduct of the fact that options are more likely to be above threshold in rich environments. In short, switching should depend on different statistics of the environment in compare-to-threshold decision-making versus compare-alternatives decision-making.

These insights imply that we should be able to extract distinct "fingerprints" of these two strategies as a function of the environment. Regardless of implementation, switching probability in compare-to-threshold decision-making should depend on richness (the sum of values), while it should depend on discriminability (the differences between values) in compare-alternatives decision-making. However, in any bounded environment the sum of values and difference between values will be related (Figure 2C; i.e., any time outcomes are probabilistic or there are floor and/or ceilings on outcome magnitude). When richness is high (value of all options are close to their maximum) or low (value of all options close to their minimum), discriminability can only be low. However, when richness is at intermediate levels, discriminability can range from low (e.g., all values around their midpoint) to high (e.g., a mix of probabilities close to min and max). As a consequence of these bounds, compare-alternatives decision-making should have a U-shaped relationship with richness, with minimal switching at intermediate levels where discriminability is highest (Figure 2D, *bottom*). By contrast, compare-to-threshold decision-making will have an inverted U-shaped relationship with discriminability, driven by the statistical relationship between richness and discriminability (Figure 2E, *top*) and both strategies have a unique fingerprint as a function of both variables (Figure 2D-E).

We found that the participants switched options the most at intermediate, rather than low levels of discriminability (Figure 2F, *top*), consistent with compare-to-threshold computations (Figure 2E,*top*). The participants were also less likely to switch in rich environments, compared to poor ones (Figure 2F, *bottom*), again consistent with compare-to-threshold computations (Figure 2E, *bottom*). To quantify the relationship between participants' data and the compare-alternatives and compare-to-threshold fingerprints, we calculated the probability of each strategy switching frequently as a function of discriminability and richness (i.e., environment predicted p(switch); Figure 2D-E; see Methods). We then measured the slope and curvature of the relationship to discriminability and richness under each hypothesis (Figure 2G-H; see Methods). Human switching was sensitive to discriminability and richness in a way that better resembled compare-to-threshold decision making, both in terms of the individual features (Figure S1A-C) and in a multivariate feature space that considered all features simultaneously (Figure 2G-H and Figure S1D-E; median distance to $\Delta reward$ in the full feature space = 39.41, range = [22.04, 437.07]; to threshold = 6.80, range = [3.76,9.78] ; *two-sided Wilcoxon rank sum test*, $p < 0.001$; see Methods). While the human data was not a perfect match for either fingerprint, it better resembled the fingerprint of compare-to-threshold decision-making than the fingerprint of compare-alternatives decision-making.

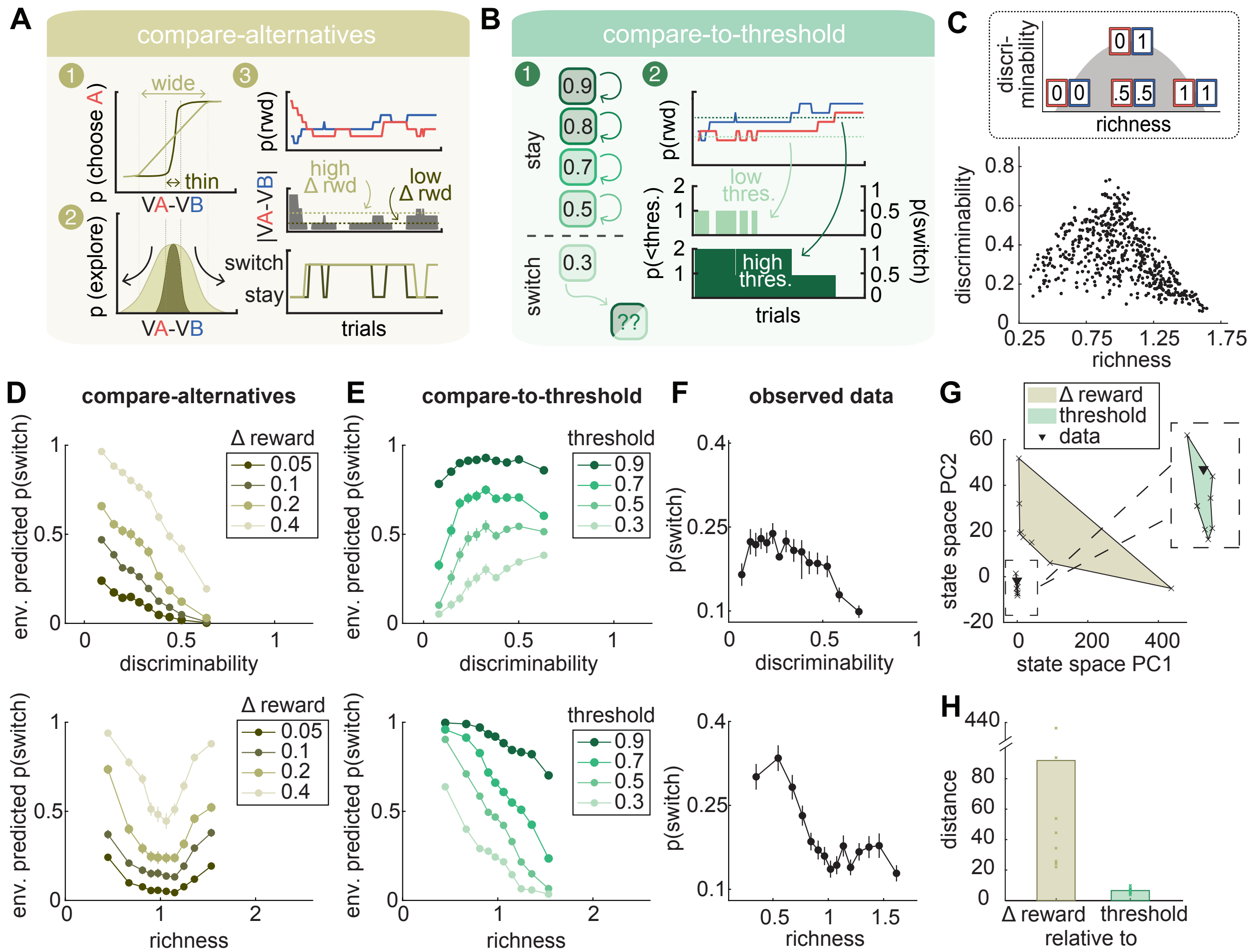

Figure 2: **Dissociable signatures of compare-to-threshold and compare-alternatives decisions to explore as a function of environmental statistics. A)** In compare-alternatives decision-making, exploration is driven by noise in value comparison. This is typically modeled via a softmax decision rule in which noise is larger when values are close together. Noise scales with inverse temperature, such that exploration occurs across a wider range of value differences when inverse temperature is low, compared to when it is high. In either case, switching occurs the least often at the tails, when differences in value are highest. As a result, we can predict how often participants should switch from the discriminability of the environment in this cartoon. **B)** In compare-to-threshold decision-making, the decision to stay depends only on the value of the focal option. If the value drops below some threshold, the algorithm switches. This means that switching essentially does not occur when all options are above threshold and occurs in proportion to the number of options that are below threshold otherwise. As a result, we can predict how often participants should switch from the richness of the environment in this cartoon. **C)** *top*: Cartoon illustrating the relationship between richness and discriminability for 4 example pairs of probabilistic rewards. *bottom*: Discriminability as a function of richness in the reward walks in Experiment 1. Each dot represent discriminability and richness in a 100-trial bin (see Methods). **D)** Probability of the difference in option values being below some $\Delta reward$ ("environment predicted p(switch)" for compare-alternatives) as a function of discriminability (*top*) and richness (*bottom*) for 4 values of $\Delta reward$ (*shades of gold*). **E)** Probability of any random option value below some threshold ("environment predicted p(switch)" for compare-to-threshold) as a function of discriminability (*top*) and richness (*bottom*) for 4 threshold values (*shades of green*). **F)** Probability of switching for all participants in Experiment 1 as a function of different levels of reward discriminability *(top)* and richness *(bottom)* of the environment (errorbar = SEM). **G)** First 2 PCs of the multivariate feature space of compare-alternatives fingerprints (*gold*) and compare-to-threshold fingerprints (*green*) (see Methods). Bounds encompass all possible values for the $\Delta reward$ and threshold parameters. x = each parameter value, black caret = humans. **H)** Distance between humans and every (*gold*) and (*green*) in the full 7-dimensional space. x = individual parameter values.

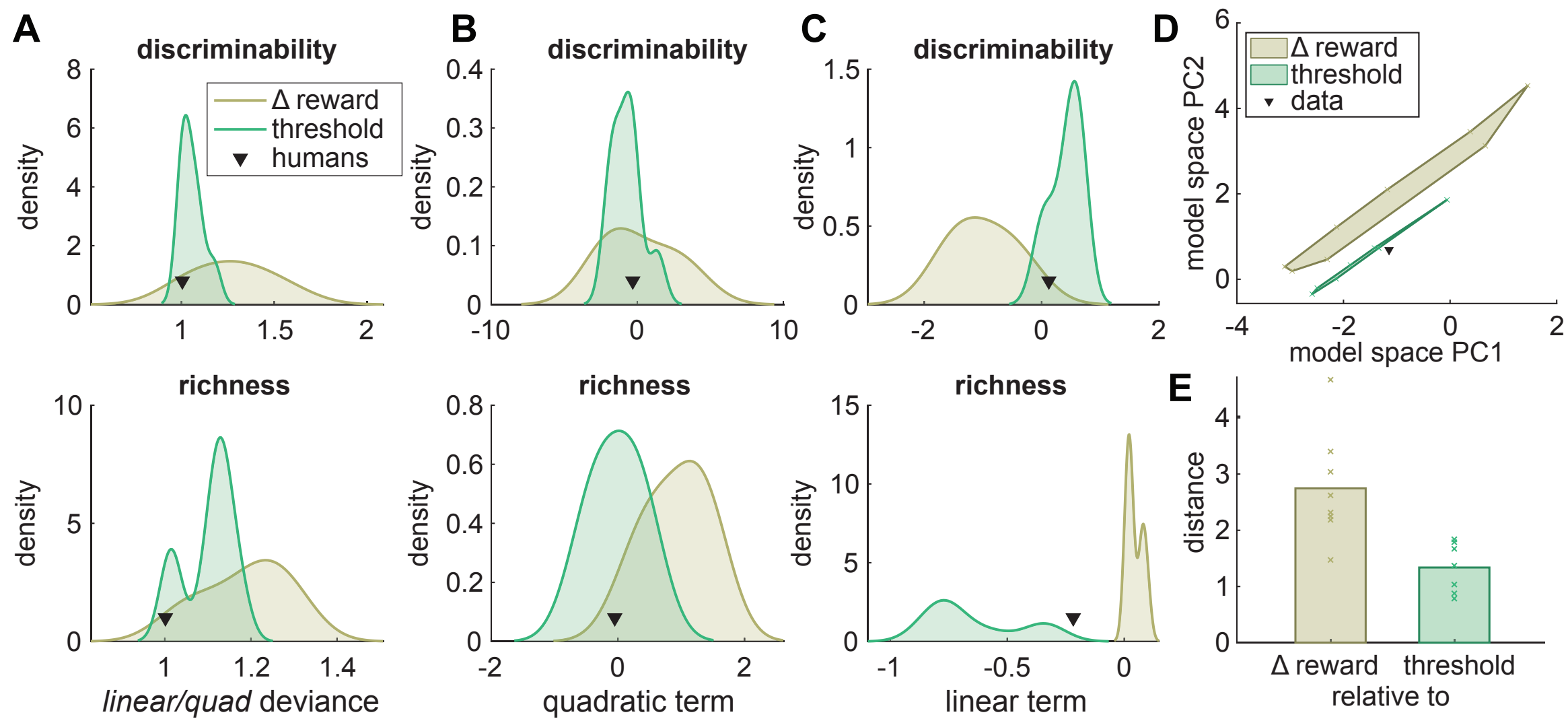

Figure S1: **Quantifying the fingerprints of compare-to-threshold and compare-alternatives decision to explore**. **A)** Distribution of deviance ratio between linear and quadratic model fits to compare-alternatives (*gold*) and compare-to-threshold (*green*) environmental predicted probability of switch (Figure 2D-E) as a function of the discriminability *(top)* and richness *(bottom)* of the environment (black caret = humans). **B)** Distribution of the quadratic term (i.e., curvature) of the quadratic model fit to compare-alternatives (*gold*) and compare-to-threshold (*green*) environmental predicted p(switch) (Figure 2D-E) as a function of discriminability *(top)* and richness *(bottom)* of the environment (black caret = humans). **C)** Distribution of the linear term (i.e., slope) of the linear model fit to compare-alternatives (*gold*) and compare-to-threshold (*green*) environmental predicted probability of switch (Figure 2D-E) as a function of discriminability *(top)* and richness *(bottom)* (black caret = humans). **D** Same as Figure 2G, but for the linear GLM, rather than the binomial model. First 2 PCs of the multivariate feature space of compare-alternatives fingerprints (*gold*) and compare-to-threshold fingerprints (*green*). Bounds encompass all values for the $\Delta reward$ and threshold parameters. x = each parameter value, black caret = humans. **E** Same as Figure 2H, but for the linear GLM (identity link function), rather than the binomial model. Distance between humans and every (*gold*) and (*green*) in the full 7-dimensional space. Bars = mean distance, x = individual parameter values.

6) In the same vein of unusual behavioural modelling, I didn't understand why the authors use a delta reward rule for switching for the simulations rather than just simply using a softmax on the value difference with a fitted or different simulated biases terms? If I understood their procedures simulation correctly it is almost what they are doing, except that they are using unsigned, rather than signed difference, but it would be way easier to understand if they just described it as a switch-stay bias term of a softmax.

Given where this comment falls in the sequence, we believe that this comment is referring to Figure 2D-E. As detailed above in response to point 5, we have made extensive changes to clarify these analyses in the Results, Figures, and Methods sections. To address this specific point, it may help to restate the point we clarified above: that these are not simulations, but instead analyses of the statistics of different random walks, so a softmax decision rule would not be appropriate here. We did use softmax decision rules for all simulations.

7) The discriminability richness relationship (shown in figure 2) is not a necessity of decision making, but more an artefact of the task design. I am not sure why they did not collect at least one data set without those constraints to prove their points?

The sum of option values and the difference between those values are necessarily coupled whenever values are bounded. This relationship between discriminability and richness will arise whenever rewards are probabilistic, for example, or whenever reward magnitudes are subject to floor and/or ceiling effects. So,

this is not an artefact of our specific task design, but, instead, a general property of most decision-making environments. This means that creating a task in which discriminability and richness are fully decoupled would require an unbounded reward structure. We did not pursue this for two reasons. First, it would be nearly impossible to implement, given the need to balance the limits of human discriminability with a finite research budget and ethical concerns regarding overly large participant payments. Second, decoupling these quantities is not required to test our hypotheses. Even in a bounded environment, we were able to observe a clear double dissociation between compare-to-threshold's relationship to both richness and discriminability and compare-alternative's relationship to both richness and discriminability. This dissociation was sufficient to test our stated hypotheses.

We have endeavored to make this fine point more clear in the Results: These insights imply that we should be able to extract distinct "fingerprints" of these two strategies as a function of the environment. Regardless of implementation, switching probability in compare-to-threshold decision-making should depend on richness (the sum of values), while it should depend on discriminability (the differences between values) in compare-alternatives decision-making. **However, in any bounded environment the sum of values and difference between values will be related (Figure 2C; i.e., any time outcomes are probabilistic or there are floor and/or ceilings on outcome magnitude).** When richness is high (value of all options are close to their maximum) or low (value of all options close to their minimum), discriminability can only be low. However, when richness is at intermediate levels, discriminability can range from low (e.g., all values around their midpoint) to high (e.g., a mix of probabilities close to min and max). **As a consequence of these bounds, compare-alternatives decision-making should have a U-shaped relationship with richness, with minimal switching at intermediate levels where discriminability is highest (Figure 2D, *bottom*). By contrast, compare-to-threshold decision-making will have an inverted U-shaped relationship with discriminability, driven by the statistical relationship between richness and discriminability (Figure 2E, *top*) and both strategies have a unique fingerprint as a function of both variables (Figure 2D-E)**.

8) I am also a bit puzzled why they sometimes write "RL vs Foraging" as the "Foraging" model also uses an RL algorithm to update value. the option based choice vs environment comparison vs switch seems more valid.

The revised manuscript and all figures now consistently frame the paper around differentiating "compare-alternatives" calculations from "compare-to-threshold" calculations. This aligns the terminology with the central theoretical question, rather than with any specific implementation of these computations. We then refer to the class of compare-to-threshold RL models that we develop here as the "Foraging-RL models" to emphasize that they incorporate aspects of both theories. We differentiate these from the "traditional RL models" we consider, which depend on compare-alternatives computations.

Minor comments:

A) It would be very helpful to have a better breakdown of the existing 3 experiments at the start. Specifically, what are the exact tasks and what is manipulated between the experiments and for what purpose. As far as I understood it, Experiment 1 is a simple two option Bandits tasks, experiment 2 has linked probability options and experiment 3 has more options and volatility/uncertainty manipulations?

We have revised the manuscript to more clearly describe the (now) 5 experiments. We have added a concise summary of the motivation for the experiments in the Introduction, expanded the Results sections to provide a thorough description of each experiment and its rationale, and updated (**Figure 5**) with additional panels that break down the design and outcomes of each experiment while highlighting their similarities and differences. We have also included detailed descriptions of the two newly added experiments in the Methods section. Because the manuscript was already at the journal's word limit, we were unable to incorporate an extended overview of all experiments directly into the Introduction; however, we have added this comprehensive overview to the relevant result section.

In the introduction: Therefore, we developed a novel compare-to-threshold sequential decision-making model ("foraging-RL model") that is inspired by foraging theory but incorporates delta-rule reward prediction errors from RL. We compared this new foraging-RL model with multiple variations of the compare-alternatives RL models ("traditional RL") [39] across five independent experiments that manipulated the uncertainty of the unchosen options, the demands of the environment, and the setting in

which the experiment was performed. We found that the foraging-RL model was a better fit for the participants' decisions in all experiments, outperformed multiple variations on traditional RL models, and better predicted the participants' tendency to repeat choices on both individual and group level.

In the results: It remained possible that the results in Experiment 1 were a consequence of the specific environment we chose (i.e., a slow-moving bandit task with two independent option values that was conducted online). We therefore fit the models to 4 additional datasets in which we manipulated (1) the independence of option values (Experiment 2; two options), (2) the volatility of values (Experiment 3, 3-options), (3) the number of options (Experiment 4; 2-, 3-, and 4-options), and (4) the experimental setting (Experiment 5; in person, multiple response modalities). Experiment 2 allowed us to hold richness constant across the task and, along with Experiment 3, to bidirectionally manipulate the uncertainty of the unchosen option(s). Experiments 3 and 4 allowed us to determine how the number of available alternatives influenced model fit. Finally, because Experiments 1–4 were all conducted online, Experiment 5 replicated the original experiment in person, using both oculomotor and touchscreen response modalities.

In the Methods:

Participants

Online Experiments (1-4)

Participants in Experiments 1-4 were recruited via the online platform, Amazon Mechanical (mTurk). To avoid bots and improve data quality, participants were only accepted when they had a minimum of 5000 approved human intelligence tasks (HIT) and a minimal percentage of 98% in proportion of completed tasks that are approved by requesters. Geographical restrictions were set for US participants only. To help ensure that participants understood the task, there was an initial 25 trial block with fixed but randomly assigned reward probabilities (20%, 30% and 70%). To continue to the main task, participants were required to (1) switch between options at least twice, and (2) get reward more than 42% of the time during the practice block. The criteria and number of trials was chosen to ensure that people choosing at random would rarely pass through to the main task. Participants were not allowed to repeat the experiment. All participants who successfully submitted the HIT, either the practice block or the full experiment, were paid a base rate of \$0.50, plus a bonus of \$3.61 mean $\pm$ \$0.97 SD based on their performance (participants were given a \$0.02 compensation for each trial that ended with a reward). All participants completed 300 trials in each experiment. Participants provided informed consent following a full explanation of the experimental procedure that reminded them of their right to withdraw at any time during the study.

For Experiment 1 (2-option bandit task), a total of 258 participants (120 females, 137 males, 1 preferred not to say) completed the task. For Experiment 2 (2-option task with linked rewards), a total of 95 participants (41 females, 51 males) completed the task. For Experiment 3 (3-option bandit task with variable volatility), a total of 270 participants (112 females, 156 males, 2 prefer not say) completed the task. For Experiment 4 (k-option bandit task with variable k), a total of 150 participants (sex and gender was not collected) completed the task. Some analyses of Experiment 1, 4 and 5 are included in a manuscript comparing switching behavior between mice, monkeys, and humans [28], but all analyses presented here are new.

Experiment 1: 2-option restless bandit task. In Experiment 1, participants were required to repeatedly choose one of two options. Each option was associated with a probability of reward, which changed across trials and independently across options (step size = 0.1, hazard rate = 0.1). Each participant's reward schedule was randomly generated to avoid environmental biases.

Experiment 2: Linked 2-option bandit task. To understand how fixing richness and decreasing uncertainty in unchosen option value influences behavior, participants performed a modified version of the two-armed restless bandit task in which option values were linked. One option walked according to the same statistics used in Experiment 1, while the value of the other option was set to 1 minus this value. Because the values always summed to 1, there was no variation in the richness of the environment across trials.

Experiment 3: Volatile 3-option bandit task. To explore how increased uncertainty influences behavior, participants performed a 3-option restless bandit task under multiple different levels of volatility

in the environment. All volatility levels were higher than that used in Experiment 1. Specifically, both hazard rates and step sizes were variable (hazard rates were between 0.1 and 1; step sizes ranged from 0.05 to 0.667). Participants were classified into one of 3 volatility bins based on terciles of the decay of the autocorrelations in the reward walks (trial at which the autocorrelation decayed to 0.75 of its initial value; Figure 5H). We also added an additional option in this task, which has no significant effect on the probabilities of switching or exploring [28], but did allow us to also analyze any dependence of alternative option value on choices (Figure S5).

Experiment 4: Multi-option bandit task. To understand how the number of alternatives options influenced behavior, participants performed either 2-, 3- or 4- option restless bandit task (50 participants in each group). Experiment 4 also used a slightly higher hazard rate than Experiment 1 (0.2) to encourage exploration.

In person experiment (5)

Experiment 5: In-person 2-option bandit task We conducted an in-lab experiment using a 2-option restless bandit task to achieve two goals: (1) determine if compare-to-threshold computations are still preferred in an in-person setting (as opposed to online platforms like Amazon MTurk) and (2) test if different response modalities (eye tracking vs. touchscreen) affected choice of algorithm. The experiment consisted of 20-25 trials practice block to familiarize with the task followed by 2 testing blocks (1 for each modality: eye movements and touch screen presses). While online experiments used playing cards as stimuli, in-person experiments cued option locations with small equiluminant gabors and included small (< 1 degree) central fixations that had to be looked at (in eye modality) or touched (in touch modality) to initiate a trial (after [13]). For eye modality responses, monocular eye data was sampled at 2,000 Hz with an Eyelink 1000+ infrared eye tracker. Saccades were identified in real-time using custom Matlab routines. Touch responses were recorded via an infrared touch frame (Chengying CYS-27) placed on top of the monitor and processed via standard Paychtoolbox-3 routines. The modality assigned to the first block was randomized to counterbalance order effects. Practice trials were excluded from all analyses. Data was collected between 9 AM and 5 PM EST. A total of 29 participants (19 females, 10 males) completed the task. Participants completed an average of 483.28 ± 43.01 (SD) trials in the eye-tracking version, and an average of 499.48 ± 1.70 (SD) in the touchscreen version.

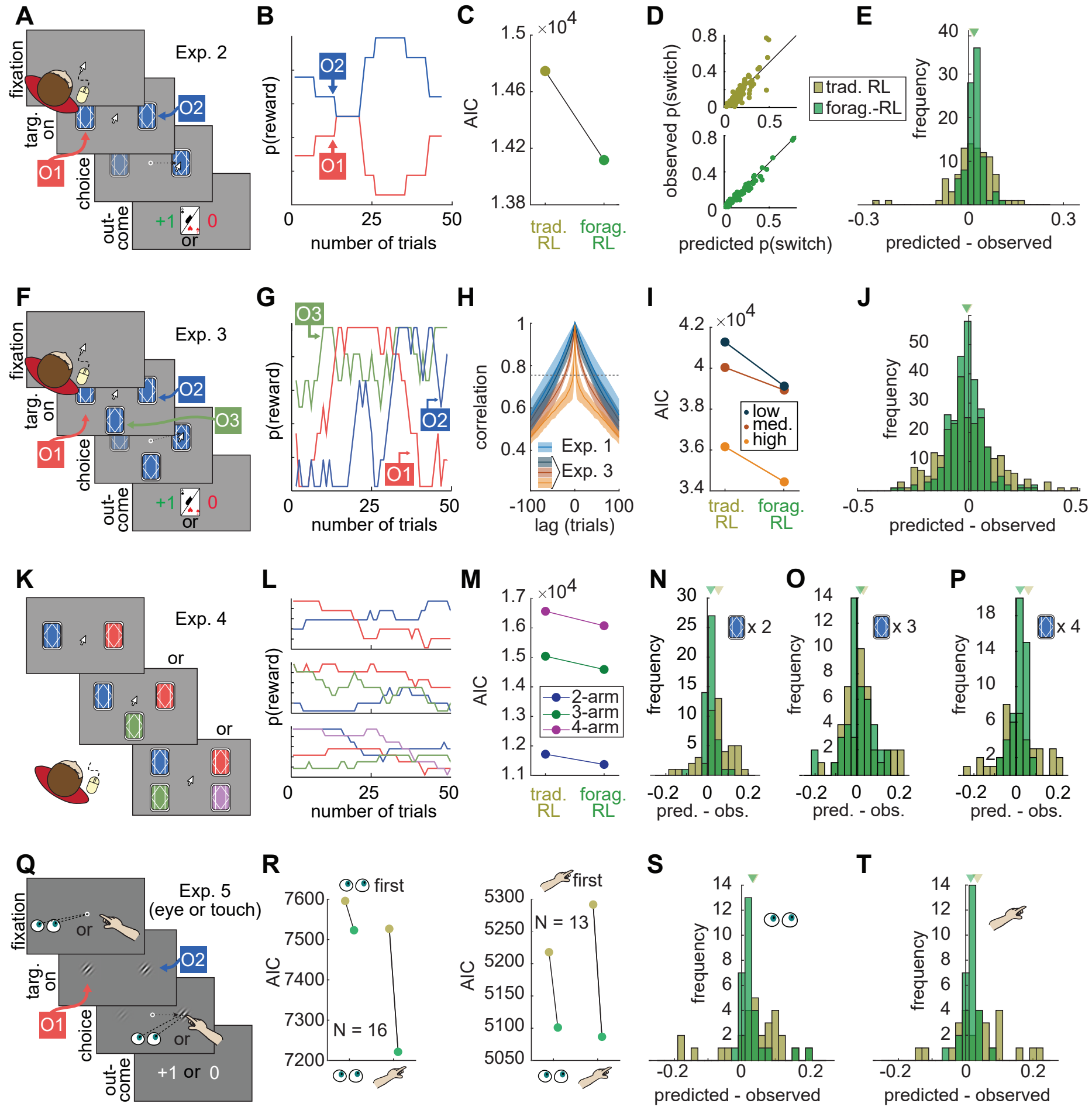

Figure 5: **Foraging-RL better describes participants' behavior across multiple tasks. A)** Schematic representation of Experiment 2. Participants (n = 95) performed a 2-option bandit task in which option values always summed to 1. **B)** Example reward schedule (50-trial portion) from Experiment 2 (red trace = option 1; blue trace = option 2). **C)** The AIC of traditional RL and foraging-RL model fits to all data from Experiment 2. **D)** Participants switch probabilities as a function of predicted probability of switch from simulations of the traditional RL model (*top*), and the foraging-RL model (*bottom*). Dots = participants. **E)** Distribution of the difference in predicted versus observed switch probability for both models. Colors as in D, carets = means. **F)** Experiment 3. Participants (n = 270) performed a volatile 3-option bandit task where changes in option values occurred more rapidly than in Experiments 1 and 2. **G)** An example reward schedule (50-trial portion) experienced by one participant from Experiment 3. **H)** Auto-correlation in reward schedules averaged for all participants of Experiment 1 (*blue*), and the 3 volatility bins in Experiment 3 (*dark purple, red, orange*). Note that even the "low" volatility environment is higher volatility than Experiment 1. Full line indicates the mean, shading indicates STD and dotted line indicates the decay threshold. **I)** The AIC of traditional RL and foraging-RL model fits to all data from Experiment 3. **J)** Same as *E* for Experiment 3. **K)** Experiment 4. Participants (n = 150) performed 2-, 3-, or 4-option bandit tasks (50 participants per condition). **L)** Example reward schedules (50-trial portion) from Experiment 4 for 2- (*top*), 3- (*mid*) and 4- (*bottom*) option conditions. **M)** The AIC of traditional RL and foraging-RL model fits to all data from Experiment 4. **N-P)** Same as *E* for 2- (*N*), 3- (*O*) and 4- (*P*) option conditions. **Q)** Experiment 5. Participants (n = 29) performed two versions of a 2-armed bandit task in person and in randomized order. In the "touch" version, participants physically touched the chosen option on a touchscreen. In the "eye" version, participants saccaded to the desired option and held fixation to select it. **R)** (*left*) The AIC of traditional RL and foraging-RL model fits to data from Experiment 5 for either participants who experienced the eye (*left*) or touch version of the task first (*right*). **S-T)** Distribution of the difference in predicted versus observed switch probability for both models for the eye-tracking modality (*S*) and the touchscreen modality (*T*). caret = mean.

B) Actually, switching a lot at low discriminability levels, isn't necessary utility optimizing as both options are similar in value and therefore the marginal value of picking the better one is low, particularly if one assumes as cost to deliberation, which is also hardest with low value differences.

The reviewer raises a great point—that switching a lot at low discriminability levels is not likely to be a great strategy. We have added the following language to the discussion to highlight the point that this may be one of the structural limitations of a traditional RL / compare-alternatives algorithm and therefore a relative advantage of choosing to use compare-to-threshold computations in these kinds of environments:

Discussion : Further, we noted here that compare-alternatives computations also predict that people should explore the most when discriminability is low and values are most confusable: exactly when the marginal utility of choosing another option is actually the worst (Figure 2A). Considering that exploratory choices are both costly and time-consuming [5, 14, 13, 36, 24], compare-to-threshold choices offer the additional advantage of exploring only when they offer the biggest potential return—when a previously exploited option begins to disappoint and the search for an alternative is truly warranted. In short, it seems likely that humans favor compare-to-threshold computations because this approach is more computationally efficient, better aligned with the brain's biological constraints, and better suited to the scale and statistics of natural environments.

C) For the first experiment switching to "random" is same as switching to the only other option, no? If so that should be clearer.

Yes, that is correct, we model exploration as a switch away from an exploited option to a random alternative, which in the case of a 2-alternative task is just a switch to the only alternative. We have made this explicit in both the results and methods:

In Results : Where a traditional compare-alternatives RL agent estimates the value of choosing each option and compares them ($V_1, \ldots, V_n$; Figure 3A), our novel foraging-RL agent updates the value of exploiting the last chosen action via the same delta-rule computations used in traditional RL agents (V_{exploit}; Figure 3B). It then compares this value against a fixed threshold to decide whether to continue exploiting or to explore the alternatives at random (*foraging-RL model*; Figure 3B). **Where there are only 2 options, this means deterministically switching to the only alternative.**

And in the Methods : In the foraging-RL models, values do not represent the value of a single focal option. Instead, the only value tracked in the model is the value of staying with or "exploiting" the option that was just chosen (...) and in: It is important to note that the foraging-RL algorithm only tracks one value, the value of the exploited option. Whenever the model switches to a new primitive action (explores), the value of exploiting that action is unknown, so the value of exploitation is reset to the threshold. In this way, the value of exploitation always represents the value of terminating a sequences of choices to a particular option, even if the model has only just begun choosing that primitive action.

D) Similar to A, but if the authors don't want to change the overall structure of how the present the experiments (which I leave up to them), they should at the very least improve how the illustrate the Exp 2 and 3 to make it clearer what they look like and what is being manipulated schedule wise.

As described above (in response to major point 3 and minor point A), we have revised the text and Figure5 to better illustrate the logic of and differences between experiments.

2 Reviewer #2 (and #3)

In this paper, Zid and colleagues investigate whether human sequential choices under uncertainty with partial information (i.e., only about the selected option, creating an explore-exploit tradeoff) are more similar to computations that compare option values to make a choice ("compare-alternatives") or computations that compare only the value of a currently selected option to a fixed switching threshold ("compare-to-threshold"). They report evidence that compare-to-threshold models better capture subjects' choices in non-stationary (drifting) 2- and 3-armed bandits, and provide a better fit to detailed aspects of the temporal dynamics of subjects' repetition and switching. The authors interpret these results as indicative of "foraging computations" and suggest that this has broader

implications for models of decision making under uncertainty. We broadly agree with the aims and importance of the article. Indeed, there are long-standing puzzles about the nature and causes of human decision-making biases in general, and biases in reward-based sequential decision tasks in particular, relative to standard (compare-alternatives) formulations of utility-theoretic models. Despite much research on such biases, and despite their likely theoretical importance, conclusive answers remain incomplete, and the direction taken by the authors is a promising one. Overall, we find the study's methodology to be sound and rigorous, and the paper to be clear and well argued. To name a few aspects of the study that we appreciate: first, the framing of decisions in bandit problems in terms of foraging theory is, to our knowledge, a rather novel contribution that helps relate foraging frameworks to a large body of work in psychology and neuroscience. Second, the finding that compare-to-threshold models perform worse on task than compare-alternatives, but provide a better fit to subjects' choices, is indeed intriguing and suggestive. Third, the model comparison and the sequence of model extensions are mostly convincing. Finally, the detailed analysis of repetition patterns in terms of multiple decay rates of choice history effects and relating these to latent explore-exploit states helps to make sense of why the compare-to-threshold models provide a better fit to subjects and situates the study as a natural and important extension of the lab's previous work. Nevertheless, we find important but remediable shortcomings in the currently reported results, which are currently inconclusive with respect to the main research questions and limit the impact of the paper. We also believe that the framing of the study can be sharpened to clarify the paper's contributions. We summarize our main criticisms and suggestions as follows.

We appreciate the reviewers' kind words about the novelty, rigor, and potential impact of this work as well as their detailed feedback and insightful questions. We have made a number of changes to the manuscript to address these concerns, including clarifying our text and revising our statistical approach in places, which we will detail below.

Specific additional analyses include (1) a Generalized linear model (GLM) to estimate the weight of chosen and unchosen options on behavior from Experiment 1, (2) an analysis of the stability of the fits from Experiment 1 to assess whether there was any strategy change, (3) an analysis of alternative option value on switching behavior from Experiment 3, (4) fitting both traditional RL and foraging-RL to 2 additional dataset one of which was collected in person, (5) developing an additional extension to traditional RL that integrates a repetition bonus to the softmax and fit it to data from Experiment 1, (6) fitting the 3 models ([19, 35]) that integrate a prior about the richness of the environment suggested by the reviewer, and (7) including the 1-parameters WSLS as a baseline comparison for both the fits and the simulations. New figures include (1) Figure 1F-G, (2) Supplementary Figure S2, (3) Supplementary Figure S3, (4) Supplementary Figure S4, (5) much of Figure 5, and (6) Supplementary Figure S5. We believe that these changes have significantly improved the manuscript, we thank the reviewers for their time and insight and hope we have addressed their concerns.

[1] It appears that some very direct tests for potential rejection of the compare-to-threshold model are not included. A core assumption of this model is that the probability to repeat/switch is not sensitive to the value of the other (=switch) option(s), but only to the repeat option (which is compared to some threshold). A very direct test of this assumption in the 2-armed bandit case would be to run a logistic regression of choices, with the decision to repeat as the dependent variable and as regressors a constant, the value of the switch option, and the repeat option. The compare-to-threshold model predicts no effect of the switch option value, while the compare-alternative model predicts a negative effect. Note that there are several ways to perform this analysis: using the generative reward probabilities for the option value, or using model-based estimation, e.g., with a delta-rule model, with an ideal observer model, etc.

The logistic GLM suggestion is related to major comment #1 from Reviewer 2 and 3. In order to address these comments we used a GLM to independently estimate the contributions of the chosen and unchosen option values to choice. If, as the reviewer suggests, the difference in value is calculated, then we should see positive beta weights for chosen options and negative beta weights for unchosen options (producing a difference in values). This was true in a very small minority of participants (3 / 258 had opposite signs for Value A and B, to use the reviewer's terms).

The idea that foraging would predict positive signs for both Value A and B is, we believe, a misunderstanding related the lack of clarity in our original description of the fingerprint analyses (this has been

corrected here, see below). The compare-to-threshold approach does not calculate or operate on richness, it only calculates the value of the focal option. So, in the reviewer's terms, the specific prediction this view would make is that Value A would be encoded, but Value B would not. Indeed, that's what we see in 115 / 258 participants: positive beta weights for chosen options and beta weights near zero for unchosen options. Further, model comparison did not suggest that including a term for unchosen option values was necessary.

Changes to the manuscript to describe the new logistic model are as follows:

Methods:

Choice predictive Generalized Linear Model (GLM)

To determine whether the value of previously unchosen options influenced choice, we used logistic regression to predict the probability of repeating choice on a trial-by-trial basis for each participant. We defined the log odds of $p(\text{repeat}_t)$ as

$$log\left(\frac{p(\text{repeat}_t)}{1-p(\text{repeat}_t)}\right) = \beta_0 + \beta_1 V(\text{chosen}_{t-1}) + \beta_2 V(\text{unchosen}_{t-1}) \ , \tag{3}$$

where $V(\cdot)$ is the objective value of the option that was chosen or unchosen at time $t-1$. In this formulation, decisions that depend on comparing values would have positive beta weights for $V(\text{chosen}_{t-1})$ and negative beta weights for $V(\text{unchosen}_{t-1})$, indicating that the probability of choice is proportional to $V(\text{chosen}_{t-1}) - V(\text{unchosen}_{t-1})$. Conversely, decisions that depend on comparing to a threshold would only have positive beta weights for $V(\text{chosen}_{t-1})$, because only the focal option value is tracked.

Objective values were used because these offered the fairest comparison between views. We could not estimate subjective values without making an assumption about whether the participants are using compare-alternatives or compare-to-threshold computations. Because models were fit on a trial-by-trial basis, $p(\text{repeat})$ was a binary vector labeled as 1 whenever the choice on the trial t was the same as the choice on trial $t-1$.

To estimate the relative contribution of each predictor, we also fit two reduced models: one containing only $V(\text{chosen}_{t-1})$ and another containing only $V(\text{unchosen}_{t-1})$. In Figure 1F-G, the p-values for the coefficients of interest ($V(\text{chosen}_{t-1})$ and $V(\text{unchosen}_{t-1})$) obtained from the full model were adjusted using a Holm-Bonferroni correction within each predictor. Model comparison was evaluated with the Bayesian Information Criterion because we found that the Akaike Information Criterion had an insufficient penalty for the number of parameters (i.e., the full model was generally preferred even when option values were completely randomized). Participants who never switched were excluded from this analysis because model parameters were not identifiable.

Then, a new result section was added to the revised manuscript:

Participants largely ignored the value of the unchosen option

The compare-to-threshold view predicts that the probability of choosing an option is only determined by the prior value of that option, not the value of any alternative options. Therefore, choices should be entirely insensitive to the value of unchosen alternatives. By contrast, decisions should depend on the difference between chosen and unchosen values in the compare-alternatives view. To determine which view better described participants' choices here, we developed a generalized linear model (GLM) that predicted choice from the reward probabilities of the previously chosen option ($V(\text{chosen}_{t-1})$) and the previously unchosen option ($V(\text{unchosen}_{t-1})$; see Methods). We found that the value of the chosen option was a strong positive predictor of the decision to repeat a choice (Figure 1F, *left* : sig. positive in 118 / 254 participants). However, the value of the unchosen option had minimal influence (Figure 1F, *right* : sig. positive in 2 / 254 participants; sig. negative in 11 / 254 participants). Further, of the 11 participants who had a negative $V(\text{unchosen}_{t-1})$ term, only 3 also had a significant positive $V(\text{chosen}_{t-1})$ term. This meant that only 3 out of 258 participants (1.2%) were value "comparators" (Figure 1F, *right*) in the sense predicted by the compare-alternatives view, and the vast majority of participants (98.8%) were not. Model comparison revealed that a model which included only $V(\text{chosen}_{t-1})$ outperformed

a full model (containing both $V(\text{chosen}_{t-1})$ and $V(\text{unchosen}_{t-1})$) (Figure 1G). The $V(\text{chosen}_{t-1})$-only model was also better fit to the majority of individual participants (better fit in 174 / 254 participants; $wBIC$ of full and $V(\text{chosen}_{t-1})$-only models relative to $V(\text{unchosen}_{t-1})$-only model (both < 0.0001)). Together these results suggest that the value of the previously chosen option was the primary driver of choice, with minimal influence from unchosen option values, consistent with the compare-to-threshold view.

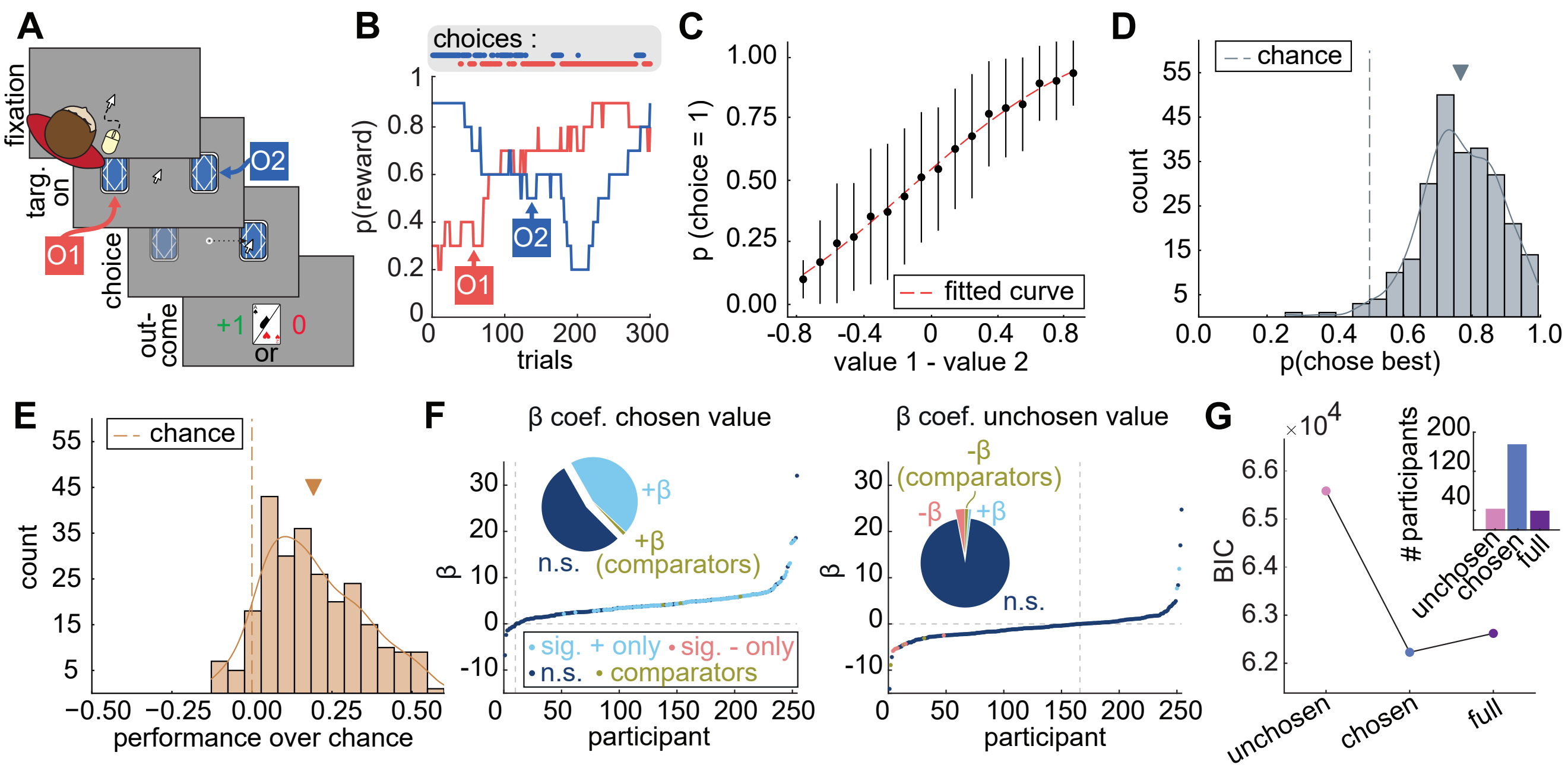

Figure 1: **Experiment 1 task and baseline behavior. A)** Participants (n = 258) chose between 2 face-down decks of playing cards that were composed of aces and 2's. Participants were told that the proportion of aces and 2's in each deck would change over time and that decks could be good, bad, or mediocre right now, but would not stay that way the whole time. Participants got a point (and \$0.02) for each ace they found, but no points for 2's. **B)** The reward probability of each option (red trace = option 1; blue trace = option from an example reward schedule, with participant choices along the top (red dots = option 1; blue dots = option 2). **C)** Average probability of choosing option 1 as a function of the difference in the objective reward probability ("value") between option 1 and 2. Error bars = standard error of the mean (SEM) across participants. **D)** Distribution of the number of trials in which each participant chose the objectively best option. Dotted line = chance, caret = mean across participants. **E)** Same as *D*, for the percent of trials in which participants were rewarded, normalized as percent of chance performance. **F)** Predicting choice from the value of chosen and unchosen options. Beta coefficients for ($V(\text{chosen}_{t-1})$) (*left*) and $V(\text{unchosen}_{t-1})$ (*right*). Each circle represents a single participant, sorted by coefficient value. The dashed horizontal line is at zero (no effect). Participants with a significant positive betas are light blue, significant negative betas in red, and non-significant betas in dark blue. Gold circles identify "comparators" who had a significant positive weight for the chosen option and negative weight for unchosen option value. Significance is Holm-Bonferroni corrected within predictor ($\alpha = 0.05$). Insets illustrate the proportion of participants in whom each term was significant, colored by sign and identifying all "comparators". **G)** sum of BIC for each model ($V(\text{unchosen}_{t-1})$-only, $V(\text{chosen}_{t-1})$-only, and the full model which contains both terms). Inset) The number of individual participants best fit by each model, based on the Bayesian Information Criterion (BIC).

Another very direct test of this assumption in the 3-armed bandit case is that when a switch occurs, the choice probability should be uniform among the other two alternatives. In contrast, the compare-alternatives models predict that this probability should reflect the reward probability of those alternatives. The authors conducted a 3-armed bandit task in the experiment, which is a strength of the paper, so testing this prediction is only a matter of additional analysis.

This is another great suggestion that was also made by Reviewer 1. We analyzed the effects of alternative option value in data from Experiment 3 and the results did not change our overarching conclusion. These

analyses have been added as Supplementary Figure S5 and via multiple changes to the text. Briefly, the majority of participants were not influenced by alternative option values, but the majority of those who were tended to chose the worst alternative–not the best. This is difficult to reconcile with any traditional RL model that we are familiar with. By contrast, we believe that a foraging model could account for all these observations if it had the working memory capacity to track (1) the identity of the last exploited option, (2) the identity of the most uncertain (least recently selected) option—important directions for future model development. This is described in the text as follows:

Results : Because 3 targets were used in Experiment 3, we could also leverage this dataset to determine how the value of alternatives influenced choice. In a 3-alternative setting, compare-alternatives computations would be more likely to switch to the best alternative option where compare-to-threshold computations would explore uniformly because alternative values are not tracked. During exploratory switching (Figure S5A-B; see Methods), a minority of participants had some bias between the two alternative options. However, the majority of these chose the worst alternative option, not the best. By contrast, traditional RL models would predict a large number of individuals with a bias towards the best alternative, not the small number seen in practice (i.e., 2.4%-7.8%, 5-18 people / 270, depending on method; 2.5% expected by chance). However, the fact that 35-46 participants had any bias at all was also not consistent with the assumption that exploration is random and that unchosen option values are forgotten instantaneously in the foraging-RL models. This was especially apparent in switch-backs—decisions that revert to a previously exploited option (compare Figure S5C and D)—implying that adding some encoding of the last exploited option's identity could improve the foraging model substantially. Similarly, adding some capacity to preferentially explore options that have not been recently sampled would likely both improve the foraging model and account for the results in Figure 4G and S5A, B, and D) because these options are statistically more likely to be lower value than those that the model has recently chosen, given that the model tends to choose good options (Figure 3C-F). In short, while a minority of participants exhibited biases between alternative options in the 3-option case, rectifying these observations with the foraging model could be accomplished through adding a working memory component that recalls (1) the last exploited option, and (2) the most uncertain option, without tracking alternative values.

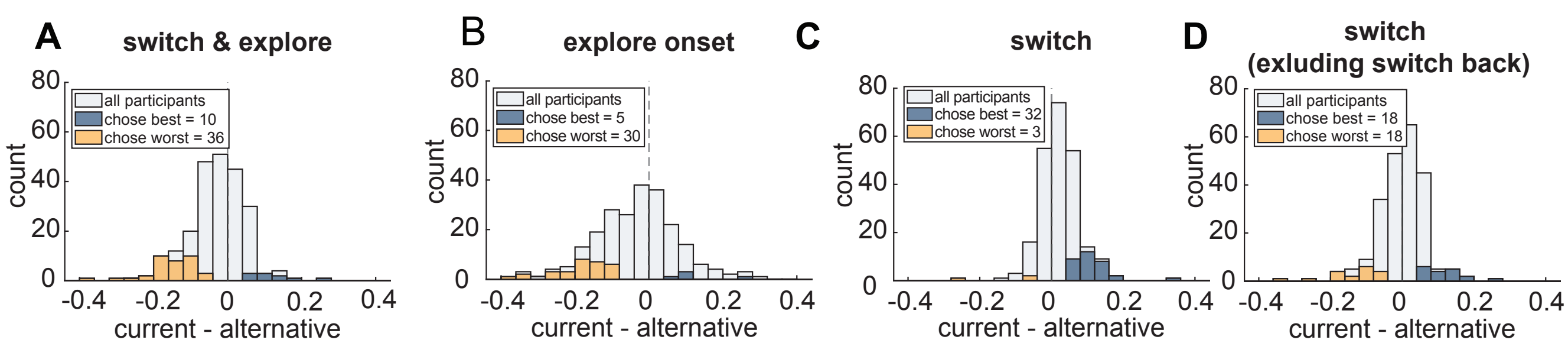

Figure S5: **Switching preferences among the alternative options in Experiment 3 A)** Because 3 options were used in Experiment 3, we were able to ask if participants preferentially switched to the best or worst of the alternatives. We first focused on exploratory switches: switch decisions that were also identified as exploratory via the HMM (see Methods). Distributions (*grey*) show the average difference in objective option values for these decisions within each participant. Positive values indicate participants who switched to the objectively better alternative, negative values indicate participants who switched to the objectively worse alternative. Participants that chose the best alternative significantly more frequently than chance are in blue. Participants that chose the worst alternative more frequently than chance are in orange. Chance was estimated by running 50,000 bootstrap simulations of random choices for each individual, significance tested at $p < 0.05$, uncorrected. **B)** Same as *A*, but specifically for the first explore trial in a sequence ("onset" of exploration) which is a switch decision by definition. **C)** Same as *A*, but for all switches, many of which are "switch backs", returning to the previously exploited option. **D)** Same as *C*, but excluding the switch backs to the last exploited option.

[2] To rule out the compare-alternatives model requires ruling out the entire class of RL models. This is not easy because it requires testing many variants of the RL model. The authors made the commendable effort to try several alternatives. We are not aware of any method that would

guarantee that all possible variants have been tested. We make the more modest suggestion that two more variants be included. (i) The models appear to differ in their ability to capture subjects' repetition behavior. A simple extension of the RL model (and possibly the food model) is to include a repetition bonus in the softmax. In the RL model, this would correspond to a dummy regressor indicating whether option a-i was selected in the previous trial (in the case of the 2-armed bandit, it is also possible to reparametrize the model into choice repetition versus switch and include a constant). The history kernels come close, but in more sophisticated forms that do not capture the simplest form of repetition bias.

We appreciate reviewer's point that a limited number of additional models would be helpful and we have added 8 key models to the revised manuscript. To address this specific point, we have added the +1 parameter repetition bonus to the compare-alternatives RL model, which offers a nice compliment to the 2 choice-history dependent models in the original manuscript, but again did not outperform the foraging model.

We describe our specific implementation of this new model in the Methods: Because there were significant computational differences between how choice-history dependence functioned in the foraging-RL model, compared to traditional RL, we also considered a 1-parameter "repetition bonus" extension of a traditional RL model, in which some probability of repeating the immediate last choice was added into the softmax decision rule as

$$p(\text{choice}_t = i) = \frac{e^{\beta((1-b)v_{i,t}+bc_{i,t})}}{\sum_{j=1}^{N} e^{\beta((1-b)v_{i,t}+bc_{i,t})}} , \tag{4}$$

where $c_{i,t}$ is a dummy variable indicating whether or not option i was chosen on the last trial (1 = chosen). b constrained as $0 < b < 1$, is a repetition bonus parameter that controls the relative weight of choice repetition in the model. Because value is calculated using reward prediction errors, $0 \leq v_{i,t} \leq 1$ and $0 \leq v_{i,t} \leq 1$, $b = 1$ implies that the last choice is weighed as much as value, whereas $b = 0$ implies that value dominates the last choice completely.

Model fits are described in the results: A variety of extensions and alternatives are known to outperform the standard 2-parameter traditional RL model, and it remained unclear whether any of these would fit behavior better than the foraging-RL model. Therefore, we next compared the foraging-RL model with 10 additional models: 4 common extensions of the traditional RL model (asymmetrical learning, value decay, and 2 forms of choice history dependence), 2 dynamic Bayesian models and a free-fitting exponential filter (see Supplementary Figure S3E), and **3 extensions meant to address the specific limitations of the traditional RL model identified here (see Methods). At the group level, every extended traditional RL model explained behavior better than the standard traditional RL model (Figure 3J-K, Supplementary Figure S3). The foraging-RL was a better fit than all but 1 traditional RL model (Figure 3J-K, Supplementary Figure S3, Supplementary Table 1).** (...) **Foraging-RL also outperformed 3 extensions to RL models that were developed to account for our behavioral observations**: an RL model in which values are explicitly normalized by richness (see Methods; "richness"; Supplementary Figure S3E; Supplementary Table 1), **a repetition bonus model that implements a tradeoff between repeating the last option and making decisions based on relative value (see Methods; "repetition bonus"; Supplementary Figure S3E; Supplementary Table 1)**, (...)

And shown in supplemental Figure S3E.

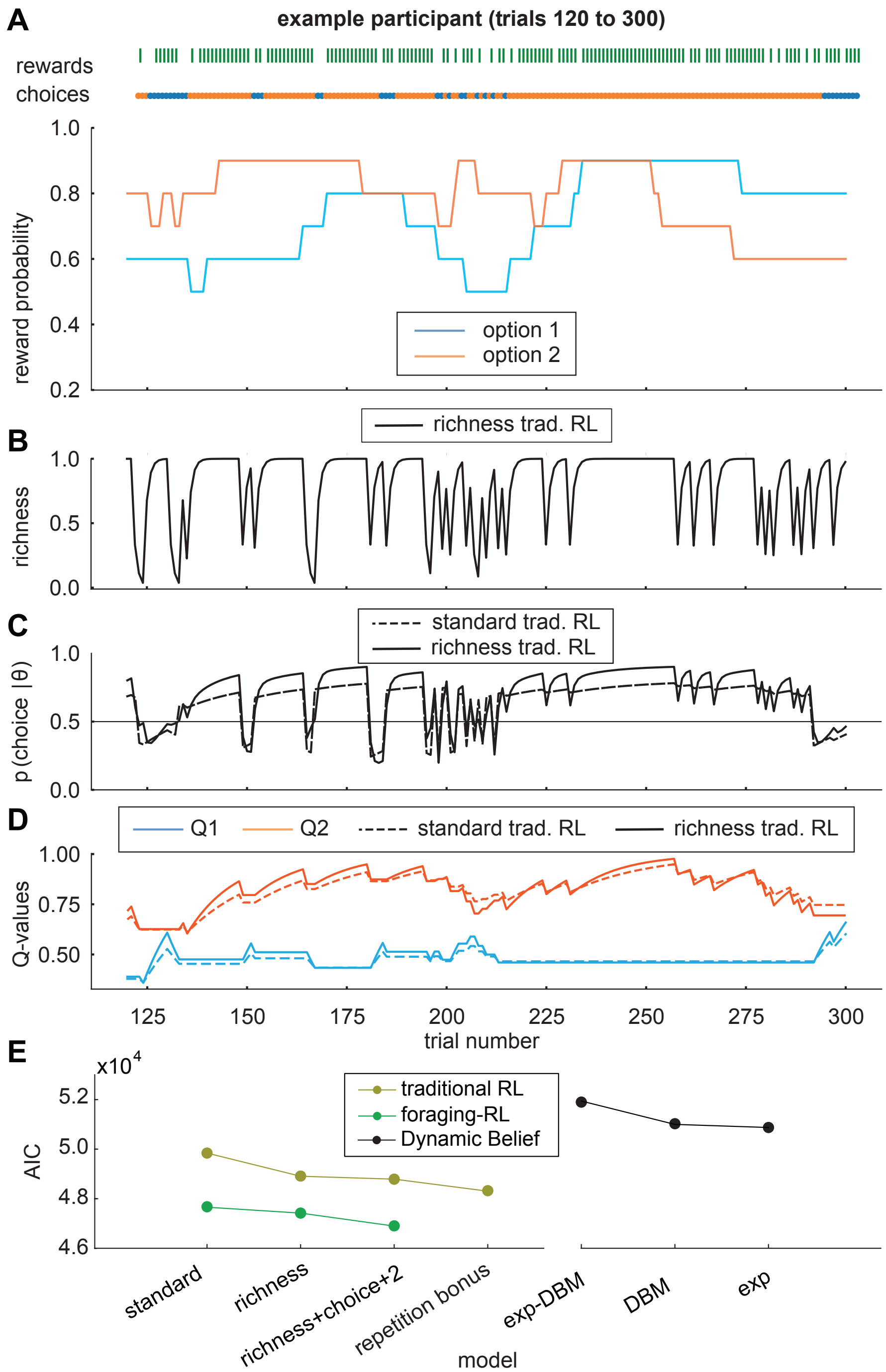

Figure S3: **Additional model fits. A)** Example reward probabilities from a single participant in Experiment 1. Trials 120 to 300 are shown for illustration. Color of the circles on top correspond to the option chosen by the participant. Green ticks indicate that the participant received reward on that trial, and red ticks indicate omission of reward. **B)** Richness, as estimated by the richness traditional RL model, with maximum-likelihood parameters that best explain the tracking of richness under this model. **C)** The likelihood of fitted standard traditional RL (standard trad. RL; dotted) and richness traditional RL (richness trad. RL; solid) models for each choice made by the participant. Chance at 0.5. Richness trad. RL has a better likelihood because the richness allows this model to have a more deterministic choice than standard-RL, even with similar Q-values. **D)** Q-values calculated by each model (see Methods). **E, left)** AIC values for additional extensions of traditional RL and foraging-RL models, fits to behavior from Experiment 1. "Richness choice" models incorporate both choice history dependence and richness terms (see Methods). **E, right)** Same for additional compare-alternative models from a dynamic belief class. exp-DBM is the exponential filtering approximation to the Dynamic belief model ([35], see Methods), DBM is the Dynamic Belief model as in [19] and exp is a fully fitted exponential filter ([35].

	model	total parameters (k)	AIC	log-likelihood	AIC_w
**traditional RL**	repetition bonus	762	48323.8	-23,399.9	< 0.0001
	richness	1016	48,913.3	-23,440.7	< 0.0001
	richness and history kernel 2	1524	48,787.7	-22,869.8	< 0.0001
**dynamic belief**	exp-DBM	508	53,150	-26,067	< 0.0001
	DBM	508	51,025.9	-25,004	< 0.0001
	exp	1016	50,873	-24,420	< 0.0001

Table 1: **Pairwise, group-level model comparison against standard foraging-RL for Experiment 1**. AIC weights are computed relative to standard foraging-RL (AIC_w for standard foraging-RL was 1 in all pairwise comparisons).

(ii) In the compare-to-threshold model, the threshold can be interpreted as a prior about the richness of the environment. Such a prior is not included in the RL model, but it is possible to do so, see for example the work of Angela Yu. In her learning model, the estimation of the option value includes a prior about the expected value of options in general (thus capturing an optimism/pessimism bias of the participant). This prior is persistent and recurring in the value update, is different from the initial value of the delta rule, and actually requires extending the delta rule into a slightly more complex learning model.

references

Guo, D, Yu, AJ (2018). Why so gloomy? A Bayesian explanation of human pessimism bias in the multi-armed bandit task. Advances in Neural Information Processing Systems, 32.
Ryali, C, Gautaum, R, Yu, AJ (2018). Demystifying excessively volatile human learning: A Bayesian persistent prior and a neural approximation. Advances in Neural Information Processing Systems, 32.

We fit both versions of the Dynamic Belief Model referenced here, but the model did not perform well on this dataset so the results did not alter the major conclusions. The Guo et al. paper develops and applies the Dynamic Belief Model (DBM) to static multi-armed bandits, and the Ryali et al. paper compares a tractable approximation of the full DBM with an exponential filter model in a visual search task. Here, we fit the full model from Guo et al. (2018), the approximate DBM from Ryali et al. (2018), as well as a fully fitted exponential filter from Ryali et al (2018). We found that all of these models did a poorer job at fitting our data than any of the other models we fit (new supplemental Figure S3E). Our intuition is that our environments were incompatible with the number of changes assumed in these models, but it would be interesting in future work to understand more about why this approach does not seem to be a good fit here, despite performing better than some RL algorithms in other datasets. We have added the following language to detail our implementation of the Dynamic Belief model:

in the Methods:

Dynamic Belief Model Another way of solving a restless multi-armed bandit is to track a variable that signals if there has been a sudden change in the environment. One particular instantiation of this partially-observable modeling is to track a belief about the environment that changes over time, depending on the outcomes received, called The Dynamic Belief Model (DBM)[19, 35]. DBM is a hidden Markov model with latent variable γ which denotes the probability of observing $x = 1$. It assumes the observations are drawn from a Bernoulli distribution whose rate parameter undergoes unsignaled changes with probability $1 - \alpha$ at each time step. Calculating the predictive probability that one will obtain a success given a past history of observations $P_{t+1} \equiv P(x_{t+1} = 1|x_{1:t})$ entails marginalizing over every possible timing of the most recent change point, or discretizing the belief space. To fit this model on our data, we followed the standard approach of discretizing the belief space (distribution over γ), and using a uniform prior distribution [19].

The standard DBM suffers from being computationally expensive due to the implicit marginalization of the probability distributions. Another possibility is to use an exponential filter approximation which works up to order α^2 [35],

$$P_{t+1} \approx (1 - \alpha)P_0 + \alpha(Gx_t + P_t(1 - G)), \tag{5}$$

where P_0 is the initial probability of observing a reward, here chosen to be equal to 1/2 for all arms, and $G = \frac{1}{a+b+1}$, with a and b the parameters of the Beta distribution that governs the prior distribution of γ, the probability of observing a success. A sensible choice for these parameters would be a uniform distribution of γ, thus $a = b = 1$ [35].

With this predictive probability, one can build an algorithm that maximizes future reward, through a decision rule like a softmax over this predictive probability for each arm:

$$P(\text{choosing arm}) = \frac{\exp(\beta P_{t+1}(\text{arm}))}{\sum_{\text{arm}} \exp(\beta P_{t+1}(\text{arm}))}.$$

Both the full DBM and its exponential-filter approximation only have 2 free parameters, the probability of the environment changing α, and the inverse temperature parameter β.

A different approach is to use a full exponential filter on this predictive probability P_t, that linearly sums past observations, while exponentially discounting into the past.

$$P_{t+1} = C(1-\delta) + \eta\delta x_t + \delta P_t,$$

where the free parameters (C, η, δ) are constrained as $0 \leq C, \eta \leq 1, 0 \leq \delta < 1, C + \frac{\eta\delta}{1-\delta} < 1$ [35]. Along with the inverse temperature β, this model has thus 4 free parameters.

In the Results: Therefore, we next compared the foraging-RL model with 10 additional models: 4 common extensions of the traditional RL model (asymmetrical learning, value decay, and 2 forms of choice history dependence), **2 dynamic Bayesian models and a free-fitting exponential filter (see Supplementary Figure S3E),** and 3 extensions meant to address the specific limitations of the traditional RL model identified here (see Methods). At the group level, every extended traditional RL model explained behavior better than the standard traditional RL model (Figure 3J-K, Supplementary Figure S3). **The foraging-RL was a better fit than all but 1 traditional RL model (Figure 3J-K, Supplementary Figure S3, Supplementary Table 1)**.

[3] There are problems with model comparison: (i) The baseline model for compare-to-threshold has 3 free parameters (threshold, softmax temperature, and learning rate), and the compare-alternatives model has 2 free parameters (softmax temperature and learning rate). It is generally misleading to report log-likelihood as the primary metric for models that have different numbers of free parameters (e.g., reported in the first place, l. 170; in Fig. 5; used for the foraging index). Consider replacing it with a cross-validated estimate of the log-likelihood, or reporting it as a secondary metric after a metric that accounts for the difference in the number of parameters (e.g., AIC, as used in the paper).

We have revised the text and figures to report either the AIC or the likelihood ratio first (where applicable), followed by the raw log-likelihoods. Because our interpretations were based on AIC in the original manuscript, this did not change our conclusions. We have kept the log-likelihoods in the paper because the raw value offer a numerical sense of how well each model accounts for individual choices, whereas AIC represents an additional transformation that is less intuitively tied to the underlying choice probabilities. While log-likelihoods were used to compute the foraging index in Fig. 5, we had already used AIC values to determine which model was better at both the group and individual levels. The foraging index was only ever used to measure individual differences in relative model fit. While we agree that raw values of this index might be misinterpreted because there is no correction for the number of parameters here, these raw values were not included in the text to avoid just such misinterpretation. An extra parameter in one class would cause a DC offset in the index with respect to 1, but it would not disrupt the relative order of the participants, which was the focus of this analysis. The entirety of the results related to this index are as follows:

In the results: However, there was no systematic relationship between how foraging-like an individual participant was and how well they did in the task (see Methods; no correlation between the "foraging index" and the probability of choosing the best option [$R = -0.01, p = 0.84$] or above-chance reward probability [$R = 0.03, p = 0.64$]).

(ii) The comparison at the group level is based on a fixed effect. Random effects analysis for subjects in a group is more powerful (see e.g. Stephan et al; note that it is not the only methods for random effects analysis, we do not ask the authors to use this one in particular, although it would address our point).

We have updated the manuscript to include many analyses that model subjects as random-effects. These analyses have strengthened our overarching conclusions and improved the quality of the manuscript. Most notably, we now apply random-effects analysis to (1) show that the standard foraging-RL model outperforms standard traditional RL in individual subjects in Experiment 1, (2) to show that the best class of models for each participant in Experiment 1 is the foraging class, and (3) to show that foraging-RL outperforms traditional RL on an individual basis in 3 additional experiments. Additionally, throughout the paper we are explicit about whether conclusions are drawn at the group level or the individual level.

(iii) For the individual-level model comparison (l. 173-177), the metric used seems to be the log-likelihood rather than AIC, which is not appropriate in this case given the difference in model complexity. It may be a typo, with log-likelihood instead of AIC, since the reported value is positive (which is impossible for log-likelihood).

This was indeed a typo that has been corrected in the revised manuscript. We now report both AIC and log-likelihood.

[4] How is a (stationary) threshold learned or calibrated in a particular decision context? Although the authors note that thresholds are assumed to be learned over long periods of time, they cannot be invariant to aspects of the decision context if compare-to-threshold is to be a viable model of actual animal decision making. Again, this may be out of scope, but the current wording of the paper seems overly simplistic: it would be helpful if the discussion commented on how such thresholds might actually be set.

We appreciate the reviewers' point that it's helpful to explain why treat the threshold as a fixed parameter. However, we disagree with the assertion that thresholds "cannot be invariant to aspects of the decision context if compare-to-threshold is to be a viable model of actual animal decision making."

In formal ethological foraging theory, patch leaving thresholds are indeed related to the background rate of reward. However, this relationship is normative, not descriptive. In practice, many foragers under- or over-harvest with respect to the theoretical optimum. The fact that these foragers are nonetheless alive and well suggests that reasonable behavior is possible even when thresholds are imperfectly adjusted to the statistics of the environment.

Further, in the specific foraging-RL model we develop here, it may be more instructive to think of the threshold as a kind of explore/exploit bias parameter, rather than as an online estimate of the average reward in the environment. In related work, we have explored versions of the model where the threshold is learned, but find that these consistently underperform the fixed-parameter version in the kinds of short, single-session tasks used here. If these thresholds are learned, we suspect they are learned on timescales long enough to require a different experimental approach. For now, we believe that it is most appropriate to treat the threshold as a parameter that can vary between or within individuals just as exploration itself varies between and within individuals.

We have made several changes to the manuscript to be more clear about how the threshold parameter functions in the foraging-RL model and how we believe it should be interpreted in this context.

In the Methods : (...) and the threshold ρ, constrained as $0 < \rho < 2$, could be thought of as a threshold for exploration, which, by analogy to foraging theory [37, 4], could reflect expectations about the background rate of reward or value of alternative options in the environment.

Discussion : One place in which our foraging model deviates from the ethological literature is in the threshold parameter. In foraging theory's patch leaving models ([37, 4]), the threshold parameter is determined by the background rate of reward in the environment. Further, previous compare-to-threshold learning models implemented a threshold which dynamically updates itself to estimate this background rate of reward [7]. By contrast, we decided to model the threshold as an individual difference parameter that simply controls the rate of exploration. We did this because the link between the threshold and the background rate of reward is purely theoretical. While this is the normative way to identify the optimal time points at which a forager *should* leave a decaying patch, this assumption does not consistently describe the decisions of real-world foragers, who tend to systematically under-or-over harvest with respect to average-reward thresholds([22, 7, 20, 21, 3, 25, 27]. Modeling the threshold as a fixed individual difference parameter resonates with these results, but also with prior observations that people

have long-term and stable preferences in the amount they explore (e.g., [6]). We know that the frequency of exploration changes with individual differences in personality characteristics and life circumstances that are not related to the rate of reward in any given task [17, 2, 5, 26, 38, 31, 12, 1], and we reported here that the threshold was far and away the most stable parameter across repetitions of the task in Experiment 5. Ultimately, it is probably best to conceptualize foraging-RL threshold as a parameter that integrates multiple sources of information such as previous reward history, inherent beliefs about environments, personality characteristics, and/or unmeasured variability in mood or arousal. Future work is needed to understand how these factors shape the threshold for exploration and over what time scales it may change.

[5] Isn't the most plausible model a hybrid between compare-alternatives and compare-to-threshold? (and possibly other strategies). It is known that people can of course explicitly compare alternatives, and it would be quite extraordinary if they never or rarely do so in reward-guided sequential decision tasks under uncertainty. The space of latent choice-generating states plausibly includes threshold-comparison and alternative-comparison states (e.g., as a further hierarchical level). For example, in the present study, there may be stronger evidence for compare-alternatives early in the task (when a threshold may not have stabilized), which is gradually overtaken by compare-to-threshold, which in turn may give way to compare-alternatives in instances of apparently large changes in the environment. Including this would be an important contribution, in our view, because it would (a) be more faithful to the rich structure of actual decision making, where a number of non-mutually exclusive mechanisms are likely to be at play, and (b) be informative for algorithmic accounts of how compare-to-threshold might be implemented. It may also point to [4] above as a potential mechanism for calibrating switching thresholds to particular decision contexts. If the authors feel this is out of scope, it seems worth mentioning in the discussion.

The reviewer makes a couple of interesting suggestions here related to the idea that people may use a mixture of strategies that could evolve over time or across circumstances, rather than exclusively using compare-to-threshold computations. Of course, we cannot rule out the possibility that people use different strategies in different environments than those evaluated here, or in a random subset of trials. Nonetheless, we have amended the manuscript to show that participants' preference for the foraging-RL model over traditional RL is stable from the beginning to end of the sessions and between sessions (results shown below). Because the major result in this paper is that people use compare-to-threshold computations at all, we believe that developing and validating a hybrid model necessary to identify exactly how and when people switch between compare-to-threshold and compare-alternative strategies would make manuscript excessively diffuse and difficult to follow. We have therefore addressed this comment via amending the discussion section to more clearly explain that our results do not imply that no one ever uses compare-alternatives computations:

From the Discussion: Although the majority of participants' choices were better described as a compare-to-threshold process, we did find evidence of compare-alternatives computations in a small minority of participants (1.2%, 4.3%, 2.2% or 7.8%, depending on the analysis). We cannot rule out the possibility that this proportion would be higher in different tasks or in unexplored variants of this task (e.g., change point tasks, static bandits, bandit problems where reward magnitude drifts rather than probability, or bandit problems without feedback noise). Because the compare-to-threshold strategy is both computationally simpler and less rewarding than the compare-alternative strategy, it seems plausible that different results might be obtained if the stakes were higher and/or the cognitive demands lower. Indeed, when we raised the demands, increasing the number of options and he dimensionality of the task, the advantage of the foraging-RL model also grew—an intriguing parallel to the computational advantage that could come from adopting a strategy that need track only 1 value in increasingly high-dimensional environments. Of course, future within-subjects work is needed to determine if this result reflects a change in strategy across individuals or just some improved ability to discriminate the models in higher-dimensional environments. One manipulation that would be especially important in future studies is full outcome feedback (where the outcome of both chosen and unchosen options are explicitly provided, e.g., [16, 31]. Full feedback might encourage compare-alternatives strategies [16], though it would be important to differentiate true compare-alternatives computations (where option values are tracked independently over time) from a compare-to-threshold strategy that operates on differenced values (i.e., where relative value is calculated at the time of encoding, rather than at selection). Of course, we do not mean to exclude the possibility that humans use a repertoire of strategies, switching between

them as needed. For example, in the 3-alternative task, a substantive proportion of participants (16%) did not explore alternative options uniformly, though roughly equal proportions of these were biased towards the best alternative (consistent with using a compare-alternatives strategy for exploration) or towards the worst (consistent with an as-yet-unidentified exploratory strategy). While more work is clearly needed, these results do suggest that compare-to-threshold models should be evaluated even in tasks that are conventionally modeled using compare-alternatives computations.

As described above, we've also made additions to the manuscript that report stability in preference over time and across repetitions of the experiment. First, we examined the trial-by-trial choice likelihoods under each model for each participant over the course of each session in Experiment 1(Figure S3.A). We found no systematic bias towards one model or another at any point during the sessions. At the individual level, a minority of participants went from preferring one strategy to preferring another across blocks of 50 trials at the beginning and end (42 / 254; 16.5%; FigureS3.B), but these participants also had more ambiguous evidence for either strategy, making it unclear whether these were true strategy switches or just the result of the noise we might expect when fitting models to such a small a number of trials. Since we added a experiment where participants repeated the task twice, using a different response modality, we were also able to report in the revised manuscript that preference is relatively stable across repetitions of the task.

The time-varying analyses are described :
In the results: Choice likelihoods of both models were stable through time within participants (Figure S3A-B).

We have also added the following figure to the supplement:

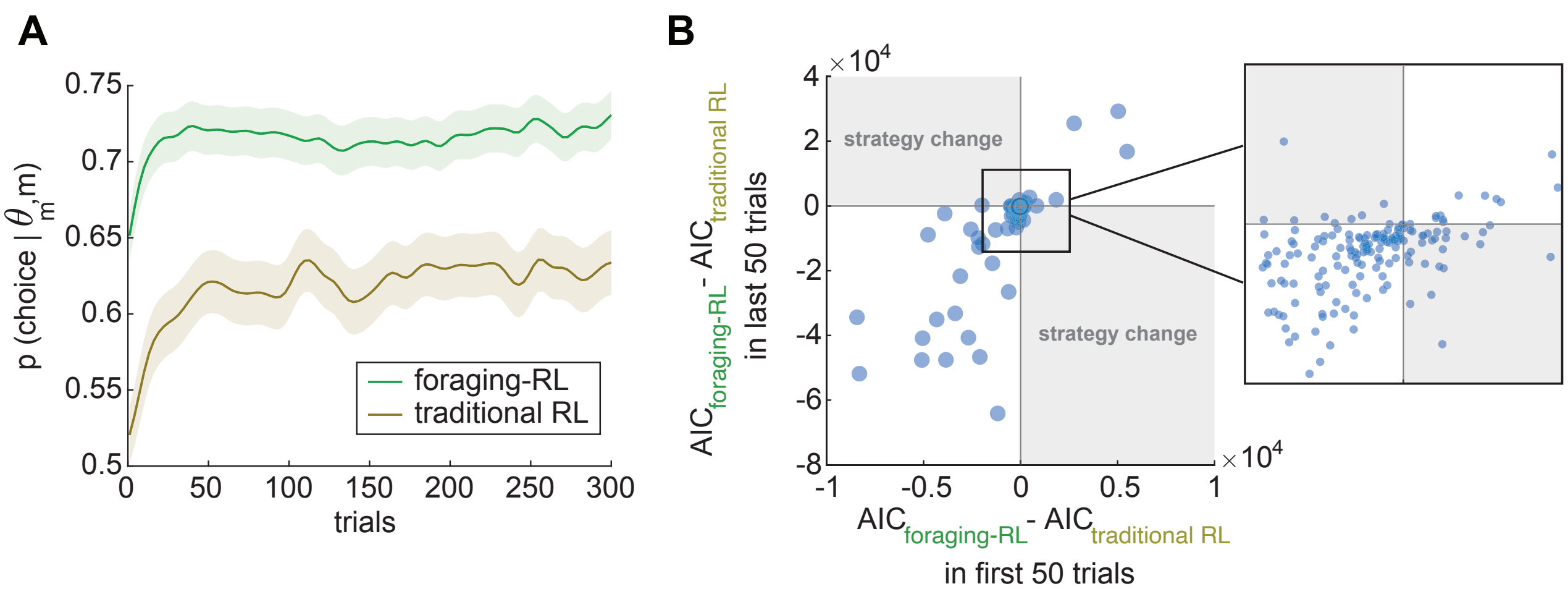

Figure S3: **Stability of model fits across the sessions.A)** Choice likelihood for each model as a function of trial number. Lines represent smoothed mean and shading represent $\pm SEM$ across participants in Experiment 1.**B)** To further explore whether people switched strategies, we compared model fit in the first 50 trials versus the last 50 trials of each session. For each participant, we calculated the AIC for both epochs and then computed the difference in AIC (foraging-RL - traditional RL). Each dot represents the AIC difference in the last 50 trials as a function in the first 50 trials. Participants who switched strategies would fall into either gray areas in the plot. We found that some participants did go from preferring one strategy to preferring another (42 / 254; 16.5%). However, the difference in relative evidence for the two models was an order of magnitude lower in these "strategy switching" participants (*50 first trials*; all participants mean ΔAIC [foraging - traditional] = -263.02 ; participants who switched strategies median ΔAIC [foraging - traditional] = -38.45; *50 last trials*; all participants median ΔAIC [foraging - traditional] = -2306 ; participants who switched strategies mean ΔAIC [foraging - traditional] = -121). This makes it difficult to determine how many of these participants truly switched strategies between the beginning and end of the task, versus how many had ambiguous evidence for the models in one or the other 50 trial bin and only appeared to switch due to noise in model fitting.

The repetition of the task with 2 response modality and the relevant analyses are described :

In the results : " We therefore fit the models to 4 additional datasets in which we manipulated (1) the independence of option values (Experiment 2; two options), (2) the volatility of values (Experiment 3, 3-options), (3) the number of options (Experiment 4; 2-, 3-, and 4-options), and (4) **the experimental setting (Experiment 5; in person, multiple response modalities)**. (...) Finally, in Experiment 5, we performed an in-person experiment with 29 participants (19 females, 10 males) in which we manipulated the modality of the response within-subjects. This manipulation was meant address the fact that participants in Experiments 1-4 were recruited via the online platform Amazon mTurk, which limits environmental control and could reduce participant engagement, and to determine if similar results would be obtained regardless of response modality. In the lab, each participant completed two versions of a 2-armed bandit task: one using a touchscreen and another using eye-tracking device (**Figure 5Q**). Order was randomized and counterbalanced. **Foraging-RL model parameters were reasonably correlated across testing blocks, though the threshold parameter ρ was the most stable (rho: $r = 0.79, p < 0.00001$; alpha: $r = 0.45, p < 0.002$; beta: $r = 0.35, p = 0.064$). These compared favorably with the parameter correlations observed with a traditional RL model (alpha r = 0.423; p = 0.0223; beta r = -0.062, p = 0.7477)**.

[6] The paper should acknowledge that the conclusions are drawn from a specific class of problems: k-arm bandit with drifting reward probabilities and binary observations. This makes the current observation, as well as past observations, less informative about the current latent option value than in bandit problems with fixed or infrequently abruptly changing values, and bandit problems with continuously valued observations. This may make the difference between RL and compare-to-threshold smaller than in other tasks. In addition, the data were collected on Amazon MTurk, where data quality is known to be often limited (although the average performance of the sample analyzed is reassuring). Acknowledging these two points seems useful.

We have amended the discussion to specify that our conclusions are specific to the restless, probabilistic bandit tasks studied here, and added an experiment were data were collected in the lab, in person. Results were similar to the MTurk experiments (Experiment 5).

In the first paragraph of the discussion: Here, in a restless, probabilistic k-armed bandit task, we found that human behavior is, on average, better explained by compare-to-threshold computations. (...) The foraging-RL model fits were consistently better than traditional RL model across multiple variations of the restless, probabilistic k-armed bandit task—including manipulations of volatility, response modality, number of options, uncertainty about the unchosen option, and experimental setting (i.e., both online and in person).

In the second paragraph of the discussion: (...) We cannot rule out the possibility that this proportion would be higher in a different task or in unexplored variants of this task (e.g., change point tasks, static bandits, bandit problems where reward magnitudes drift rather than probabilities, or bandit problems without feedback noise). It seems plausible that different results might be obtained in different tasks, especially if the stakes were higher or the cognitive demands lower.

We have added detail of the in-person version of the task to the Methods as follows:

In the Methods: **Experiment 5: In-person 2-option bandit task** We conducted an in-lab experiment using a 2-option restless bandit task to achieve two goals: (1) determine if compare-to-threshold computations are still preferred in an in-person setting (as opposed to online platforms like Amazon MTurk) and (2) test if different response modalities (eye tracking vs. touchscreen) affected choice of algorithm. The experiment consisted of 20-25 trials practice block to familiarize with the task followed by 2 testing blocks (1 for each modality: eye movements and touch screen presses). While online experiments used playing cards as stimuli, in-person experiments cued option locations with small equiluminant gabors and included small ($<$ 1degree) central fixations that had to be looked at (in eye modality) or touched (in touch modality) to initiate a trial and cause options to appear (after [13]) For eye modality responses, monocular eye data was sampled at 2,000 Hz with an Eyelink 1000+ infrared eye tracker. Saccades were identified in real-time using custom Matlab routines. Touch responses were recorded via an infrared touch frame (Chengying CYS-27) placed on top of the monitor and processed via the standard Paychtoolbox-3 routines. The modality assigned to the first block was randomized

to counterbalance order effects. Practice trials were excluded from all analyses. Data was collected between 9 AM and 5 PM EST. A total of 29 participants (19 females, 10 males) completed the task. Participants completed an average of 483.28 ± 43.01 trials in the eye-tracking version, and an average of 499.48 ± 1.70 in the touchscreen version.

And the results:

Foraging-RL better predicts behavior across different environments

Finally, in Experiment 5, we performed an in-person experiment with 29 participants (19 females, 10 males) in which we manipulated the modality of the response within-subjects. This manipulation was meant address the fact that participants in Experiments 1-4 were recruited via the online platform Amazon mTurk, which limits environmental control and could reduce participant engagement, and to determine if similar results would be obtained regardless of response modality. In the lab, each participant completed two versions of a 2-armed bandit task: one using a touchscreen and another using eye-tracking device (**Figure 5Q**). Order was randomized and counterbalanced. Foraging-RL model parameters were reasonably correlated across testing blocks, though the threshold parameter ρ was the most stable (rho: $r = 0.79, p < 0.00001$; alpha: $r = 0.45, p < 0.002$; beta: $r = 0.35, p = 0.064$). These compared favorably with the parameter correlations observed with a traditional RL model (alpha r = 0.423; p = 0.0223; beta r = -0.062, p = 0.7477). The foraging-RL model again provided a better fit to human behavior than the traditional RL model at the group level, independently of the response modality (touchscreen or eye-tracking) and condition order (**Figure 5R**, relative *traditional RL* AIC weights for all presentation orders and response modalities $< 10^{-15}$, see Supplemental Table 6). While model comparison results were not significant at the individual level in this small sample, we again found that, the foraging-RL better accounted for individual participants' tendency to switch between options in both the eye-tracking task, and the touchscreen task (**Figure 5S-T**; eye-tracking: *foraging-RL model*: RMSE = 0.045 (95% CI = [0.036, 0.054]); *traditional RL*: RMSE = 0.11, (95% CI = [0.095,0.12]); significant Wilcoxon signed rank test, $W = 377, p < 0.0006$); touchscreen: *foraging-RL*: RMSE = 0.061 (95% CI = [0.053, 0.069]); *traditional RL*: RMSE = 0.099, (95% CI = [0.091,0.11]); significant Wilcoxon signed rank test, $W = 398, p < 0.0001$)). Further, we also found that the foraging-index, a measure of the relative goodness of model fit (see Methods), was correlated across modalities ($r = 0.63, p < 0.0005$). Overall, these findings show that participants consistently used the compare-to-threshold strategy, regardless of the responses modality (touchscreen vs. eye-tracking) and the environmental setting (online vs. in-person).

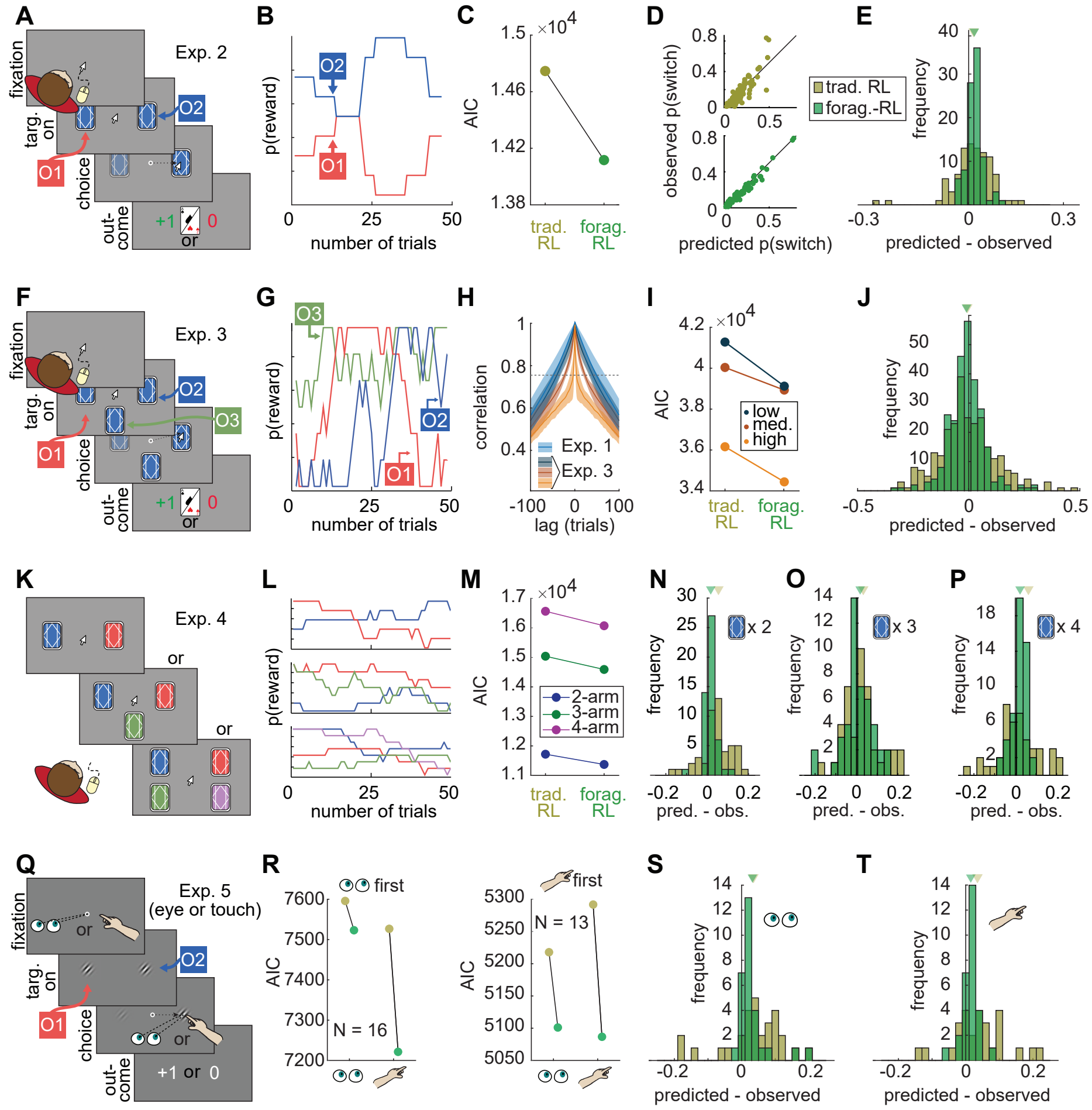

Figure 5: **Foraging-RL better describes participants' behavior across multiple tasks**. **A)** Schematic representation of Experiment 2. Participants (n = 95) performed a 2-option bandit task in which option values always summed to 1. **B)** Example reward schedule (50-trial portion) from Experiment 2 (red trace = option 1; blue trace = option 2). **C)** The AIC of traditional RL and foraging-RL model fits to all data from Experiment 2. **D)** Participants switch probabilities as a function of predicted probability of switch from simulations of the traditional RL model (*top*), and the foraging-RL model (*bottom*). Dots = participants. **E)** Distribution of the difference in predicted versus observed switch probability for both models. Colors as in D, carets = means. **F)** Experiment 3. Participants (n = 270) performed a volatile 3-option bandit task where changes in option values occurred more rapidly than in Experiments 1 and 2. **G)** An example reward schedule (50-trial portion) experienced by one participant from Experiment 3. **H)** Auto-correlation in reward schedules averaged for all participants of Experiment 1 (*blue*), and the 3 volatility bins in Experiment 3 (*dark purple, red, orange*). Note that even the "low" volatility environment is higher volatility than Experiment 1. Full line indicates the mean, shading indicates STD and dotted line indicates the decay threshold. **I)** The AIC of traditional RL and foraging-RL model fits to all data from Experiment 3. **J)** Same as *E* for Experiment 3. **K)** Experiment 4. Participants (n = 150) performed 2-, 3-, or 4-option bandit tasks (50 participants per condition). **L)** Example reward schedules (50-trial portion) from Experiment 4 for 2- (*top*), 3- (*mid*) and 4- (*bottom*) option conditions. **M)** The AIC of traditional RL and foraging-RL model fits to all data from Experiment 4. **N-P)** Same as *E* for 2- (*N*), 3- (*O*) and 4- (*P*) option conditions. **Q)** Experiment 5. Participants (n = 29) performed two versions of a 2-armed bandit task in person and in randomized order. In the "touch" version, participants physically touched the chosen option on a touchscreen. In the "eye" version, participants saccaded to the desired option and held fixation to select it. **R)** (*left*) The AIC of traditional RL and foraging-RL model fits to data from Experiment 5 for either participants who experienced the eye (*left*) or touch version of the task first (*right*). **S-T)** Distribution of the difference in predicted versus observed switch probability for both models for the eye-tracking modality (*S*) and the touchscreen modality (*T*). caret = mean.

presentation order	response modality	model	total parameters (k)	AIC	log likelihood
**eye-tracking first**	**eye-tracking (1st)**	foraging-RL	3×16	7,523.5	-3,758.7
		traditional RL	2×16	7,596.3	-3,796.1
	**touchscreen (2nd)**	foraging-RL	3×16	7,221.4	-3,607.7
		traditional RL	2×16	7,470.0	-3,733.0
**touchscreen first**	**eye-tracking (2nd)**	foraging-RL	3×13	5,101.1	-2,547.6
		traditional RL	2×13	5,218.1	-2,607.0
	**touchscreen (1st)**	foraging-RL	3×13	5,086.7	-2,540.4
		traditional RL	2×13	5,256.7	-2,626.3

Table 6: **Group-level model comparison for Experiment 5.**

Minor:

- it seems that the win-stay-loose-shift (in a probabilistic version that chooses randomly with a probability epsilon) would be a useful baseline to include in the paper. It is mentioned briefly at the beginning of the paper as a very good, simple model of the data; it could be included more exhaustively in all figures and analysis, being a simpler model than RL and forage.

We fit a one-parameter win-stay–lose-shift (WSLS) model to participants' behavior in all Experiments. The quality of model fit was so poor that it was difficult to include it exhaustively throughout the manuscript without obscuring the main effects. However we have updated the text in the main manuscript to include WSLS as a baseline for comparison and added multiple additional comparisons as a supplemental figure related to Experiment 1.

In the results: We found that the standard Foraging-RL model was a better fit to participant behavior than the standard RL model (Figure 3G-H; standard foraging-RL: AIC = 47,675, 3 parameters by 254 participants = 762 parameters; log-likelihood = -23,075, standard traditional RL: AIC = 49,840, relative AIC weight $< 1.8 * 10^{-32}$, 2 parameters per participant, 508 total, log-likelihood = -24,413; 76,200 total trials), **with both models substantially outperforming a 1-parameter Win-Stay-Lose-Shift model (WSLS: AIC= 84,740, log-likelihood = -42,116)**. (...)

We simulated data from traditional RL and foraging-RL agents that were matched to the participants' environment and parameters (see Methods; and the 1-parameter WSLS: Supplementary Figure S4).

New Supplemental Figure S4 illustrates behavior that was simulated from fitted WSLS algorithms, showing that this model does an even poorer job at explaining participant switching than either the foraging-RL or traditional RL models.

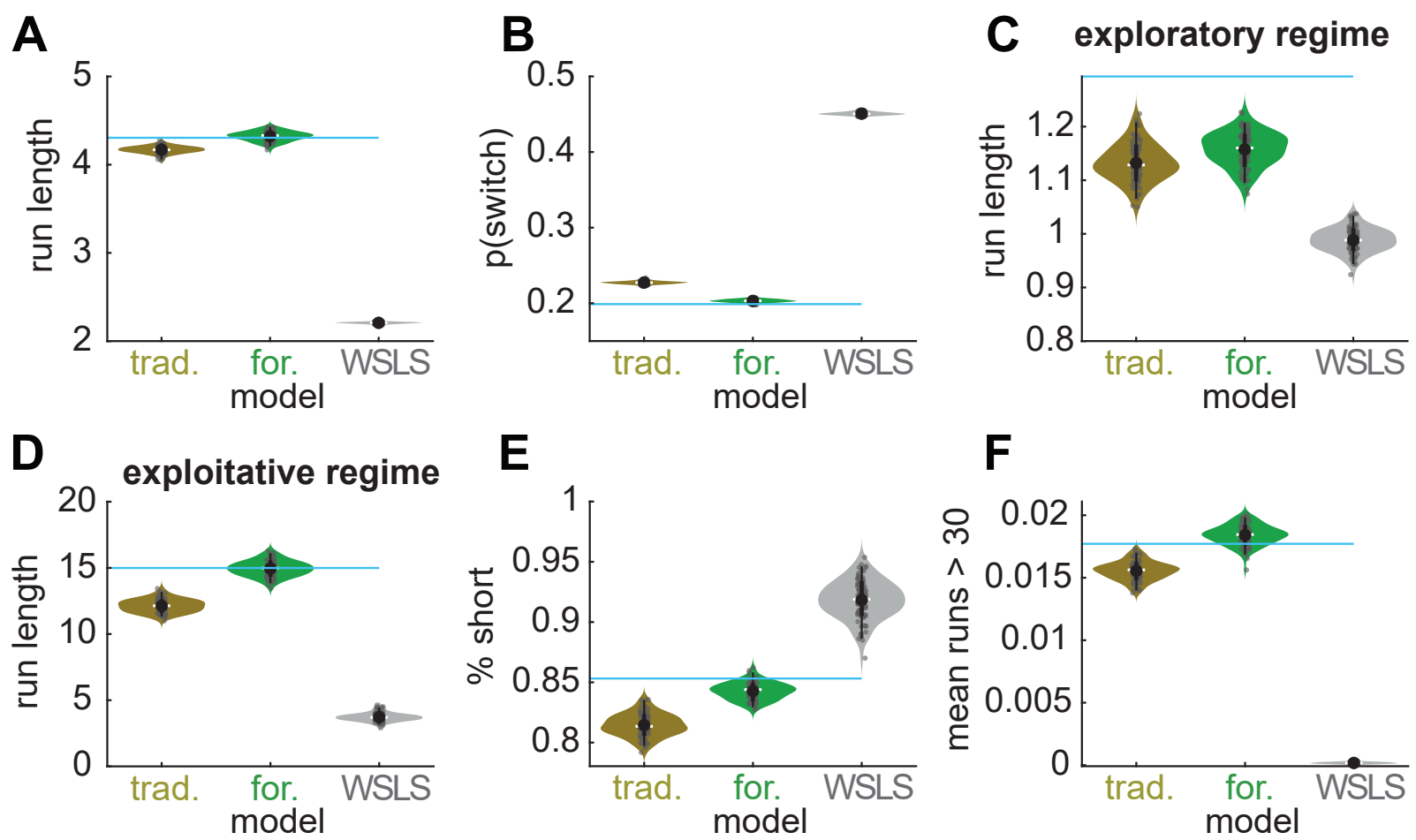

Figure S4: **Traditional RL and foraging-RL models predict behaviour more accurately than a simple win-stay lose-shift model A)** Average number of trials between switch decisions (i.e., run length) of traditional RL (*gold*) and foraging-RL (*green*) and win-stay loose-shift (*gray*) agents in simulated Experiment 1 (blue line = average for Experiment 1 participants). **B)** Probability of switch. Same conventions as in *A*. **C)** Average run length in the exploratory regime. **D)** Average run length in the exploitative regime. **E)** Proportion of exploratory regimes. **F)** Proportion of very long exploitative regimes exceeding 30 consecutive trials.

In the methods:

Win-Stay Lose-Shift Model A very simple, value-free strategy that can solve a restless multi-armed bandit is the Win-Stay Lose-Shift (WSLS) algorithm [33]. As the name suggests, the algorithm simply repeats a choice if a reward was delivered, and switches away if the reward was omitted. Because this model was only fit to 2-alternative data here, that meant switching to the only alternative option. For flexibility, in this work we implemented an ϵ-greedy version of WSLS where there is a probability ϵ to make the opposite choice (i.e., to stay following a loss or switch following a win), and a probability $1-\epsilon$ to follow the outcome-contingent WSLS algorithm described before. In short,

$$p(\text{stay}) = \begin{cases} 1-\epsilon & \text{if} \quad r = 1 \\ \epsilon & \text{if} \quad r = 0, \end{cases}$$

where $\epsilon \in [0,1]$. It follows from this that $p(\text{switch}) = 1 - p(\text{stay})$, $p(\text{choice} = i) = p(\text{stay})$ if and only if choice i was made on the last trial, and $p(\text{choice} = i) = \frac{p(\text{switch})}{n-1}$ otherwise, where n is the number of options.

- please show the model predictive checks for Fig 2D (i.e. corresponding values from the fitted models)

Figure 2D did not include fitted models on which we could perform model predictive checks. We have addressed this comment via clarifying the analyses we performed in the “fingerprint” section, in the Results, Methods, and Figure 2 of the revised manuscript. Briefly, we have clarified that these plots were straightforward analyses of the reward walks themselves, not simulations from models.

Methods:

Strategy Fingerprints

Compare-alternatives and compare-to-threshold decision-making should, in theory, depend on different aspects of the environment. To quantify these predicted dependencies we characterized the fingerprints of each strategy as a function of two features of the environment: richness (the sum of the values) and

discriminability (the range of the values, normalized to the sum). In order to make predictions for each strategy, we first separated the 258 reward walks experienced by the participants into 100-trial bins (3 bins per session, 774 bins). We then calculated the richness and discriminability within each bin (Figure 2C, *bottom*) and the "environmental predicted p(switch)" for each bin, given each of the two strategies (Figure 2D-E). Because exploration should occur most often when option values are close together in the compare-alternatives strategy, the environment-predicted p(switch) under this strategy was calculated as the probability that the difference in option values was below some Δ reward for various $\Delta rewards$ (4 examples $\Delta rewards$ in Figure 2D). Because exploration should only occur when the focal option is below threshold in the compare-to-threshold strategy, the environment-predicted p(switch) under this strategy was the probability that any randomly selected "focal" option value would be below some threshold for various thresholds (4 examples thresholds in Figure 2E).

To quantify how similar participants' switching patterns were to the compare-alternatives and compare-to-threshold fingerprints, we estimated the shape of the environmental predicted p(switch) at each parameter value for each strategy (i.e., threshold or $\Delta reward$). We did this via fitting generalized linear models (GLMs) to the environment-predicted p(switch) from both richness and discriminability. Two models were fit, a linear (2 parameter) GLM and a quadratic (3 parameter) GLM, for each combination of parameter, strategy, and independent variable. We then extracted 3 key features from each pair of fitted models: (1) the relative deviance of the linear and quadratic models (a measure of the extent of curvature as a function of either richness or discriminability), (2) the quadratic term of quadratic model (a measure of the sign and magnitude of curvature in the relationship), and (3) the linear term of the linear model (a measure of the overall slope of the relationship). This approach allowed us to precisely quantify how switch probabilities were predicted to change as a function of both richness and discriminability under each hypothesized strategy. Distributions of the features for every possible threshold and $\Delta reward$ are illustrated in S1.

To consider the fingerprints holistically, rather than as individual features, we then took the 6-feature dimensions described above, added the overall probability of switching to account for DC offsets in switch probabilities as a 7th feature, and then calculated the distance from the human fingerprint to the predicted fingerprint for every possible parameter value for each of the two strategies (Figure 2H). PCA was performed only to illustrate the approach in Figure 2G, where distances were calculated in the full space. In this plot, bounding boxes are drawn around all environment-predicted fingerprints under each strategy to illustrate the range of fingerprints that each strategy was capable of generating. Similar results were found regardless of whether GLMs were fit with binomial or identity link functions. The former appears in Figure 2G, the latter is shown in Figure S1A-C).

Results : The compare-alternatives and compare-to-threshold views also differ in their predictions about how people should decide to explore as a function of the environment (Figure 2A-B). Specifically, the compare-alternatives and compare-to-threshold views predict that switching will be most frequent in different situations because there are different mechanisms for exploration in the two cases. The compare-alternatives view predicts that people are the most likely to explore when it is difficult to discern which option is the best. Switching would therefore occur most frequently when the option values are close together (i.e., when the $\Delta reward$ between the options is small; Figure 2A). This happens most often in **ambiguous** environments, rather than *discriminable* environments. Conversely, compare-to-threshold view predicts that people explore whenever an exploited option falls below a threshold (Figure 2B). Switching would therefore occur most frequently when the value of any option is more likely to fall below a threshold. This happens most often when all option values are low, in **poor** environments rather than *rich* environments, when no option can fall below threshold. Note that this is true even without assuming that the compare-to-threshold algorithm calculates richness: compare-to-threshold choices will inevitably depend on richness as a byproduct of the fact that options are more likely to be above threshold in rich environments. In short, switching should depend on different statistics of the environment in compare-to-threshold decision-making versus compare-alternatives decision-making.

These insights imply that we should be able to extract distinct "fingerprints" of these two strategies as a function of the environment. Regardless of implementation, switching probability in compare-to-threshold decision-making should depend on richness (the sum of values), while it should depend on discriminability (the differences between values) in compare-alternatives decision-making. However, in any bounded environment the sum of values and difference between values will be related (Figure 2C; i.e., any time outcomes are probabilistic or there are floor and/or ceilings on outcome magnitude). When

richness is high (value of all options are close to their maximum) or low (value of all options close to their minimum), discriminability can only be low. However, when richness is at intermediate levels, discriminability can range from low (e.g., all values around their midpoint) to high (e.g., a mix of probabilities close to min and max). As a consequence of these bounds, compare-alternatives decision-making should have a U-shaped relationship with richness, with minimal switching at intermediate levels where discriminability is highest (Figure 2D, *bottom*). By contrast, compare-to-threshold decision-making will have an inverted U-shaped relationship with discriminability, driven by the statistical relationship between richness and discriminability (Figure 2E, *top*) and both strategies have a unique fingerprint as a function of both variables (Figure 2D-E).

We found that the participants switched options the most at intermediate, rather than low levels of discriminability (Figure 2F, *top*), consistent with compare-to-threshold computations (Figure 2E,*top*). The participants were also less likely to switch in rich environments, compared to poor ones (Figure 2F, *bottom*), again consistent with compare-to-threshold computations (Figure 2E, *bottom*). To quantify the relationship between participants' data and the compare-alternatives and compare-to-threshold fingerprints, we calculated the probability of each strategy switching frequently as a function of discriminability and richness (i.e., environment predicted p(switch); Figure 2D-E; see Methods). We then measured the slope and curvature of the relationship to discriminability and richness under each hypothesis (Figure 2G-H; see Methods). Human switching was sensitive to discriminability and richness in a way that better resembled compare-to-threshold decision making, both in terms of the individual features (Figure S1A-C) and in a multivariate feature space that considered all features simultaneously (Figure 2G-H and Figure S1D-E; median distance to $\Delta reward$ in the full feature space = 39.41, range = [22.04, 437.07]; to threshold = 6.80, range = [3.76,9.78] ; significant Wilcoxon rank sum test, $w = 92, p < 0.001$; see Methods). While the human data was not a perfect match for either fingerprint, it better resembled the fingerprint of compare-to-threshold decision-making than the fingerprint of compare-alternatives decision-making.

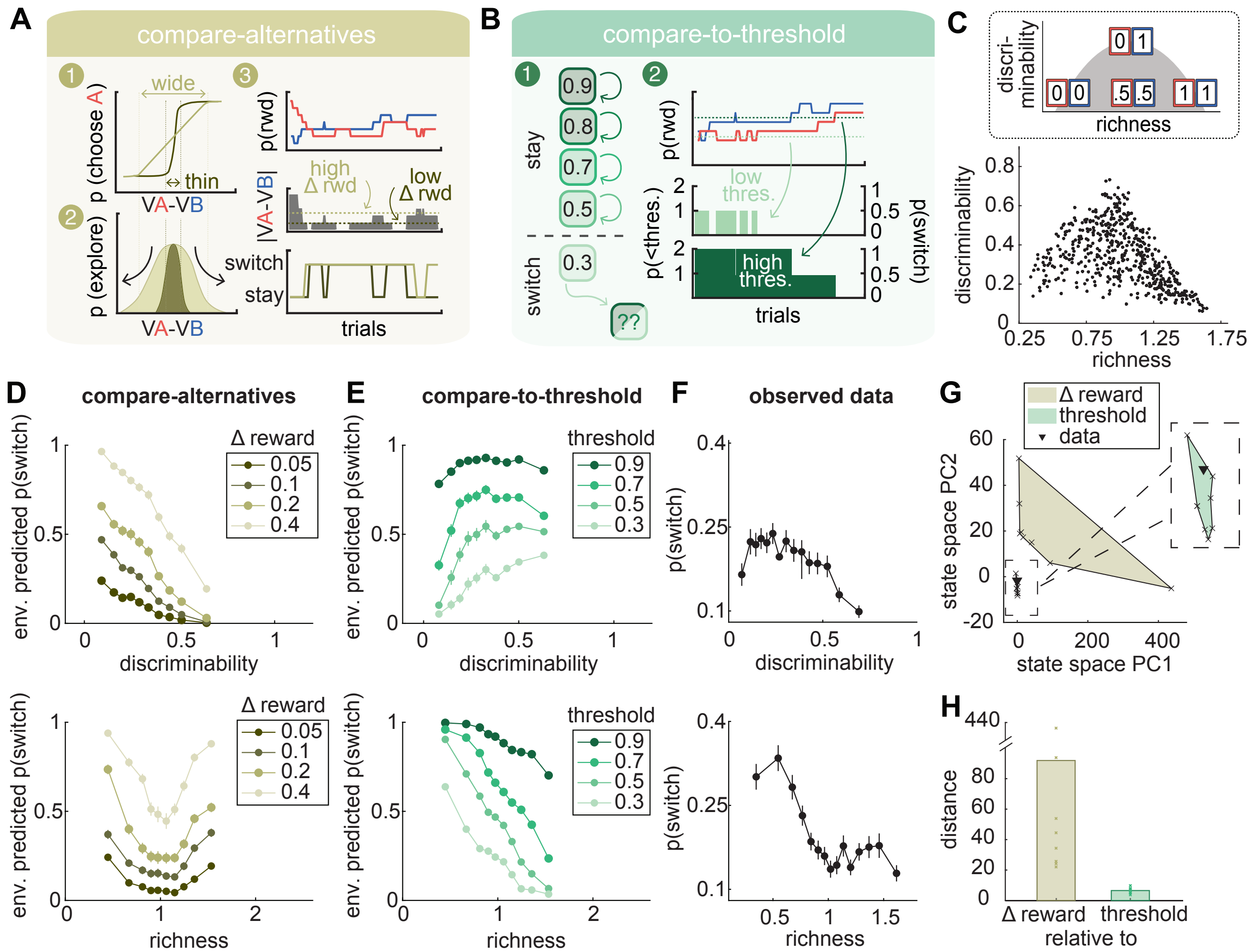

Figure 2: **Dissociable signatures of compare-to-threshold and compare-alternatives decisions to explore as a function of environmental statistics. A)** In compare-alternatives decision-making, exploration is driven by noise in value comparison. This is typically modeled via a softmax decision rule in which noise is larger when values are close together. Noise scales with inverse temperature, such that exploration occurs across a wider range of value differences when inverse temperature is low, compared to when it is high. In either case, switching occurs the least often at the tails, when differences in value are highest. As a result, we can predict how often participants should switch from the discriminability of the environment in this cartoon. **B)** In compare-to-threshold decision-making, the decision to stay depends only on the value of the focal option. If the value drops below some threshold, the algorithm switches. This means that switching essentially does not occur when all options are above threshold and occurs in proportion to the number of options that are below threshold otherwise. As a result, we can predict how often participants should switch from the richness of the environment in this cartoon. **C)** *top*: Cartoon illustrating the relationship between richness and discriminability for 4 example pairs of probabilistic rewards. *bottom*: Discriminability as a function of richness in the reward walks in Experiment 1. Each dot represent discriminability and richness in a 100-trial bin (see Methods). **D)** Probability of the difference in option values being below some $\Delta reward$ ("environment predicted p(switch)" for compare-alternatives) as a function of discriminability (*top*) and richness (*bottom*) for 4 values of $\Delta reward$ (*shades of gold*). **E)** Probability of any random option value below some threshold ("environment predicted p(switch)" for compare-to-threshold) as a function of discriminability (*top*) and richness (*bottom*) for 4 threshold values (*shades of green*). **F)** Probability of switching for all participants in Experiment 1 as a function of different levels of reward discriminability *(top)* and richness *(bottom)* of the environment (errorbar = SEM). **G)** First 2 PCs of the multivariate feature space of compare-alternatives fingerprints (*gold*) and compare-to-threshold fingerprints (*green*) (see Methods). Bounds encompass all possible values for the $\Delta reward$ and threshold parameters. x = each parameter value, black caret = humans. **H)** Distance between humans and every (*gold*) and (*green*) in the full 7-dimensional space. x = individual parameter values.

- the delta rule model for V-exploit seems not completely consistent. In particular, the model reinforces the exploit decision when the agent is rewarded for switching. I understand why it is the case, but it may be more intuitive to present the model as increasing the probability to repeat whenever the current choice is rewarded, with an intercept that corresponds to the (negative) threshold rho.

This is an interesting suggestion. As we understand it, it is clear that the architecture of the model is such that getting a reward increases the probability of exploiting the option that was just chosen, and not getting a reward decreases that probability. And it is clear that this is true regardless of how many times that option has been chosen, although any change in the exploited action resets Vexploit to the threshold. In this case, our understanding of the argument is that it would be more intuitive to say instead that reward increases the probability of repeating the option that was just chosen, rather than the probability of exploiting it. Assuming that our understanding is correct, yes, it makes sense that something doesn't feel entirely intuitive about the idea of "exploiting" something that you just chose for the first time, especially as we tend to think of exploitation as a sustained behavior. After some debate, we ultimately decided to continue using the term "exploit" to refer to the alternative to the "explore" option. We believe this term better matches how we think of these processes–as temporally-extended options, rather than one off decisions–and also how we interpret Vexploit, as the value of continuing (versus terminating) the exploitation option. We have also gone through the Methods section where we describe the foraging-RL model and added some clarifying language, just in case we misunderstood the issue at hand here.

Methods: In the foraging-RL models, values do not represent the value of a single focal option. Instead, the only value tracked in the model is the value of staying with or "exploiting" the option that was just chosen.

And

It is important to note that the foraging-RL algorithm only tracks one value, the value of the exploited option. Whenever the model switches to a new primitive action (explores), the value of exploiting that action is unknown, so the value of exploitation is reset to the threshold. In this way, the value of exploitation always represents the value of terminating a sequences of choices to a particular option, even if the model has only just begun choosing that primitive action.

- l. 28: rath(er: aberrant bracket
- l.107: Fig 2A shows that when discriminability is low, richness can only be very low or very high. Fig 2A shows that it can be any level of richness. I think what the authors want to say is the other way around: when richness is very high/low, discriminability can only be low.
- l. 233: moreso: more so
- l. 391: option to to try: delete to
- l. 395: an more: a more

Thanks for catching our typos, these have been corrected in the revised manuscript. Furthermore, to clarify our explanation of this figure panel, we (1) slightly modified the hypothesis figure it itself (now refereed to as **Figure 2c - top**), and (2) modified the text in the manuscript to better explain the relationship between discriminability and richness.

see revised text below:

When richness is high (value of all options are close to their maximum) or low (value of all options close to their minimum), discriminability can only be low. However, when richness is at intermediate levels, discriminability can range from low (e.g., all values around their midpoint) to high (e.g., a mix of probabilities close to min and max).

see updated figure :

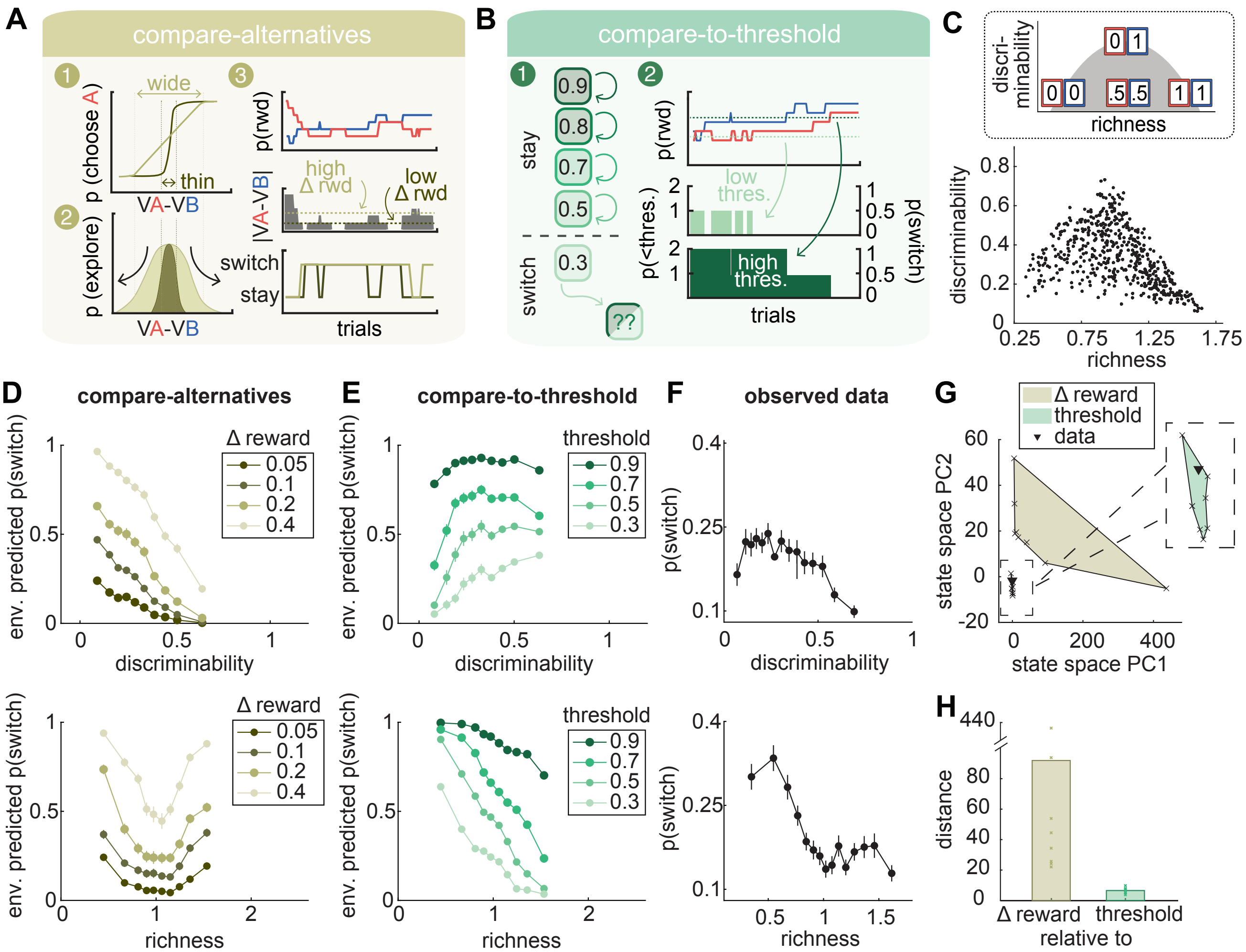

Figure 2: **Dissociable signatures of compare-to-threshold and compare-alternatives decisions to explore as a function of environmental statistics. A)** In compare-alternatives decision-making, exploration is driven by noise in value comparison. This is typically modeled via a softmax decision rule in which noise is larger when values are close together. Noise scales with inverse temperature, such that exploration occurs across a wider range of value differences when inverse temperature is low, compared to when it is high. In either case, switching occurs the least often at the tails, when differences in value are highest. As a result, we can predict how often participants should switch from the discriminability of the environment in this cartoon. **B)** In compare-to-threshold decision-making, the decision to stay depends only on the value of the focal option. If the value drops below some threshold, the algorithm switches. This means that switching essentially does not occur when all options are above threshold and occurs in proportion to the number of options that are below threshold otherwise. As a result, we can predict how often participants should switch from the richness of the environment in this cartoon. **C)** *top*: Cartoon illustrating the relationship between richness and discriminability for 4 example pairs of probabilistic rewards. *bottom*: Discriminability as a function of richness in the reward walks in Experiment 1. Each dot represent discriminability and richness in a 100-trial bin (see Methods). **D)** Probability of the difference in option values being below some $\Delta reward$ ("environment predicted p(switch)" for compare-alternatives) as a function of discriminability (*top*) and richness (*bottom*) for 4 values of $\Delta reward$ (*shades of gold*). **E)** Probability of any random option value below some threshold ("environment predicted p(switch)" for compare-to-threshold) as a function of discriminability (*top*) and richness (*bottom*) for 4 threshold values (*shades of green*). **F)** Probability of switching for all participants in Experiment 1 as a function of different levels of reward discriminability *(top)* and richness *(bottom)* of the environment (errorbar = SEM). **G)** First 2 PCs of the multivariate feature space of compare-alternatives fingerprints (*gold*) and compare-to-threshold fingerprints (*green*) (see Methods). Bounds encompass all possible values for the $\Delta reward$ and threshold parameters. x = each parameter value, black caret = humans. **H)** Distance between humans and every (*gold*) and (*green*) in the full 7-dimensional space. x = individual parameter values.

References

[1] Merideth A. Addicott, John M. Pearson, Jessica Wilson, Michael L. Platt, and F. Joseph McClernon. Smoking and the bandit: A preliminary study of smoker and non-smoker differences in exploratory behavior measured with a multi-armed bandit task. *Experimental and clinical psychopharmacology*, 21(1):66–73, February 2013. ISSN 1064-1297. doi: 10.1037/a0030843. URL https://pmc.ncbi.nlm.nih.gov/articles/PMC4028629/.

[2] Nathaniel J. Blanco, A. Ross Otto, W. Todd Maddox, Christopher G. Beevers, and Bradley C. Love. The influence of depression symptoms on exploratory decision-making. *Cognition*, 129 (3):563–568, December 2013. ISSN 0010-0277. doi: 10.1016/j.cognition.2013.08.018. URL https://www.sciencedirect.com/science/article/pii/S0010027713001753.

[3] Evan C. Carter and A. David Redish. Rats value time differently on equivalent foraging and delay-discounting tasks. *Journal of Experimental Psychology. General*, 145(9):1093–1101, September 2016. ISSN 1939-2222. doi: 10.1037/xge0000196.

[4] Eric L. Charnov. Optimal foraging, the marginal value theorem. *Theoretical Population Biology*, 9(2):129–136, April 1976. ISSN 0040-5809. doi: 10.1016/0040-5809(76)90040-X. URL https://www.sciencedirect.com/science/article/pii/004058097690040X.

[5] Cathy S Chen, Evan Knep, Autumn Han, R Becket Ebitz, and Nicola M Grissom. Sex differences in learning from exploration. *eLife*, 10:e69748, November 2021. ISSN 2050-084X. doi: 10.7554/eLife.69748. URL https://doi.org/10.7554/eLife.69748. Publisher: eLife Sciences Publications, Ltd.

[6] Cathy S. Chen, Evan Knep, Veldon-James Laurie, Olivia Calvin, R. Becket Ebitz, Melissa Fisher, Michael-Paul Schallmo, Scott R. Sponheim, Matthew V. Chafee, Sarah R. Heilbronner, Nicola M. Grissom, A. David Redish, Angus W. MacDonald, Sophia Vinogradov, and Caroline Demro. Beyond reward learning deficits: Exploration-exploitation instability reveals computational heterogeneity in value-based decision making in early psychosis, May 2025. URL https://www.medrxiv.org/content/10.1101/2025.04.29.25326698v1. Pages: 2025.04.29.25326698.

[7] Sara M. Constantino and Nathaniel D. Daw. Learning the opportunity cost of time in a patch-foraging task. *Cognitive, Affective, & Behavioral Neuroscience*, 15(4):837–853, December 2015. ISSN 1531-135X. doi: 10.3758/s13415-015-0350-y. URL https://doi.org/10.3758/s13415-015-0350-y.

[8] Vincent D. Costa, Andrew R. Mitz, and Bruno B. Averbeck. Subcortical Substrates of Explore-Exploit Decisions in Primates. *Neuron*, 103(3):533–545.e5, August 2019. ISSN 0896-6273. doi: 10.1016/j.neuron.2019.05.017. URL https://www.sciencedirect.com/science/article/pii/S0896627319304428.

[9] Nathaniel D. Daw, John P. O'Doherty, Peter Dayan, Ben Seymour, and Raymond J. Dolan. Cortical substrates for exploratory decisions in humans. *Nature*, 441(7095):876–879, June 2006. ISSN 1476-4687. doi: 10.1038/nature04766. URL https://www.nature.com/articles/nature04766. Publisher: Nature Publishing Group.

[10] Nathaniel D. Daw, Samuel J. Gershman, Ben Seymour, Peter Dayan, and Raymond J. Dolan. Model-Based Influences on Humans' Choices and Striatal Prediction Errors. *Neuron*, 69(6):1204–1215, March 2011. ISSN 0896-6273. doi: 10.1016/j.neuron.2011.02.027. URL https://www.cell.com/neuron/abstract/S0896-6273(11)00125-5. Publisher: Elsevier.

[11] Lorenz Deserno, Rebecca Boehme, Christoph Mathys, Teresa Katthagen, Jakob Kaminski, Klaas Enno Stephan, Andreas Heinz, and Florian Schlagenhauf. Volatility Estimates Increase Choice Switching and Relate to Prefrontal Activity in Schizophrenia. *Biological Psychiatry: Cognitive Neuroscience and Neuroimaging*, 5(2):173–183, February 2020. ISSN 2451-9022. doi: 10.1016/j.bpsc.2019.10.007. URL https://www.sciencedirect.com/science/article/pii/S245190221930271X.

[12] Magda Dubois and Tobias U. Hauser. Value-free random exploration is linked to impulsivity. *Nature Communications*, 13(1):4542, August 2022. ISSN 2041-1723. doi: 10.1038/s41467-022-31918-9. URL https://www.nature.com/articles/s41467-022-31918-9. Publisher: Nature Publishing Group.

[13] R. Becket Ebitz, Eddy Albarran, and Tirin Moore. Exploration Disrupts Choice-Predictive Signals and Alters Dynamics in Prefrontal Cortex. *Neuron*, 97(2):450–461.e9, January 2018. ISSN 1097-4199. doi: 10.1016/j.neuron.2017.12.007.

[14] R. Becket Ebitz, Brianna J. Sleezer, Hank P. Jedema, Charles W. Bradberry, and Benjamin Y. Hayden. Tonic exploration governs both flexibility and lapses. *PLOS Computational Biology*, 15(11):e1007475, November 2019. ISSN 1553-7358. doi: 10.1371/journal.pcbi.1007475. URL https://journals.plos.org/ploscompbiol/article?id=10.1371/journal.pcbi.1007475. Publisher: Public Library of Science.

[15] Charles Findling and Valentin Wyart. Computation noise in human learning and decision-making: origin, impact, function. *Current Opinion in Behavioral Sciences*, 38: 124–132, April 2021. ISSN 2352-1546. doi: 10.1016/j.cobeha.2021.02.018. URL https://www.sciencedirect.com/science/article/pii/S2352154621000401.

[16] Charles Findling, Vasilisa Skvortsova, Rémi Dromnelle, Stefano Palminteri, and Valentin Wyart. Computational noise in reward-guided learning drives behavioral variability in volatile environments. *Nature Neuroscience*, 22(12):2066–2077, December 2019. ISSN 1546-1726. doi: 10.1038/s41593-019-0518-9. URL https://www.nature.com/articles/s41593-019-0518-9. Publisher: Nature Publishing Group.

[17] Michael J. Frank, Bradley B. Doll, Jen Oas-Terpstra, and Francisco Moreno. Prefrontal and striatal dopaminergic genes predict individual differences in exploration and exploitation. *Nature Neuroscience*, 12(8):1062–1068, August 2009. ISSN 1546-1726. doi: 10.1038/nn.2342. URL https://www.nature.com/articles/nn.2342. Publisher: Nature Publishing Group.

[18] Dalin Guo and Angela Yu. Human learning and decision-making in the bandit task: Three wrongs make a right. In *2019 Conference on Cognitive Computational Neuroscience*, Berlin, Germany, 2019. Cognitive Computational Neuroscience. doi: 10.32470/CCN.2019.1356-0. URL https://ccneuro.org/2019/Papers/ViewPapers.asp?PaperNum=1356.

[19] Dalin Guo and Angela J Yu. Why so gloomy? A Bayesian explanation of human pessimism bias in the multi-armed bandit task. In *Advances in Neural Information Processing Systems*, volume 31. Curran Associates, Inc., 2018. URL https://proceedings.neurips.cc/paper$_files/paper/2018/hash/f55cadb97eaff2ba1980e001b0bd9842-Abstract.html$.

[20] Nora C. Harhen and Aaron M. Bornstein. Overharvesting in human patch foraging reflects rational structure learning and adaptive planning. *Proceedings of the National Academy of Sciences of the United States of America*, 120(13):e2216524120, March 2023. ISSN 1091-6490. doi: 10.1073/pnas.2216524120.

[21] Benjamin Y Hayden, John M Pearson, and Michael L Platt. Neuronal basis of sequential foraging decisions in a patchy environment. *Nature neuroscience*, 14(7):933–939, June 2011. ISSN 1097-6256. doi: 10.1038/nn.2856. URL https://pmc.ncbi.nlm.nih.gov/articles/PMC3553855/.

[22] John M. C. Hutchinson, Andreas Wilke, and Peter M. Todd. Patch leaving in humans: can a generalist adapt its rules to dispersal of items across patches? *Animal Behaviour*, 75(4):1331–1349, April 2008. ISSN 0003-3472. doi: 10.1016/j.anbehav.2007.09.006. URL https://www.sciencedirect.com/science/article/pii/S0003347207005003.

[23] Christian Ineichen, Hannes Sigrist, Simona Spinelli, Klaus-Peter Lesch, Eva Sautter, Erich Seifritz, and Christopher R. Pryce. Establishing a probabilistic reversal learning test in mice: evidence for the processes mediating reward-stay and punishment-shift behaviour and for their modulation by serotonin. *Neuropharmacology*, 63 (6):1012–1021, November 2012. ISSN 1873-7064. doi: 10.1016/j.neuropharm.2012.07.025.

[24] Marieke Jepma and Sander Nieuwenhuis. Pupil diameter predicts changes in the exploration-exploitation trade-off: evidence for the adaptive gain theory. *Journal of Cognitive Neuroscience*, 23(7):1587–1596, July 2011. ISSN 1530-8898. doi: 10.1162/jocn.2010.21548.

[25] Gary A Kane, Aaron M Bornstein, Amitai Shenhav, Robert C Wilson, Nathaniel D Daw, and Jonathan D Cohen. Rats exhibit similar biases in foraging and intertemporal choice tasks. *eLife*, 8:e48429, September 2019. ISSN 2050-084X. doi: 10.7554/eLife.48429. URL https://doi.org/10.7554/eLife.48429. Publisher: eLife Sciences Publications, Ltd.

[26] Erika A. Kaske, Cathy S. Chen, Collin Meyer, Flora Yang, Becket Ebitz, Nicola Grissom, Amita Kapoor, David P. Darrow, and Alexander B. Herman. Prolonged Physiological Stress Is Associated With a Lower Rate of Exploratory Learning That Is Compounded by Depression. *Biological Psychiatry: Cognitive Neuroscience and Neuroimaging*, 8(7):703–711, July 2023. ISSN 2451-9022. doi: 10.1016/j.bpsc.2022.12.004. URL https://www.sciencedirect.com/science/article/pii/S2451902222003391.

[27] Zachary P. Kilpatrick, Jacob D. Davidson, and Ahmed El Hady. Uncertainty drives deviations in normative foraging decision strategies. *Journal of the Royal Society, Interface*, 18(180):20210337, July 2021. ISSN 1742-5662. doi: 10.1098/rsif.2021.0337.

[28] Veldon-James Laurie, Akram Shourkeshti, Cathy S. Chen, Alexander B. Herman, Nicola M. Grissom, and R. Becket Ebitz. Persistent Decision-Making in Mice, Monkeys, and Humans, May 2024. URL https://www.biorxiv.org/content/10.1101/2024.05.07.592970v1. Pages: 2024.05.07.592970 Section: New Results.

[29] Rachel S. Lee, Yotam Sagiv, Ben Engelhard, Ilana B. Witten, and Nathaniel D. Daw. A feature-specific prediction error model explains dopaminergic heterogeneity. *Nature Neuroscience*, 27(8):1574–1586, August 2024. ISSN 1546-1726. doi: 10.1038/s41593-024-01689-1. URL https://www.nature.com/articles/s41593-024-01689-1. Publisher: Nature Publishing Group.

[30] Jian Li, Samuel M. McClure, Brooks King-Casas, and P. Read Montague. Policy Adjustment in a Dynamic Economic Game. *PLOS ONE*, 1(1):e103, 2006. ISSN 1932-6203. doi: 10.1371/journal.pone.0000103. URL https://journals.plos.org/plosone/article?id=10.1371/journal.pone.0000103. Publisher: Public Library of Science.

[31] Stefano Palminteri, Emma J. Kilford, Giorgio Coricelli, and Sarah-Jayne Blakemore. The Computational Development of Reinforcement Learning during Adolescence. *PLOS Computational Biology*, 12(6):e1004953, 2016. ISSN 1553-7358. doi: 10.1371/journal.pcbi.1004953. URL https://journals.plos.org/ploscompbiol/article?id=10.1371/journal.pcbi.1004953. Publisher: Public Library of Science.

[32] John M. Pearson, Sarah R. Heilbronner, David L. Barack, Benjamin Y. Hayden, and Michael L. Platt. Posterior cingulate cortex: adapting behavior to a changing world. *Trends in Cognitive Sciences*, 15(4):143–151, April 2011. ISSN 1364-6613. doi: 10.1016/j.tics.2011.02.002. URL https://www.sciencedirect.com/science/article/pii/S1364661311000180.

[33] Herbert Robbins. Some aspects of the sequential design of experiments. *Bulletin of the American Mathematical Society*, 58(5):527–535, 1952. ISSN 0002-9904, 1936-881X. doi: 10.1090/S0002-9904-1952-09620-8. URL https://www.ams.org/bull/1952-58-05/S0002-9904-1952-09620-8/.

[34] Matthew F. S. Rushworth and Timothy E. J. Behrens. Choice, uncertainty and value in prefrontal and cingulate cortex. *Nature Neuroscience*, 11(4):389–397, April 2008. ISSN 1546-1726. doi: 10.1038/nn2066. URL https://www.nature.com/articles/nn2066. Publisher: Nature Publishing Group.

[35] Chaitanya Ryali, Gautam Reddy, and Angela J Yu. Demystifying excessively volatile human learning: A Bayesian persistent prior and a neural approximation. In *Advances in Neural Information Processing Systems*, volume 31. Curran Associates, Inc., 2018. URL https://papers.nips.cc/paper$_{f}iles/paper/2018/hash/78b9cab19959e4af8ff46156ee460c74 - Abstract.html$.

[36] Akram Shourkeshti, Gabriel Marrocco, Katarzyna Jurewicz, Tirin Moore, and R. Becket Ebitz. Pupil size predicts the onset of exploration in brain and behavior. *bioRxiv*, page 2023.05.24.541981, May 2023. doi: 10.1101/2023.05.24.541981. URL https://www.ncbi.nlm.nih.gov/pmc/articles/PMC10245915/.

[37] David W. Stephens and John R. Krebs. *Foraging Theory*. Princeton University Press, 1986. ISBN 978-0-691-08442-8.

[38] Gregory P. Strauss, Michael J. Frank, James A. Waltz, Zuzana Kasanova, Ellen S. Herbener, and James M. Gold. Deficits in positive reinforcement learning and uncertainty-driven exploration are associated with distinct aspects of negative symptoms in schizophrenia. *Biological Psychiatry*, 69(5):424–431, March 2011. ISSN 1873-2402. doi: 10.1016/j.biopsych.2010.10.015.

[39] Robert C Wilson and Anne GE Collins. Ten simple rules for the computational modeling of behavioral data. *eLife*, 8:e49547, November 2019. ISSN 2050-084X. doi: 10.7554/eLife.49547. URL `https://doi.org/10.7554/eLife.49547`. Publisher: eLife Sciences Publications, Ltd.

In this document, we have included all the reviewers' comments in **bold**, our responses in black, and quotes from the revised manuscript in blue. In the manuscript all changes are visible in blue with previous language in ~~red~~.

1 Reviewer #1

My comments the authors replies below. In short, I don't think there is much left for the authors to address, except some very minor textual changes, if at all. Congratulations and a great study!

Thank you for the careful read of our manuscript and helpful comments in the last round of review.

1) Good

We're glad our response addressed this concern.

2) I didn't mean to imply that the model explicitly calculates richness, just that the richness of the environment participants experience, which is correlated with the value of both options, impacts decisions, even if the model doesn't calculate richness itself, but only switches when experience falls under a certain level. What I am saying is that even if you generally do option based learning, having option independent repeated negative reward experiences could make you more exploratory, which is another way of saying that people might have a mixture of option specific learning and "foraging" based exploratory drive when things e.g. get worse. I however, get your point, that I hadn't fully appreciated before, that I should have more carefully dissociated between the environmental richness as the average value of both option which will reduce exploration as both options are "good enough" and the other concept of comparison between experienced richness i.e. value of past choices independent of option, which the model does, unless I am mistaken, track explicitly to compare to their threshold. In short, I agree that the model doesn't really do average value of both options directly, but could be impacted by it (and behaviour seems to be, which is interesting) but the foraging model is directly linked to an exploratory urge when reward experiences go down, which could be seen as an additional drive to explore on top of option specific drives. However, as the authors point out, if this is true, it seems that for their tasks, the reward experience based drive outweighs the option specific one, on average.

We're glad that our revisions clarified the fact that the foraging model does not explicitly calculate the average value of the arms. It may be worth clarifying that the model also does not calculate the value of past choices independently of option because it forgets the value of exploitation when it shifts arms, as described in the text. We agree it would be interesting to explore the utility of incorporating other formulations of richness into these models in future work.

3) I am a bit surprised that the authors couldn't find an openly available dataset, but belief them that they tried. I naively assumed there was more of an commonly shared resource than there appears to be, particularly for such canonical tasks as 2 armed bandits.

We appreciate your understanding and hope that more of these datasets are made publicly available in the future!

4) The authors state "Briefly, the majority of participants did not retain alternative option values and those who did tended to chose the worst alternative–not the best. This is difficult to reconcile with a traditional RL model, although perhaps a good compare- alternatives model could be engineered that is capable of reversing its representation of values from time". This is indeed a very cool finding and contradicts RL models. I find the authors explanation that participants might have a vague sense of not having tried an option for a long time and that being correlated with an option being historical bad, believable for explaining why people actively prefer the worse option, but either way, it does serve a very important piece of information against option specific mechanisms

We appreciate the kind words about this new analysis. It was a great suggestion.

5) I agree with the authors on my misunderstanding of the direction of the unchosen option effect and that it shouldn't play a role! They are correct in pointing out that only an environmental richness mechanism would change their switching based on the value of the unchosen option and only if it maintain such a memory, which the foraging model doesn't. It's nice to see the prediction of the foraging model, a chosen option dominance, come out in a conventional GLM analysis.

Thanks for the suggestion!

6) –

– –

7) I understand that probability space is highly constrained and bounded but disagree that most reward environments tie discriminability and richness together just because our experiments often do. In the real world we experience many environments with differing reward richness independently of the difference in value between options. It just happens to be the case that most researchers are tying themselves to reward probability for learning tasks instead of magnitudes but in magnitude space dissociations between differences and means/richness are very doable even without raising ethical concerns. However, in theirs and many other tasks they are correct about the relationship.

We agree that magnitude manipulations are valuable for decision-making research and would love to see work on decision-making in environments that are effectively unbounded. Our original response was perhaps a little flippant and we agree that it would have been better to focus our argument on the fact that our analyses did not require richness to be independent from discriminability.

8) Good. Minor points are also all addressed.

Thank you kindly!

2 Reviewer #2

We appreciate the authors' thorough revisions and careful consideration of our comments. We find that the additional analyses and revised manuscript address most of our initial concerns. However we do have two main outstanding concerns.

Thanks for the additional feedback. We've addressed the two main points below with additional analyses and have clarified our position on the remaining points.

[1] First, in addressing our comment [1], the authors use the objective (generative) values of the chosen and unchosen options, in both the 2- and 3-armed bandit analyses. This seems like an unfair test of the potential use of the unchosen option's (subjective) value in subjects' decisions because of an asymmetry in how closely correlated the objective and subjective values would be for the chosen versus the unchosen option. Specifically, the objective and subjective values would be more strongly correlated for the chosen option and less so for the unchosen one, precisely because the unchosen option is not sampled much when subjects often repeat their choices. This biases the analysis against finding a role for the unchosen option's value in decisions (and in favor of the compare-to-threshold model). As the authors note, it is not clear which model's subjective value should be used, given the many model variants reported. A good possibility, we suggest, for a subjective value estimate can be derived from an ideal Bayesian learner, where the options' value reflects the sampling history. Note that this model advantageously has no free parameter, it is not fit to participant's behavior. There is no truly 'neutral', a theoretical version of this subjective value. The Bayesian value is conservative insofar as compare-alternatives models provide a closer approximation to an optimal learner, and is hence a strong test of the extent to which subjects may track the unchosen value. If it diverges strongly from the results with generative values currently used, perhaps it is most fair to report both results and discuss this point.

While it is not immediately clear what the ideal Bayesian learner might be in a restless bandit task (which has no known analytical solution for the complete POMDP setting), we considered two alternatives, neither of which changed the central result. First, we considered the parameter-free ideal Bayesian learner that is sometimes used in static bandit tasks. Subjective values estimated with this model were less predictive of choice than objective rewards (likely because the model assumes perfect integration over history, which is better suited to a static, rather than dynamic environment) and the results were unchanged (only chosen value consistently predicted choice and very few participants appeared to compare values). Second, we considered an ideal Bayesian learner for whom unchosen values decayed optimally, but this again did not change the major result (only chosen value consistently impacted choice and very few participants appeared to compare values). Briefly, assuming the learner has knowledge of the specific parameters of the random walks (without directly observing the objective values), we used a continuous approach to the diffusion of the probability density, by integrating a diffusion equation (Fokker-Planck without drift). This integration naturally obtains that the subjective values regress to the prior mean (1/2) due to the boundary conditions, with a decay towards the mean given by the leading order coefficient of the Fourier series that solves the diffusion equation. That is, each trial t that an arm i is not being sampled, the subjective value $v_{i,t}$ should decay following:

$$v_{i,t} \approx \frac{1}{2} + \exp\left(-\frac{\pi^2}{2} p\Delta^2\right)\left(v_{i,t-1} - \frac{1}{2}\right).$$

The variance of the Beta distribution is also updated: $\mathrm{Var}_{i,t} \approx \mathrm{Var}_{i,t-1} + p\Delta^2$, from which the concentration κ of each arm can be updated (the approximation is coming from assuming the drift is independent of the value of the subjective value, which does not hold only at the boundaries). After each trial, we apply this decay to all the arms. Then, following a choice and a reward, we apply the standard Bayesian Beta update for the subjective value $v_{j,t}$ and concentration $\kappa_{j,t}$ for the chosen arm j. This model has indeed no free parameters, but it assumes that the ideal Bayesian learner knows the parameters of the random walks, and has some rather involved calculation for the optimal decay.

These results are reported in a small change to the Results:

These results hold true even when using subjective values estimated via an Ideal Bayesian Learner (IBL) model (see Supplementary Figure S1). Together these results suggest that the value of the previously chosen option was the primary driver of choice, with minimal influence from unchosen option values, consistent with the compare-to-threshold view.

In a new Supplementary Figure:

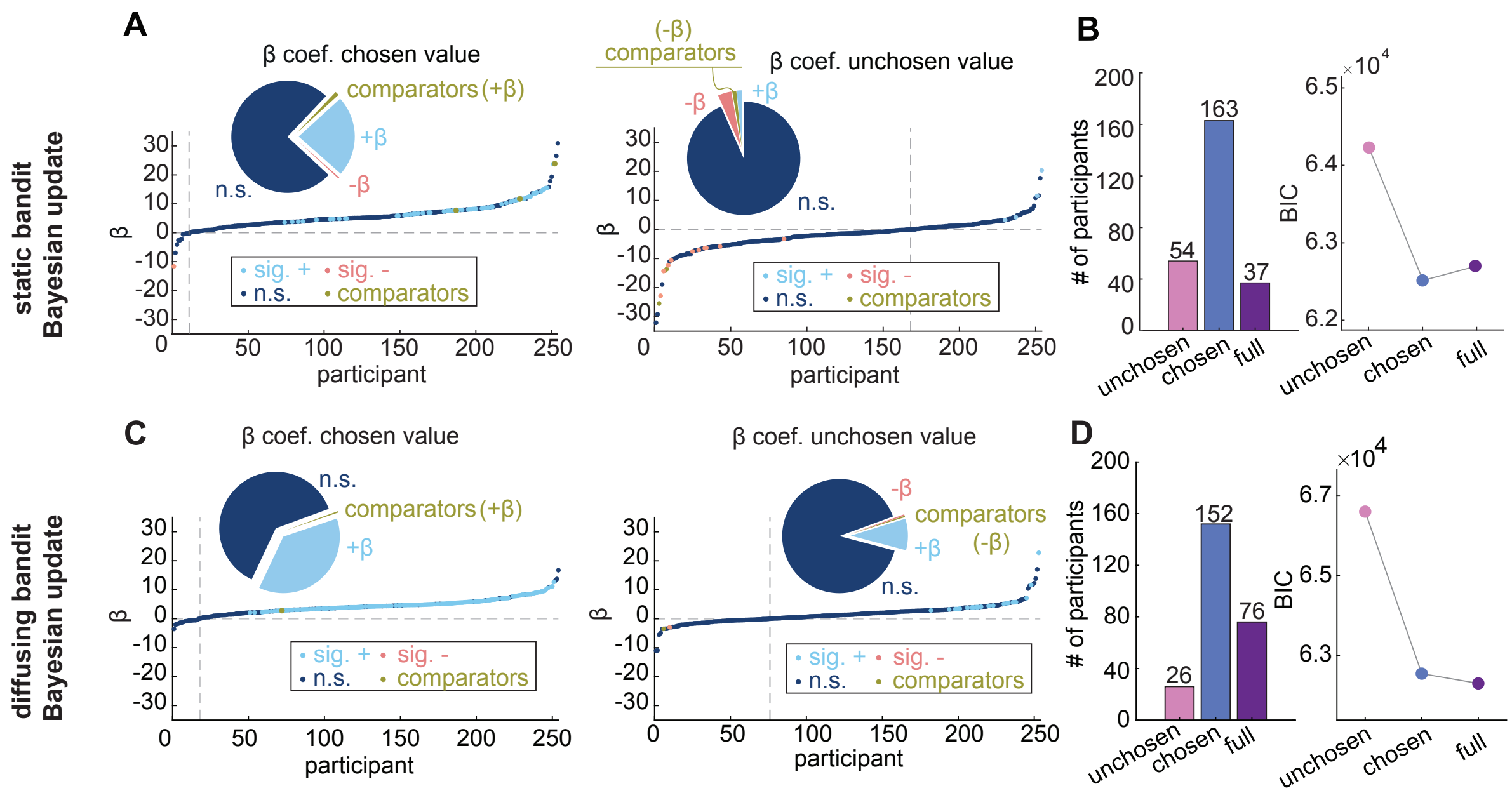

Figure S1: In Figure 1.F-G we used objective reward probabilities as proxies for option values; however, since these are only partially observable to the participants, another approach would be to use an Ideal Bayesian Learner (IBL) to estimate subjective values from the sampling history. The subjective values were estimated either via a parameter-free IBL that perfectly integrates the sampling history over time ("static bandit bayesian update", *A-B*) or via an IBL that knows how the values drift over time (without observing them) and optimal decays the subjective values due to diffusion ("diffusing bandit Bayesian update", *C-D*). See "Choice predictive Generalized Linear Model (GLM)" section of the Methods for more details. **A/C)** Predicting choice from the value of chosen and unchosen options. Beta coefficients for $(V(\text{chosen}_{t-1}))$ (*left*) and $V(\text{unchosen}_{t-1})$ (*right*). Each circle represents a single participant, sorted by coefficient value. The dashed horizontal line is at zero (no effect). Participants with a significant positive betas are light blue, significant negative betas in red, and non-significant betas in dark blue. Gold circles identify "comparators" who had a significant positive weight for the chosen option and negative weight for unchosen option value. Significance is Holm-Bonferroni corrected within predictor ($\alpha = 0.05$). Insets illustrate the proportion of participants in whom each term was significant, colored by sign and identifying all "comparators". **B/D)** (*left*) of BIC for each model ($V(\text{unchosen}_{t-1})$-only, $V(\text{chosen}_{t-1})$-only, and the full model which contains both terms). (*right*) The number of individual participants best fit by each model, based on the Bayesian Information Criterion (BIC).

And a description of the approaches in the Methods:

Objective values were used in Figure 1F-G because these offered the fairest comparison between views. This is because estimating subjective values requires making assumptions about whether the participants are using compare-alternatives or compare-to-threshold computations. That said, it is possible to approximately estimate subjective values through making sensible assumptions about how value could be tracked without fitting any parameters, and repeat the same analyses that we did with objective values.

First, we considered the parameter-free ideal Bayesian learner that is used in static bandit tasks ("static bandit Bayesian update" ; Figure S1). Assuming participants hold ideal beliefs about the probability of reward of each arm (subjective value from now on) via a $Beta(a, b)$ distribution with parameters a, b, following a choice to arm j and a reward r at time t, the parameters of arm j are updated according to

$$a_{j,t+1} = a_{j,t} + r, \quad b_{j,t+1} = b_t + (1 - r), \tag{1}$$

whereas the unchosen arms do not get updated. These equations directly translate to an updated subjective value $v_{i,t+1} = a_{j,t+1}/(a_{j,t+1} + b_{j,t+1})$ and an updated concentration $\kappa_{j,t+1} = a_{j,t+1} + b_{j,t+1}$.

This updating gives weight to all rewards received and ignores the drift, which does not hold optimally in restless arm bandits like our task. Subjective values estimated with this model were less predictive of choice than objective rewards (likely because the model assumes perfect integration over history, which is better suited to a static, rather than dynamic environment) and the results were unchanged (Supplementary Figure S1A-B, only chosen value consistently predicted choice and very few participants appeared to compare values).

Second, we considered an alternative ideal Bayesian learner for whom subjective values decayed optimally ("diffusing bandit Bayesian update" ; Figure S1), but this again did not change the major result (Figure S1C-D, only chosen value consistently impacted choice and very few participants appeared to compare values). Briefly, we assume the learner maintains the same type of belief (Beta distribution for each arm) and updates it knowing that the objective values drift over time. To do this, we assume the learner has knowledge of the specific parameters of the random walks (probability of an upwards or downwards step p and step size Δ), and used a continuous approach to the diffusion of the probability density, by integrating a diffusion equation (Fokker-Planck without drift). Due to the boundary conditions, this integration naturally obtains that the unchosen subjective values should regress to the prior mean (1/2) with maximum uncertainty, with a decay towards the mean given by the leading order coefficient of the Fourier series that solves the diffusion equation. That is, after each trial t the subjective value $v_{i,t}$ of arm i should decay following:

$$v_{i,t+1} \approx \frac{1}{2} + \exp\left(-\frac{\pi^2}{2} p\Delta^2\right)\left(v_{i,t} - \frac{1}{2}\right).$$

The variance of the Beta distribution is also updated: $\mathrm{Var}_{i,t+1} \approx \mathrm{Var}_{i,t} + p\Delta^2$, from which the concentration $\kappa_{i,t+1}$ of each arm can be updated, where the approximation is coming from assuming the drift is independent of the actual value of the subjective value, which does not hold only at the boundaries. Before each trial, we apply this decay to all the arms. Then, following a choice and a reward, we apply the standard Bayesian Beta update (Equation 1) to the chosen arm j to obtain a new subjective value $v_{j,t}$ and concentration $\kappa_{j,t}$.

[2] Second, in their response to our comment [2], the authors implement a version of the dynamic belief updating model that does not allow for an optimism or pessimism bias because P0 is kept fixed. This value should be modeled as a free parameter to capture a persistent prior (where the value of this persistent prior is fitted from choices, just as the threshold in compare-to-threshold is fitted to choices). Therefore, this analysis provided in the response does not currently rule out that (some) subjects may have a persistent prior rather than that they are comparing to threshold.

We've re-fit the DBM model using the method suggested by the reviewer, which does improve model fit but does not change the results: all foraging models still fit better than the DBM. In our revision, we allowed for the persistent prior in the DBM but had fixed the prior's sufficient statistics for simplicity. We have now followed exactly the procedure from Guo et al. 2018, fitting the concentration of the Beta prior at the group level and then the prior mean at the individual level, for both DBM and its exponential-filter approximation (EXP-DBM). We have replaced the previous fits with these new fits. While these fits are now better than standard-RL, they still underperform even standard foraging.

We have removed the mention of using a uniform prior in the Methods, and instead describe this new implementation:

Therefore, both the full DBM and its exponential-filter approximation (EXP-DBM) have in principle 4 free parameters (α, β, a, b). However, the concentration $\kappa = a+b$ of the Beta prior distribution governing γ is usually fit at the group level (Guo et al. 2018). In short, κ measures mainly how peaked this prior distribution is, where more peaked means stronger beliefs about what values γ will take if it is redrawn, values that are governed by the mean $p_0 = a/(a+b)$. We followed this approach and fitted κ at the group level (but separately for DBM and EXP-DBM), and we fitted α, the softmax β and the prior mean p_0 at the individual level, following Guo et al. 2018.

and in the supplementary Figure 3.E

Figure Redacted

Figure S4: **E)** AIC values for additional extensions of traditional RL and foraging-RL models, fits to behavio rfrom Experiment 1. "Richness choice" models incorporate both choice history dependence and richness terms (se eMethods). "exp-DBM" is the exponential filtering approximation to the Dynamic belief model (Ryali et al. 201 8,see Methods), exp is a fully fitted exponential filter (Ryali et al. 2018 and DBM is the Dynamic Belief model as inGuo et al. 2018).

Lastly, in our point [3] concerning model comparison, we recommended that the authors use cross-validation rather than BIC / AIC to avoid assumptions about the appropriate size of the penalty given correlated decision factors. Although we feel we should accept the authors' decision to stick with information criteria because these metrics are so widely used, our point is even more salient now that the authors use both BIC and AIC in different analyses. Specifically, they switch

to BIC in response to point [1]: "Model comparison was evaluated with the Bayesian Information Criterion because we found that the Akaike Information Criterion had an insufficient penalty for the number of parameters (i.e., the full model was generally preferred even when option values were completely randomized)" (p. 25). It would be strongly preferable to use a consistent metric for model fit improvements throughout the paper. We once again urge the authors to consider using cross-validation in comparing fit improvements.

In the previous round of review, this reviewer suggested we emphasize the AIC values when comparing cognitive models, which we consistently did in our revision. It is true that there was single instance in which we use a different metric: comparing GLMs following a control analysis that revealed that only BIC had a sufficiently large penalty to reproduce the ground truth relationships in shuffled data. While BIC was necessary for this single analysis, AIC is standard in the field, was the reviewer's suggestion in the last round, and it would be unreasonable at this point to re-do the cognitive model comparisons throughout the paper because an unrelated analysis required BIC. We were also surprised by the suggestion that the original comment "urge[d] the authors to consider using cross-validation in comparing fit improvements". Perhaps there is a communication issue here, but our reading of the prior comment is (and was) that the reviewer suggested cross-validated log-likelihood only as an alternative to the AIC approach that they also suggested. At this point, the paper uses the standard method to account for differences in the number of parameters and it is not clear from either this comment or the previous comment what value would be added by including cross-validated log-likelihoods or any other exploratory analyses of this type.

2.1 Very minor

: **This is probably a typo, but the sample sizes vary in the reported analyses on p. 25 (i.e., 254 in some places, 258 in others).**

This is not a typo. Parameters couldn't be estimated for participants who never switched but these participants are included in any analysis that didn't rely on model fits. This is specified in the manuscript in several places (i.e., Results L72-73, L349-358, L428-430; Methods, L800-804; Figure 4K).

I had a superficial look at the code base. It seems that: - the revisions are not part of the github repo linked to the submission (last commit 10 months ago) - for the original version, the content is partial and it covers only experiment 1 So it seems that the current repo does not make it possible to reproduce the results of the paper (exhaustively). Maybe the authors can clarify whether the intention is to provide a partial demo, or to release the entire code base in the future.

Thank you for catching that, we forgot to update the repository after the response to revision, but have now uploaded the full code as well as a subset of the data for demonstration. We plan to release the full datasets if the paper is accepted for publication.

3 Reviewer #3

I co-reviewed this manuscript with one of the reviewers who provided the listed reports. This is part of the Nature Communications initiative to facilitate training in peer review and to provide appropriate recognition for Early Career Researchers who co-review manuscripts.

Only had a superficial look together with my co-reviewer. Please refer to his note.

In this document, we have included all the reviewers' comments in **bold**, our responses in black, and quotes from the revised manuscript in blue. In the manuscript all changes are visible in blue with previous language in ~~red~~.

1 Reviewer #1 (Remarks to the Author)

The authors have addressed all my remaining comments.

2 Reviewer #2 (Remarks to the Author)

In their revision, the authors adequately updated the models used in the paper. This extensive model comparison makes the claim of the paper more convincing. I believe there is an error in Fig S4E, as the best model seems to now be the traditional RL model. Some labels for RL model and foraging model are probably swapped by mistake. Regarding the metric used for model comparison, we let the final decision to the authors and editors. We simply note that, from the reader's perspective, having different metrics in the same paper raises the concern of post-hoc selection. An easy fix would be to remove the only BIC value reported in the paper, and replace it with AIC if that's the chosen metric. Regarding our suggestion to use cross-validated likelihood, it is because unlike BIC and AIC, this method does not make assumption about how to penalize the model based on a number of free parameters, it is thus more general.

Thank you for the careful read of our manuscript and helpful comments in the last round of review. Thank you also for catching the error in Figure S4e, the labels were indeed swapped and have been corrected. As we previously explained, AIC has an insufficient penalty for this specific analyses. There are many solutions to the bias variance tradeoff each with their pros and cons.

3 Reviewer #2 (Remarks on code availability)

The code base remains largely incomplete, although it has been updated recently. In particular I don't find the analyses for Experiment 2 (if it's there, it should be better documented in the readme) and several files and paths are hard coded on the author's computer and not available (e.g. I could not find volatilityLMH_newstates2025.mat that is required for unchosenOption_exploreStrategy.m)

Following the reviewer's recommendations, we have updated the README file to clearly outline the location of each analysis. Additionally, the file referenced by the reviewer is a dataset, which has been made publicly available on Figshare.

4 Reviewer #3 (Remarks to the Author)

I co-reviewed this manuscript with one of the reviewers who provided the listed reports. This is part of the Nature Communications initiative to facilitate training in peer review and to provide appropriate recognition for Early Career Researchers who co-review manuscripts.
